# U-Limb: A multi-modal, multi-center database on arm motion control in healthy and post-stroke conditions

**DOI:** 10.1093/gigascience/giab043

**Published:** 2021-06-18

**Authors:** Giuseppe Averta, Federica Barontini, Vincenzo Catrambone, Sami Haddadin, Giacomo Handjaras, Jeremia P O Held, Tingli Hu, Eike Jakubowitz, Christoph M Kanzler, Johannes Kühn, Olivier Lambercy, Andrea Leo, Alina Obermeier, Emiliano Ricciardi, Anne Schwarz, Gaetano Valenza, Antonio Bicchi, Matteo Bianchi

**Affiliations:** Research Center “Enrico Piaggio” and Dipartimento di Ingegneria dell’Informazione, University of Pisa Largo Lucio Lazzarino 1, 56122 Pisa, Italy; Soft Robotics for Human Cooperation and Rehabilitation, Fondazione Istituto Italiano di Tecnologia, Via Morego 30, 16163 Genova, Italy; Research Center “Enrico Piaggio” and Dipartimento di Ingegneria dell’Informazione, University of Pisa Largo Lucio Lazzarino 1, 56122 Pisa, Italy; Soft Robotics for Human Cooperation and Rehabilitation, Fondazione Istituto Italiano di Tecnologia, Via Morego 30, 16163 Genova, Italy; Research Center “Enrico Piaggio” and Dipartimento di Ingegneria dell’Informazione, University of Pisa Largo Lucio Lazzarino 1, 56122 Pisa, Italy; RSI - Chair of Robotics and Systems Intelligence, Munich School of Robotics and Machine Intelligence, Technical University Munich (TUM), Heßstr. 134, 80797 München, Germany; MoMiLab Research Unit, IMT School for Advanced Studies Lucca, Piazza S. Francesco 19, 55100 Lucca, Italy; Division of Vascular Neurology and Neurorehabilitation, Department of Neurology, University of Zurich, Frauenklinikstrasse 26, 8006 Zürich, Switzerland; RSI - Chair of Robotics and Systems Intelligence, Munich School of Robotics and Machine Intelligence, Technical University Munich (TUM), Heßstr. 134, 80797 München, Germany; Laboratory for Biomechanics and Biomaterials (LBB), Department of Orthopaedic Surgery, Hannover Medical School, L384, 30625 Hannover, Germany; Rehabilitation Engineering Laboratory, Institute of Robotics and Intelligent Systems, Department of Health Sciences and Technology, CLA H 1.1 Tannenstrasse 3, 8092 Zurich, Switzerland; RSI - Chair of Robotics and Systems Intelligence, Munich School of Robotics and Machine Intelligence, Technical University Munich (TUM), Heßstr. 134, 80797 München, Germany; Rehabilitation Engineering Laboratory, Institute of Robotics and Intelligent Systems, Department of Health Sciences and Technology, CLA H 1.1 Tannenstrasse 3, 8092 Zurich, Switzerland; MoMiLab Research Unit, IMT School for Advanced Studies Lucca, Piazza S. Francesco 19, 55100 Lucca, Italy; Laboratory for Biomechanics and Biomaterials (LBB), Department of Orthopaedic Surgery, Hannover Medical School, L384, 30625 Hannover, Germany; MoMiLab Research Unit, IMT School for Advanced Studies Lucca, Piazza S. Francesco 19, 55100 Lucca, Italy; Division of Vascular Neurology and Neurorehabilitation, Department of Neurology, University of Zurich, Frauenklinikstrasse 26, 8006 Zürich, Switzerland; Research Center “Enrico Piaggio” and Dipartimento di Ingegneria dell’Informazione, University of Pisa Largo Lucio Lazzarino 1, 56122 Pisa, Italy; Research Center “Enrico Piaggio” and Dipartimento di Ingegneria dell’Informazione, University of Pisa Largo Lucio Lazzarino 1, 56122 Pisa, Italy; Soft Robotics for Human Cooperation and Rehabilitation, Fondazione Istituto Italiano di Tecnologia, Via Morego 30, 16163 Genova, Italy; Research Center “Enrico Piaggio” and Dipartimento di Ingegneria dell’Informazione, University of Pisa Largo Lucio Lazzarino 1, 56122 Pisa, Italy

**Keywords:** motion control, upper limb, stroke, human kinematics, electromyography, electro-encephalography, functional magnetic resonance imaging, Virtual Peg Insertion Test

## Abstract

**Background:**

Shedding light on the neuroscientific mechanisms of human upper limb motor control, in both healthy and disease conditions (e.g., after a stroke), can help to devise effective tools for a quantitative evaluation of the impaired conditions, and to properly inform the rehabilitative process. Furthermore, the design and control of mechatronic devices can also benefit from such neuroscientific outcomes, with important implications for assistive and rehabilitation robotics and advanced human-machine interaction. To reach these goals, we believe that an exhaustive data collection on human behavior is a mandatory step. For this reason, we release U-Limb, a large, multi-modal, multi-center data collection on human upper limb movements, with the aim of fostering trans-disciplinary cross-fertilization.

**Contribution:**

This collection of signals consists of data from 91 able-bodied and 65 post-stroke participants and is organized at 3 levels: (i) upper limb daily living activities, during which kinematic and physiological signals (electromyography, electro-encephalography, and electrocardiography) were recorded; (ii) force-kinematic behavior during precise manipulation tasks with a haptic device; and (iii) brain activity during hand control using functional magnetic resonance imaging.

## Background

An open access approach to experimental data on human sensorimotor behavior has become extremely popular in the recent years, not only for neuroscience and clinics, but also for devising new design and control guidelines in robotics. This interest has been strengthened by the widespread adoption of deep learning techniques for analyzing human movements, which has fostered the translation of neuroscientific observations for robot control, design, and planning [1]. In the literature, it is possible to find a number of datasets focusing on human loco-manipulation, in which data were acquired using different acquisition modalities, ranging from RGB cameras to optical markers and electromyographic (EMG) techniques [2–11]. Among them, it is worth mentioning the KIT Whole-Body Human Motion Database (https://motion-database.humanoids.kit.edu/), a comprehensive motion capture database of whole-body human motion [12], and the NinaPro database, which consists of surface electromyography (sEMG) data acquired from 67 intact participants and 11 participants with amputations, who were asked to perform 50 different movements [13, 14].

Although these datasets represent an important tool for improving the knowledge on the neuroscientific aspects underpinning motor generation and control in humans, their focus was limited to specific acquisition modalities or anatomical parts. Looking at the upper limb as a whole (i.e., considering the entire kinematic chain), there is poor or no evidence of databases where multi-modal and multi-center data have been collected. Furthermore, disease conditions, such as post-stroke participant data, are rarely considered. To the best of our knowledge, the only example in the literature is the Toronto Rehab Stroke Pose Dataset [15], which consists of upper body 3D poses recorded through Microsoft Kinect Sensors of 9 post-stroke patients and 10 healthy participants performing a set of tasks using an upper limb rehabilitation robot.

In this work, we strive to release an exhaustive collection of data related to the neural and local control of the upper limb musculoskeletal system, the U-Limb dataset (consisting of 91 able-bodied and 65 post-stroke participants acquired), with the aim of describing upper limb motions in both healthy (i.e., participants with no known history of neurological or physical issue) and disease conditions. The 2 great novelties of this work are (i) multi-modality and (ii) multi-centricity; i.e., data were acquired at different research and clinical centers, using shared and integrated protocols. The choice of multi-centricity is also motivated by the need to guarantee the robustness of the collected data. At the same time, multi-modal acquisitions can offer a privileged point of view to unveil different yet related aspects of human upper limb motor control. For example, kinematic data can shed light on the workspace and the phenomenological characteristics of healthy movements, while offering a benchmark to comparatively evaluate the severity of the motor impairment. In this regard it is worth underlining that the postural data contained in the U-Limb dataset, which are related to daily living activities, refer to both able-bodied and post-stroke participants. These participants underwent the same experimental protocol, which also includes sEMG and electro-encephalography (EEG) measurements, to provide information on the level of muscular tone and brain connectivity, respectively, thus offering a unique opportunity to identify quantitative tools for informing and evaluating rehabilitative outcomes. Furthermore, these different types of information can be used to analyze whether and to what extent the abundance of healthy sensorimotor degrees of freedom (DoF) of the upper limb is organized in low-dimensional representations, or synergies, whose study has received a lot of attention in the past decade. More specifically, the main focus of these studies has been on human hands, and it has driven important technological translational outcomes for engineering, assistive and rehabilitation robotics, and advanced human-machine interaction [16]. In parallel to daily living activities, we also report on data that target the observation of precise force-kinematic coordination in manipulation tasks with a robotic device, and functional magnetic resonance imaging (fMRI) data on hand fine motor control in imagined, performed, and observed manipulation tasks. In this way, we can provide a comprehensive description of the neuroscientific aspects underpinning motion generation along the whole upper limb kinematic chain, highlighting the different aspects (kinematic, muscular, neural, dynamic) of this process.

These data were collected within the recently ended H2020 EU-funded Project SoftPro, whose goal was to move from the understanding of the theoretical bases of sensorimotor control of the upper limb to produce a strong impact in different fields of research, clinical practice, and technology. More details on data organization and collection are provided in the following sections.

## Data Description

During the SoftPro Project, we collected different sets of physiological and kinematic data on the human upper limb, in both healthy and disease conditions. The latter refer to post-stroke participants, whose clinical characteristics are reported later in the text.

Data acquisition followed 3 experimental protocols, i.e., the lists of tasks thath the participants were asked to perform during the acquisition:

- daily living activities, hereinafter referred to as SoftPro protocol;
- hand grasping and control for the fMRI experiments, hereinafter referred to as fMRI protocol;
- coordination of arm and hand movements as well as grasping forces during a virtual, goal-directed object manipulation task performed with a haptic device, hereinafter referred to as VPIT (virtual peg insertion test) protocol.

The details of each protocol are reported in the dedicated section and subsections.

Data collection was organized to be multi-center and to encompass different acquisition and signal modalities. More specifically, the contributors to the generation of these datasets are University of Pisa (UP), Istituto Italiano di Tecnologia (IIT), Hannover Medical School (MHH), Technical University of Munich (TUM), University of Zurich (UZH), Swiss Federal Institute of Technology in Zurich (ETHZ), and IMT School for Advanced Studies Lucca (IMT). Data types are as follows:

- kinematic recordings (optical marker positions or inertial measurement unit [IMU]-based reconstructions of angular values through commercial sensing systems), hereinafter referred to as KIN data;
- EMG signals, hereinafter referred to as EMG data;
- EEG signals, hereinafter referred to as EEG data;
- electrocardiography (ECG) signals, hereinafter referred to as ECG data;
- fMRI, hereinafter referred to as fMRI data;
- kinematic end-effector, grasping force, and haptic interaction data from the VPIT protocol, hereinafter referred to as VPIT data.

The details of each experimental acquisition procedure are reported in the dedicated following section.

The information on the able-bodied participants (sex, mean age, handedness) who took part at the experimental sessions is briefly summarized in the following:

- Group A: 39 healthy participants, 17 female, age 26.6 ± 4.2 years, all right-handed, recorded by UP, participants were tested on the right arm;
- Group B: 20 healthy participants, 8 female, age 46.77 ± 15.25 years, 18 right-handed, recorded by MHH, participants were tested on their dominant hand;
- Group C: 5 healthy participants, 2 female, age 59.15 ± 15.85 years, recorded by UZH, participants were tested on both arms;
- Group D: 6 healthy male participants, age 29.17 ± 5.91 years, all right-handed, recorded by TUM, participants were tested on the right arm;
- Group E: 27 healthy participants, divided in 3 independent groups of 9 participants (5 female) each, all right-handed. Execution experiment: age 29 ± 3 years, imagery experiment: age 27 ± 6 years, observation experiment: age 25 ± 2 years, all recorded by IMT.

The details of the post-stroke participants involved in the experiments are reported as follows:

- Group α: 20 post-stroke participants, 5 female, age 61.00 ± 10.69 years, 11 right-arm affected, recorded by UZH, participants were tested on both arms. Note that these participants are a subset of Group γ and that the IDs are coherent between the 2 datasets. Note also that these participants were collected with the same experimental protocol and by the same experimenter as Group C, and these may serve as control group when using data of Group α.
- Group β: 20 post-stroke participants, of which 6 female, age 49.88 ± 16.92 years, 12 right-arm affected, recorded by MHH, participants were tested on the impaired arm. Note that these participants were collected with the same experimental protocol and by the same experimenter as Group B, and these may serve as control group when using data of Group α.
- Group γ: 27 post-stroke participants, 14 female, age 59.0 ± 10.93 years, 26 right-handed, recorded by ETHZ. Participants were tested on both arms. Because both the unimpaired and impaired arm were tested in Group γ, we suggest the user to consider the first set of data as control group with respect to the second.

An overview of all the data reported in this publication is finally provided in Table 1, where we also indicate the contributor and the details of the ethical committee that gave the approval to acquire and share these data in an anonymous form. Additional details on the cohort of participants enrolled for each group are collected in Table 2. All participants gave written informed consent before the start of the experiment.

**Table 1:** Details on the groups of participants enrolled in the studies

ID	Type	Group	Protocol	Contributor	Ethical Committee Approval No.
H_1_	KIN	A	SoftPro	UP	1072-2016
H_2_	EEG	A	SoftPro	UP	1072-2016
H_3_	ECG	A	SoftPro	UP	1072-2016
H_4_	KIN	B	SoftPro	MHH	3364-2016
H_5_	EMG	B	SoftPro	MHH	3364-2016
H_6_	KIN	C	SoftPro	UZH	BASEC-ID 2016-02075
H_7_	KIN	D	SoftPro	TUM	EV LUH 05/2016
H_8_	EMG	D	SoftPro	TUM	EV LUH 05/2016
H_9_	EEG	D	SoftPro	TUM	EV LUH 05/2016
H_10_	fMRI	E	fMRI	IMT	1616/2003 (amended), 1072/2016
P_1_	KIN	α	SoftPro	UZH	BASEC-ID 2016-02075
P_2_	KIN	β	SoftPro	MHH	3364-2016
P_3_	EMG	β	SoftPro	MHH	3364-2016
P_4_	VPIT	γ	VPIT	ETHZ	EKNZ-2016-02075, EK2017-00398

IDs *H_x_* refer to healthy participants, while IDs *P_x_* to participants with disease. All the experiments were carried out in accordance with the principles of the Declaration of Helsinki, and approved by the local institutional research ethical committees. All participants gave written informed consent before the start of the experiment. Experiments performed at UP were approved by the Ethics Committee of the Area Vasta Nord-Ovest Toscana, Italy; experiments performed at MHH were approved by the Ethics Committee of Hannover Medical School; experiments performed at UZH were approved by the Cantonal Ethics Committee Northwest and Central Switzerland; experiments performed by TUM were approved by the Ethics Committee of Leibniz Universität Hannover, Germany; experiments performed at IMT were approved by the Ethics Committee of the Area Vasta Nord-Ovest Toscana, Italy; experiments performed at ETHZ were approved by the Ethics Committee of ETH Zurich. Note that different ID of this table may correspond to the same group of participants. For example, participants of Group A were a cohort of 39 healthy participants who performed 1 single experiment while kinematics, EEG, and ECG recordings were simultaneously recorded (IDs H_1_, H_2_, H_3_, respectively).

**Table 2:** Details on the different populations included in this article

Group	Contributor	Participant No.	Mean age, y	M/F	Handedness R/L	Mean FMA score
A	UP	39	26.6 ± 4.2	22/17	39/0	N/A
B	MHH	20	46.8 ± 15.3	12/8	18/2	N/A
C	UZH	5	59.2 ± 15.9	3/2	5/0	N/A
D	TUM	6	29.2 ± 6	6/0	6/0	N/A
E	IMT	27	27.0 ± 2	22/5	27/0	N/A
α	UZH	20	61.0 ± 10.7	15/5	19/1	17.8 ± 2.1 (≤66)
β	MHH	20	49.9 ± 16.9	14/5	12/8	17.8 ± 2.1 (≤20)
γ	ETHZ	27	59.0 ± 10.9	13/14	26/1	46.6 ± 9.3 (≤66)

For each group of participants (for details on the modalities see Table 1), we report here the contributor, the number of participants, their mean age, the sex balance, the handedness (right vs left handed), and the mean stroke severity in terms of FMA score.

### Details on the severity level of post-stroke participants

Specific details on the level of impairment for participants of Groups α, β, and γ are reported in the accompanying files included in the corresponding dataset directory.

### Data type

#### KIN data

Kinematic data encompass both (i) optical marker positions and (ii) IMU-based angular reconstructions during the implementation of the SoftPro protocol. Regarding (i), we collected different sets of data containing the measurements of 3D optical marker coordinates related to the upper limb movements. Although different across laboratories, the placement of markers is always sufficient—with a certain redundancy—to enable the estimation of upper limb movements and the identification of a minimum set of DoF, relying on a shared kinematic model (see, e.g., [17]). In the following we provide additional details for each dataset, referring to the ID reported in Table 1.

**Figure 1:**
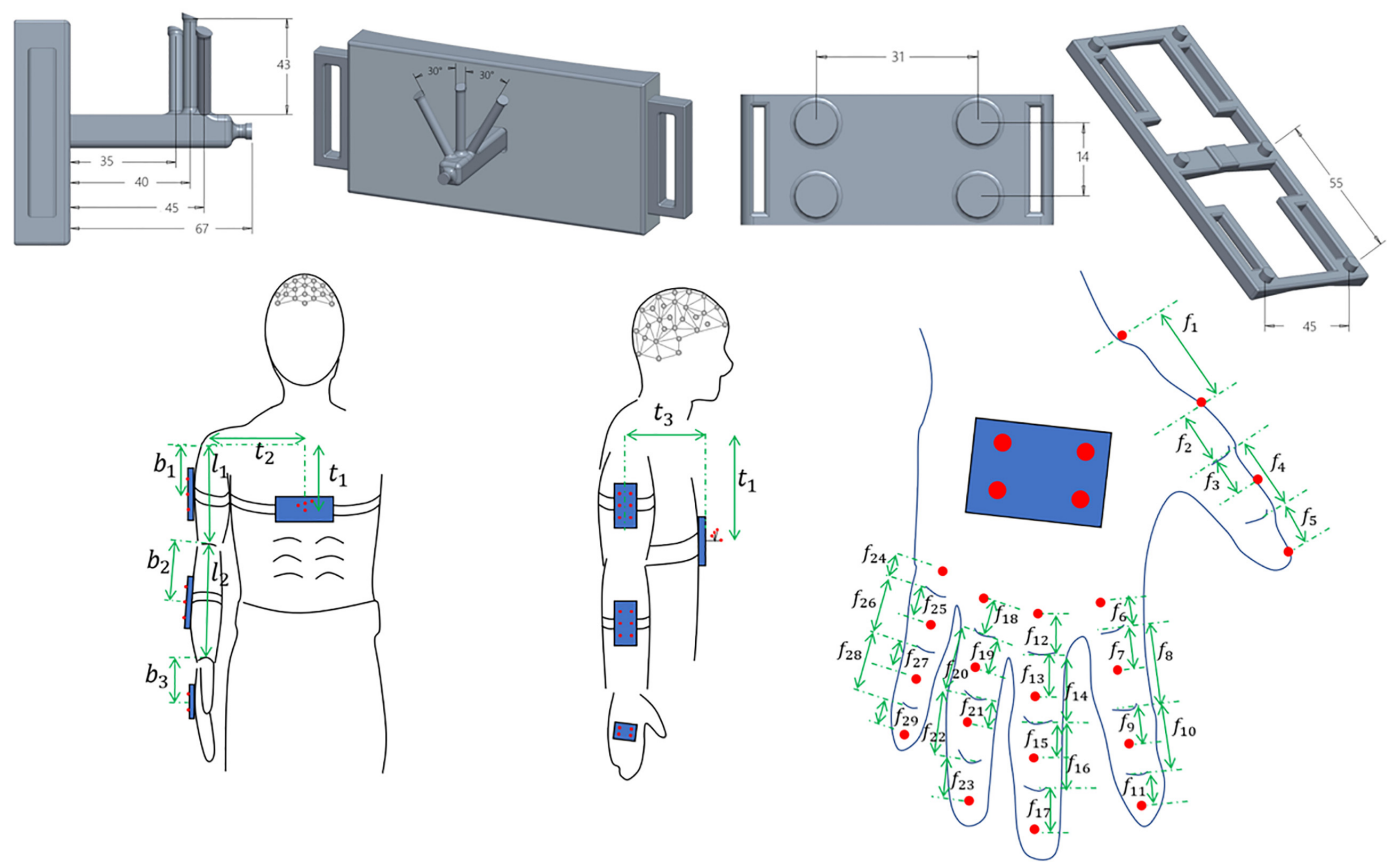
Anatomical placement of active markers, and details on the marker support used for the experiments at UP. Numerical values on the dimensions of marker support are reported in millimeters.

**Figure 2:**
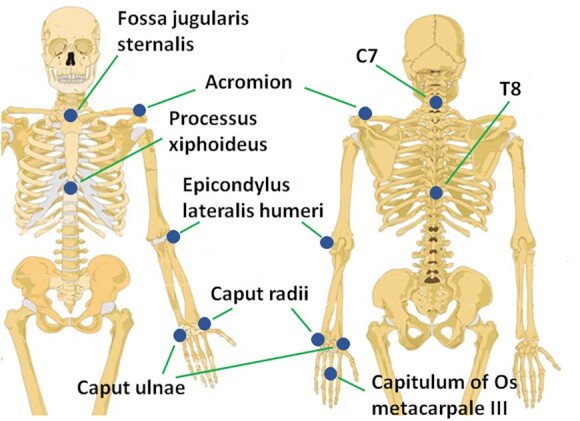
Anatomical landmarks (blue circles) that define the marker placement for the experiments performed at TUM (H_7_). C7 and T8 refer to the seventh cervical and the eighth thoracic vertebrae, respectively.

H_1_—Participants of Group A were enrolled in this study. Twenty active markers were placed on rigid supports fastened on arm links. In particular, 4 markers were placed on the chest, 6 markers on the arm, 6 markers on the forearm, and 4 markers on the hand dorsum. In addition, 20 active markers were also placed on the participant’s fingers to track hand movements. Marker 3D position was recorded via a PhaseSpace motion capture system. Marker locations and ID are reported in Fig. 1. Participant-specific physical distances between groups of markers and kinematic landmarks are provided in the data folder. See also [17–19] for further details.
H_4_—Participants of Group B were involved in this study. Arm movements were tracked trough 21 passive markers fastened on arm skin. Marker trajectories were captured using an optical infrared motion-capturing system based on 12 MX-cameras controlled by Nexus software, Version 1.8.5 (Vicon Motion System Ltd., Oxford, UK) at a sampling rate of 200 Hz. The marker placement and their IDs are given in Fig. 2.
H_6_—Participants of Group C were involved in this study. The data were recorded with a full-body worn IMU-based system sensor suit (Awinda, Xsens technologies B.V., Enschede, The Netherlands). The system consists of 17 IMUs placed symmetrically on predefined body positions and fixed with Velcro straps and a close-fitting t-shirt. The IMUs provide 3D angular velocity using rate gyroscopes, 3D acceleration using accelerometers, 3D earth magnetic field using magnetometers, as well as atmospheric pressure using the barometer in an operating frequency 2,405−1,475 MHz. Then, proprietary software was used to reconstruct the time-varying angular deviation (roll-pitch-yaw) between subsequent IMUs. For additional details see the user’s manual [20].
H_7_—Participants of Group D were involved in this study. Upper body and shoulder-arm movements were tracked using 9 passive markers, recorded using a Vicon MXT10s (Vicon Motion Systems Ltd, UK, 500 Hz) system with 8 cameras. See Fig. 2 for details of marker placement.

**Figure 3:**
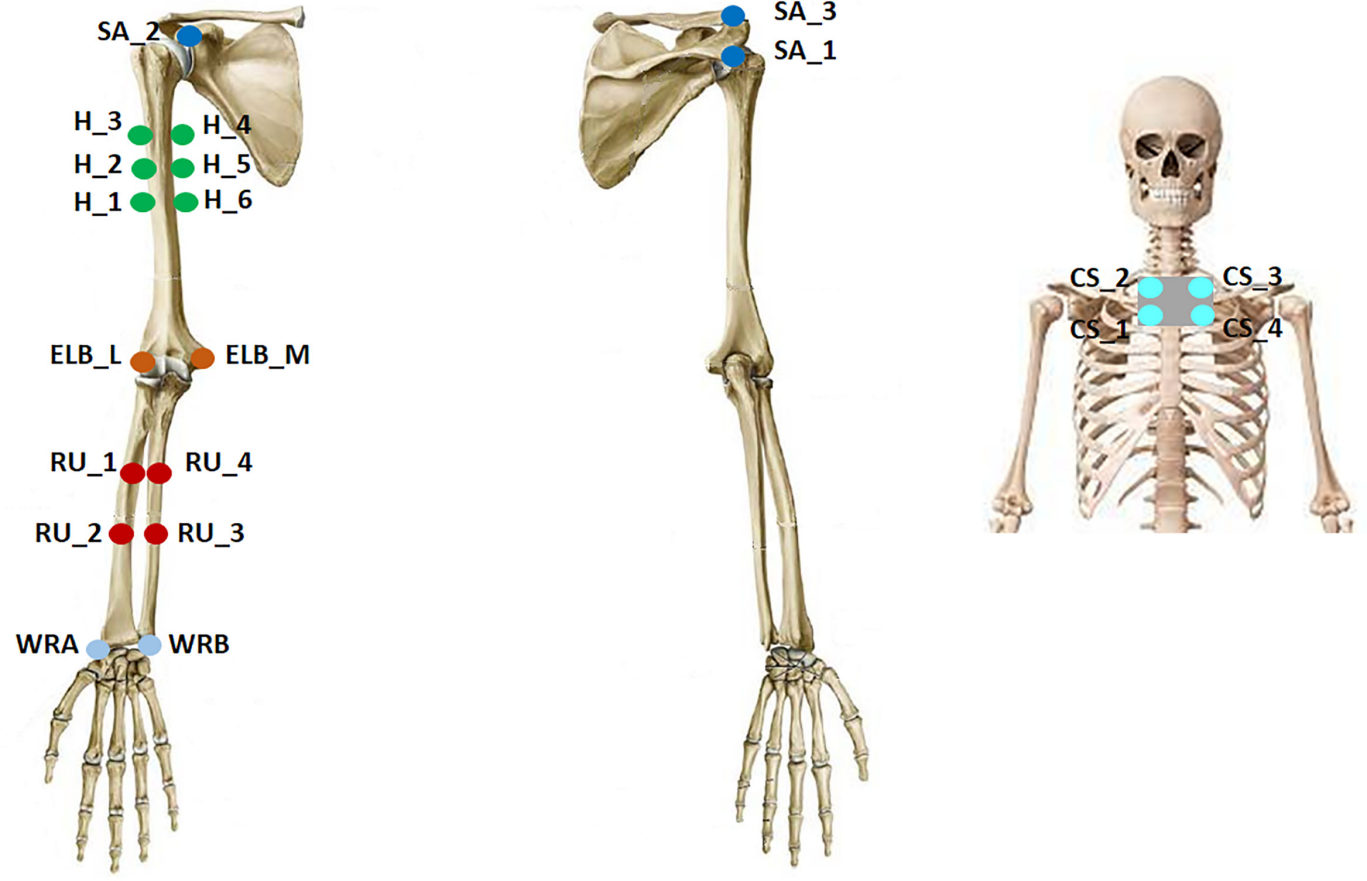
Marker placement used during the experiments performed at MHH (H_4_).

To enable the analysis of the effects of stroke conditions in upper limb kinematics (i.e., movements) we recorded the motion of participants in disease conditions. More specifically:

P_1_—Participants of Group α were enrolled in this study. Arm movements were recorded using the Xsens MVN Awinda system (same set-up of *H*_6_). This consists of 17 IMU sensors, placed on the body limbs and trunk, and of a software tool that allows data collection with a frequency of 60 Hz and reconstructs the joint angular values in time, starting from acceleration signals. Part of these data have been used in [21], to which the reader can refer for further details.
P_2_—Participants of Group β were involved in this study. Arm movements were tracked trough 21 passive markers fastened on arm skin. Marker placement and data acquisition were the same used for Group H2 (see Fig. 3).

#### EMG data

**Figure 4:**
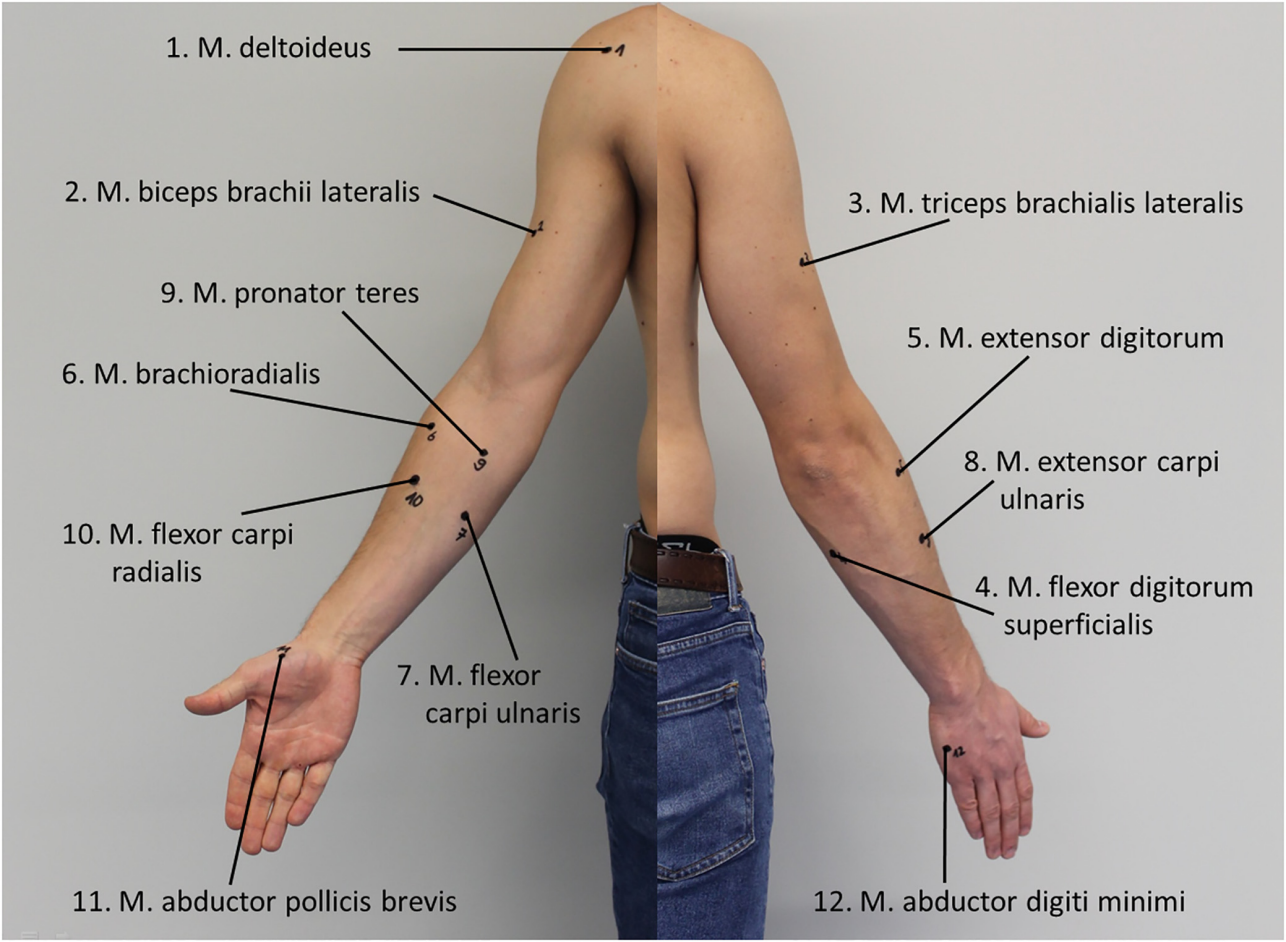
Placement of EMG sensors following SENIAM guidelines.

Muscular data were recorded during experiments ID H_5_, H_8_, and P_3_. More specifically:

H_5_—Participants of Group B were enrolled in this study. A wireless sEMG system (Trigno Delsys, Inc., Natick, MA, USA) was used to measure the activity of 12 upper arm and forearm muscles with 2,000 fps (Table 3, see also Fig. 4). Mini sensors were used for smaller muscles (No. 9−12) to reduce cross-talk artifacts. The 12 bipolar electrodes were placed following the Surface EMG for Non-invasive Assessment of Muscles (SENIAM) guidelines.
H_8_—Participants of Group D were involved in this study. Data were collected using a Refa system (TMSi, Oldenzaal, The Netherlands) with 29 bipolar channels. The 29 × 2 microelectrodes were placed, following the SENIAM guidelines [22], on the muscles reported in Table 5.
P_3_—Participants of Group β were involved in this study. The experimental framework used is the same as in H_5_ (see Table 3).

**Table 3:** List of 12 muscles recorded during the experiments at MHH

Electrode No.	Muscle
1	M. Deltoideus pars clavicularis (DC)
2	M. Biceps brachii (BB)
3	M. Triceps brachii (TB)
4	M. Flexor digitorum superficialis (FDS)
5	M. Extensor digitorum (ED)
6	M. Brachioradialis (BR)
7	M. Flexor carpi ulnaris (FCU)
8	M. Extensor carpi ulnaris (ECU)
9	M. Pronator teres (PT)
10	M. Flexor carpi radialis (FCR)
11	M. Abductor pollicis brevis (APB)
12	M. Abductor digiti minimi (ADM)

#### EEG data

**Figure 5:**
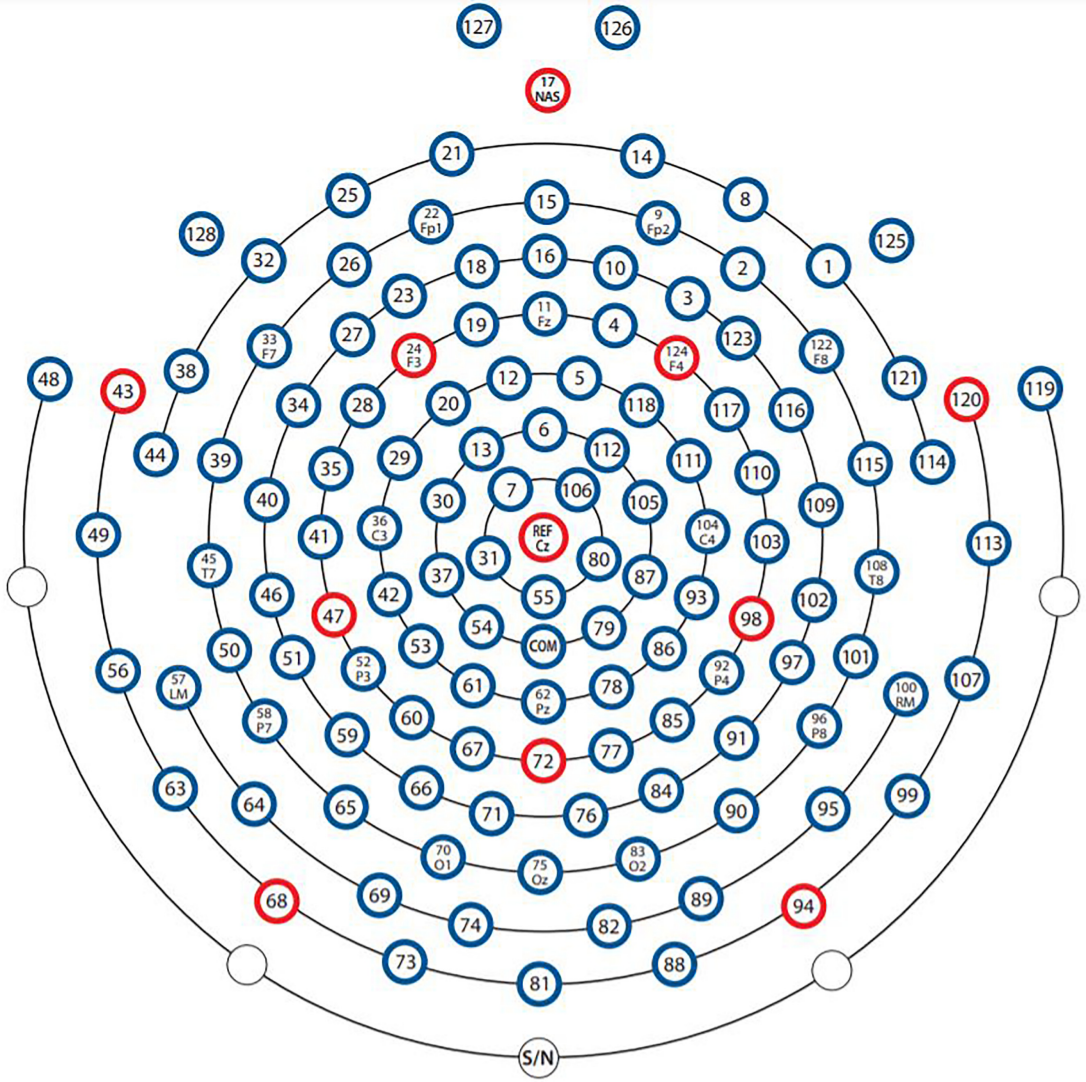
A schematic representation of HydroCel Geodesic Sensor Net (HCGSN-128) channel locations.

Cortical activity was recorded during the experiments ID H_2_ and H_9_. More specifically:

H_2_—Participants of Group A were enrolled in this study. Continuous EEG was recorded using a 128-channel Geodesic high-density EEG System (Electrical Geodesics Inc., Eugene, OR, USA) through a pre-cabled HydroCel Geodesic Sensor Net (HCGSN-128), sampling rate of 500 Hz with the vertex as online reference; sensor-skin impedances were maintained at <5–10 kΩ for each sensor. The “ground” sensor on the Net is an “isolated common,” which means that it is tied to the zero level, or common, of the isolated amp circuit’s power supply. A schematic representation of channel locations is provided in Fig. 5. These data were used for the analyses reported in [23–26], to which the reader is invited to refer for further technical details.
H_9_—Participants of Group D were involved in this study. An actiCHamp active EEG electrode net of 32 unipolar channels (Brain Products GmbH, Gilching, Germany)—which corresponds to the 10-20 system [27]—was used at 10 kHz to record brain activity.

#### ECG Data

Heart electrical activity was recorded during the experiment ID H_3_. More specifically:

H_3_—Participants of Group A were enrolled in this study. Continuous ECG was recorded using the Polygraph Input Box (PIB), the EGI’s physiological measurement Geodesic System (Electrical Geodesics Inc., Eugene, OR, USA). It allows the simultaneous measurement of peripheral nervous system activity and EEG; indeed the acquisition was performed together with experiment IDs H_2_ and H_1_. The PIB includes a bipolar channel input for the measurement of ECG. The input box accommodates the most common sensor connector (the 1.5-mm female safety connector) that is used in both clinical and research settings. Signals were acquired with a sampling rate of 500 Hz, applying 2 standard ECG sensors, the first to the lower left ribcage and the second to the upper right collarbone/clavicle, in accordance with the constructor design.

#### VPIT Data

Kinematic and haptic interaction data were transferred through a FireWire connection from the end-effector to a personal computer. Grasping force data were recorded through an NI (National Instruments, Austin, TX, USA) Data Acquisition Card. The virtual reality environment of the VPIT was implemented in C++ and OpenGL. All data were sampled at 1 kHz. Missing data segments, which occurred owing to a delayed communication of the C++ software, of ≥50 samples were linearly interpolated. Furthermore, the sensor readings were low-pass filtered with a zero-phase Butterworth filter of second order and 10 Hz cut-off frequency. Because the VPIT comprises multiple movement phases with different characteristics, a temporal segmentation of the continuous data streams is required to select specific parts of the movements that are relevant to describe impairments in the targeted sensorimotor functions. In more detail, the “transport" (ballistic movement after picking up a peg) and “return" (ballistic movement after releasing a peg in a hole) phases focus especially on the gross movements of the task. The start and end of these phases were identified by the moment the cursor velocity increased above and decreased below 5% of peak velocity, respectively. To quantify fine target adjustments when reaching for a target or hole, the data were segmented into the “peg approach" and “hole approach" phases. Last, the grasping force data were additionally divided into the “force buildup" and “force release" phases. These periods were detected by first identifying the largest maximum/minimum in the force rate profile and subsequently quantifying when the force rate decreased below and increased above 10% of the maximum/minimum force rate. More details about the data processing can be found in previous work [28].

#### fMRI

All fMRI data were acquired using a Philips Ingenia 3-Tesla scanner, with a 12-channel head phased array coil. Data consisted of anatomical and functional images. For anatomical images, a MP-RAGE sequence was acquired, with TR = 7 ms, TE = 3.17 ms, flip angle = 9°, field of view = 224 × 224 mm, 156 sagittal slices, voxel size = 1 × 1 × 1 mm. To acquire functional images, a Gradient-Echo EPI sequence was used, with TR = 200 ms, TE = 30 ms, flip angle = 75°, SENSE acceleration, factor = 2.5, field of view = 256 × 256 mm, 38 interleaved axial slices, acquisition voxel size = 3 × 3 × 3 mm. Images were reconstructed with a 128 × 128 matrix, and reconstructed voxel size was 2 × 2 × 3 mm. The top-to-bottom extent along the *z*-axis was 114 mm; this ensured total brain coverage, excluding part of the cerebellum. Functional runs comprised 4 additional dummy volumes, discarded by the scanner and not transferred.

Structural images were anonymized with mri_deface [29] to remove any anatomical detail that can allow participants’ identification. For functional MRI, the initial stages of pre-processing and the estimation of single-participant BOLD responses were performed using AFNI [30] and FSL 5.01 [31]. First, all fMRI data underwent removal of signal spikes, temporal realignment of slices, rigid-body registration to the mean image of the first run, and estimation of the 6 motion parameters. Motion spikes were then estimated as time-points exceeding 0.5 mm of framewise displacement (FD) [32]; iterative spatial smoothing up to 4 mm full width at half-maximum was subsequently performed, and the signal of each run was expressed as a percentage of the mean. Afterwards, stimulus-evoked fMRI responses were estimated for each task using a general linear model: the onsets of the 5 repetitions of each stimulus were entered into the model as regressors of interest, and the 6 motion parameters plus the raw value of the FD metric and polynomial trends up to the fourth order were used as regressors of no interest. The 5 repetitions of each stimulus were combined; for the execution and imagery experiments, we modeled the entire stimulation period (0–16 seconds) with 9 tent functions peaking at 2.5 seconds. The average t-score maps from the fifth, sixth, and seventh functions, which covered activity from 2 to 6 seconds after movement onset, were used as estimates of movement-related BOLD activity. A standard block function, convolved with the hemodynamic response, was used for the observation experiment; the modeled function started with the presentation of the video clip and lasted 1 second. To avoid that baseline fMRI activity could reflect the 2-alternatives task, this was modeled with a 2 seconds-long block function and the estimated BOLD responses were discarded. The t-score maps from the tent functions (for the execution and imagery experiments) and from the block functions relative to the movie clip (for the observation experiment) were selected for data sharing.

#### Experimental set-up differences among research centers

All the data acquisitions were preformed according to an integrated set of protocols. For what concerns the SoftPro protocol, the different research centers shared the same list of actions. However, specific cases required some adaptation of the general framework. Differences with respect to the general setup are reported in this section.

- Experiments of Group D were carried out inside an electromagnetically isolated chamber. For this reason, participants were not able to execute task 22 (tennis smash) of the SoftPro protocol. This task was replaced with the following one: Reach and grasp a smartphone, unlock the screen, dial a number, and put it back to the initial position. See also [33] for additional details.

## Analyses and Technical Validation

### Kinematic data

Quality of kinematic data was tested through the evaluation of SNR.

#### ID *H*_1_

Data of these experiments were collected using the PhaseSpace motion capture system, a commercial device that tracks precise motion data with submillimeter resolution (the amount of static marker jitter is <0.5 mm, usually 0.1 mm). Ten stereo-cameras were placed around the participant so as to fully cover the scene (360°). The system was fully calibrated before the acquisition of each participant, following the standard procedure described by the manufacturer. Marker IDs are automatically associated by the proprietary software tool. For these data, we quantified the SNR by selecting the 3 seconds of rest before the execution of each task to estimate measurement noise, and a sample of 3 seconds of signal during the execution of the task itself (vectors of same length). We used for this analysis 1 marker placed on the hand dorsum, i.e., the worst-case scenario because of the reduced distance between markers. Then, from the *x, y, z* vectors of markers’ trajectories we calculated the norm and removed the mean. From signal and noise vectors, SNR was calculated through the Matlab snr routine. We randomly selected 20 trials from the dataset and quantified the SNR for each sample. We obtained a median value of 37.54 (interquartile range [IQR], 4.56). These data were used for kinematic reconstructions that were used for the principal component analysis (PCA) and functional PCA, which outcomes are discussed in [19] and [17], respectively. The reader can refer to those works for an example on how to pre-process and analyze the data. We also report a pseudocode (see Alg. 1) of the motion identification procedure used in [17] to calculate joint angular values from readings of the motion capture system. This should serve as an example of data analysis that can be tailored on different acquisition systems.

**Table:** 

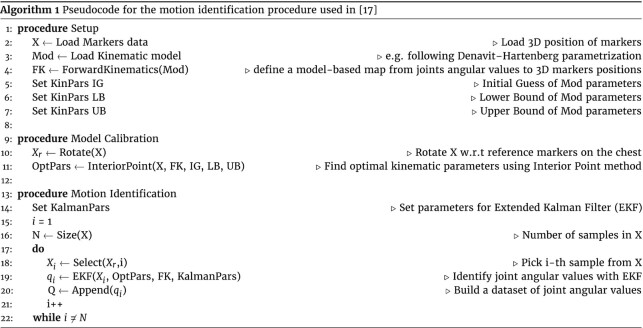

#### ID *H*_4_

Data of these experiments were collected through the Vicon motion capture system, a commercial device that ensures submillimeter errors in static conditions (see [34]). Twelve cameras were used to record the scene from multiple perspectives. Marker labeling and trajectory reconstruction were performed through the proprietary software Nexus v1.8.5. For these data, SNR was quantified following the same procedure of H_1_. From a random selection of 20 trials, we obtained a median value of 44.12 (IQR, 3.09).

#### ID *H*_6_

Data of these experiments were collected through an IMU-based sensor suit, a commercial device by Xsens technologies B.V., Enschede, The Netherlands. The producer declares an accuracy in angle estimation of 0.2° for roll/pitch and 0.5° for heading angles in static conditions. These values are increased to the value of 1° in dynamic conditions. The whole acquisition system was properly calibrated, following the manufacturer’s guidelines, before the acquisition of each participant. For these data, we quantified SNR following the same procedure used with the previous cases. SNR was evaluated on the norm of roll/pitch/yaw angles of the arm with regard to the chest (shoulder DoFs). Our analysis on a random selection of 20 trials reported a median value of 40.73 (IQR, 5.87).

#### ID *H*_7_

Data of these experiments were collected through a Vicon motion capture system, similar to the one used in H_4_. As previously stated, this system ensures submillimeter errors in static conditions (see [34]). Also in this case we quantified the SNR of data associated with the 3D position of markers placed on the hand dorsum. Our analysis on a random selection of 20 trials resulted in a median value of 45.0 (IQR, 6.48).

### EMG data

All the experiments that involved the recording of EMG data were performed by expert experimenters who followed the SENIAM guidelines for skin preparation and electrode placement [35]. This represents a gold standard in EMG signal recording and treatment, which guarantees the highest data quality. Before the placement of EMG sensors, the corresponding skin areas were cleaned with abrasive and conductive cleaning pastes (skin impedance was controlled <30 kΩ). Before each acquisition, the recorded data were carefully visually checked on-line by an expert experimenter, and sensor locations were adjusted if necessary. Part of these data were successfully used for the identification of task-dependent muscle synergies in [33] and for the validation of a human shoulder-arm musculoskeletal dynamic model in [36], to which the interested reader is referred for an example on how to pre-process and analyze the data. It is worth mentioning that in the literature EMG data typically undergo a number of pre-processing steps to increase the quality of the collected signal and make it usable for further analyses. Because in this publication we are releasing raw data, it is difficult to find references for quantitative SNR calculated on raw data. To evaluate the SNR on the raw data released with this publication, we first performed a high-pass filtering on each bipolar channel (fourth-order Butterworth filter, cut-off frequency of 10 Hz) to remove baseline shifts. Then, we calculated the SNR for each sample and for each bipolar channel. The estimation of the SNR is based on [37], and a Matlab implementation is also available [38]. This evaluation of the SNR defines the noise as an unidentifiable high-frequency component concentrated on the upper 20% of the frequency range (ensuring all frequencies are >500 Hz). The module of the noise is then estimated as the average of all the power densities in the upper 20% frequency range. Then, the SNR is estimated as the ratio between the sum of all the power densities and the noise. In the data released with this publication, the median value of the SNR is always >10^2^.

### EEG

EEG data presented were already successfully exploited in different works and from different perspectives [23–26]. As is well known, many different pre-processing pipelines have been presented in the literature to properly analyze EEG signals; they can vary according to the specific further analyses that are intended to be performed on the dataset. For this reason, in [23–26] different processing steps were applied to remove artifacts and prepare the data for further analyses. A detailed description of the processing steps that have been implemented is thereby provided.

### VPIT

The VPIT test is based on a CE marked haptic device, i.e., PHANTOM Omni, SensAble Technologies, Inc., USA, with a nominal position resolution >450 dpi (0.055 mm). Grasping forces are recorded through 3 single-axis force sensors (CentoNewton 40, EPFL, Switzerland). Each sensor can accurately record force values in the range of 0−40 N, with a resolution of 0.05 N. The linear relationship between forces applied and voltages produced by the force sensors has been verified [39]. To do this, the sensor was dynamically loaded and unloaded (up to 100 N/s) to 3 force levels (approximately 10, 20, and 30 N) against a commercial load cell (Mini 40, ATI Industrial Automation, USA) while the voltage output of the piezoresistive sensor was measured. Force data were low-pass filtered at 50 Hz and show good linearity characteristic (*V* = 0.0915*F* + 0.726; *R*^2^ = 0.9987, where *F* is the applied force and *V* the voltage measured by the force sensor).

### fMRI

Quality check of fMRI data was performed using MRIQC [40]. MRIQC is a software package, part of the bids-apps [41], that performs several processing steps to derive different parameters regarding image quality, such as SNR measures and motion estimates (e.g., FD) that are graphically reported as image quality metrics (IQMs) from each run in each participant. Here, we ran MRIQC on raw functional data, and plots with IQMs and mean images from single runs are included in the QC folder, which is organized in the same way as the folder containing data. Group analysis—i.e., averages and distributions of IQMs across participants—is also included. For further information on the quality check pipeline, please refer to http://mriqc.org.

## Discussion and Potential Implications

The aim of this article is to provide an exhaustive description of the experimental protocols and acquisition techniques that finally led to the release of the dataset U-Limb. This dataset has a value per se because it represents an extraordinary and unique source of information, with multiple sensory modalities that concur to shed light on different aspects underpinning the motor control of the human upper limb. We firmly believe that the release of this dataset, together with all the information needed to reproduce the experiments, can be a key component for fostering data reuse and benchmarking, and finally advancing the research in the field of motor control. The objective is to contribute to the establishment of a transdisciplinary community and to the definition of well-accepted guidelines for data collection. Of note, some of the data reported in this article have already been used and analyzed for different research purposes, and the scientific outcomes have affected or could positively affect various fields, as already mentioned in the introductory part of the article. In the following we report some examples of the applications of our data and discuss the transdisciplinary impact. First and foremost, neuroscientific research can benefit from the analysis of U-Limb data. Thanks to the adoption of integrated experimental protocols, the kinematic, muscular, and dynamic mechanisms, as well as the central and autonomous nervous system components related to motion execution, can be investigated, at different levels of the muscle-skeletal system (e.g., fMRI data focus on the hand; kinematic data focus on the whole upper limb chain). In [17] a functional PCA was applied to the kinematic data of healthy participants, labeled as H_1_, to identify the principal functional modes of human upper limb movements. To summarize, the idea was to decompose the temporal trajectories of upper limb joints in terms of a basis of functions. The results showed that a combination of a few functional principal components is sufficient to reconstruct a large part of the variability of joint evolutions over time, in activities of daily living. This observation has led to the definition of a planning problem for the generation of human-like movements in robot manipulators. Briefly, the human upper limb principal motion modes computed through functional analysis were embedded in the robot trajectory optimization, thus intrinsically ensuring robot human-likeness in free motions and for obstacle avoidance [42,43]. This point is of paramount importance in advanced human-robot interaction and assistive applications, to guarantee the safety of the human operator and the acceptability of the robotic technologies [44]. The kinematic data labeled as H_1_ were also analyzed in [19], to characterize the upper limb poses at each time frame, through a technique that was named “repeated principal component analysis." The outcomes demonstrated that the subspace identified by the first 3 principal components takes into account most of the motion variability, and these results were proven to be stable over time and consistent across participants. These findings could inform the definition of control laws for upper limb robotic devices, relying on a time-invariant low-dimensional approximation of upper limb kinematics, within the general framework of synergistic control [16]. For what concerns the kinematic data on post-stroke participants, it is worth reporting the results described in [21]. Briefly, the data labeled as P_1_ were analyzed to evaluate the variations of functional principal components applied to the reconstruction of joint angle trajectories. These variations were compared between 2 conditions, i.e., the affected and non-affected arm, to devise a dissimilarity index for achieving an accurate and quantitative assessment of upper limb motion impairment induced by stroke. This point is extremely important to overcome the limitations of current evaluation procedures, which are mostly based on ordinal scaling, operator-dependent, and subject to floor and ceiling effects, to pave the path for a more analytical assessment that could inform the rehabilitation procedures. On the same line, the kinematic and haptic interaction data labeled as P_4_ were used to devise quantitative metrics to evaluate the neurological sensorimotor impairment of upper limb kineto-dynamic behavior, in virtual peg-in-hole tasks [45]. It is worth highlighting here one of the characteristics that make the U-Limb dataset unique: i.e., the possibility to have data that cover different yet related aspects of human upper limb motor control, which allow it to be analyzed under different perspectives and points of view (for the aforementioned examples, a purely kinematic point of view for P_1_ and the kineto-dynamic coordination in virtual manipulation tasks for P_4_). Considering the EEG data labeled as H_2_, in [23–25] they were used to automatically discriminate transitive, intransitive, and tool-mediated imaginary actions (as described in the SoftPro protocol) using EEG dynamics, and relying on non-linear support vector machine and fuzzy entropy techniques. Interestingly, in [25] different combinations of EEG-derived spatial and frequency information were investigated to find the most accurate feature vector, and sex differences between accuracies achieved with male and female data were observed. These results could open the path to sex-based models for the development of optimized brain machine interfaces. To conclude, U-Limb can positively affect different research fields, which encompass neuroscience and motor control, clinical assessment and rehabilitation, and robotics and advanced human machine interfaces.

## Methods

### Experimental protocols

#### SoftPro protocol

“Activities of daily living" is a term commonly used in rehabilitation to indicate a set of everyday tasks. More recently, the use of this class of movements has also become central in robotics to evaluate the use of artificial systems in daily actions. The criteria for the selection of a comprehensive list of activities include (i) the specific hand-grasping configuration and (ii) the direction of motion for the whole upper limb.

In the attempt to exhaustively consider all the possible combinations of (i) and (ii), we identified 30 tasks, which were divided in 3 different classes: intransitive, transitive, and tool-mediated actions. Intransitive tasks collect movements without contact with external objects; transitive tasks are actions that involve an external object; and, finally, tool-mediated tasks are actions in which an object is used to interact with another object. This particular classification takes inspiration from the analysis presented in [46], which was proven to be reflected at the cortical level in imaging studies, e.g., [47], that show differences in cortical activation between actions belonging to the 3 different classes, with prefrontal and parietal regions of the left hemisphere tuned towards tool-mediated and transitive actions, whereas the right hemisphere shows a preference for meaningful, intransitive gestures. This organization has been confirmed by clinical observations as well: classic neurological studies show that, following cortical stroke, patients can develop class-specific deficits for tool-mediated actions [48,49], and deficits for transitive or intransitive gestures have been described as a result of greater involvement of the left or right hemisphere, respectively [50].

Within a specific class, the selected actions cover different hand-grasping configuration in order to span most of the postures of the main hand-grasping taxonomies [51,52]. A detailed list of actions is reported in Table 4. In each row of the table, the first element reports the task number, the second links to the grasp taxonomy [51], the third indicates the class of movement, and, finally, the fourth reports a brief description of the task. More details can be found in [19]. During the experiment, each task was repeated ≥3 times, resulting in a minimum number of 90 independent acquisitions for each participant. The temporal timeline for task execution was (i) 3 seconds of rest, (ii) task execution at a self-paced speed, (iii) 3 seconds of rest. Regarding UP (IDs {H_1_, H_2_, H_3_}) a custom C++ routine was used to associate the pressure of a keyboard key with (i) the start of 3D marker position acquisition and (ii) the placement of a temporal marker in the acquisition flow of EEG/ECG recordings. The same tool was used to interrupt the task acquisition on both sides. Absolute timing is also provided in the related dataset. An analogous procedure was used at MHH (IDs {H_4_, H_5_} and {P_2_, P_3_}), and at TUM (IDs {H_7_, H_8_, H_9_}), where an EtherCAT system with NI 9144 (National Instruments), controlled using the Simulink tool of Matlab, was used to send start/stop trigger signals to the acquisition systems.

**Table 4:** List of actions defining the SoftPro protocol

No.	No. [51]	Task class	Description
1		Int	Ok gesture (lifting hand from the table)
2		Int	Thumb down (lifting hand from the table)
3		Int	Exultation (extending the arm up in the air with closed fist)
4		Int	Hitchhiking (extending the arm along the frontal plane, laterally, parallel to the floor, with extended elbow, closed fist, extended thumb)
5		Int	Block out sun from own face (touching the face with the palm and covering the eyes)
6		Int	Greet (with open hand, moving wrist) (3 times)
7		Int	Military salute (with lifted elbow)
8		Int	Stop gesture (extending the arm along the sagittal plane, parallel to the floor, open palm)
9		Int	Pointing (with index finger) at something straight ahead (with outstretched arm)
10		Int	Silence gesture (bringing the index finger, with the remainder of the hand closed, to the lips)
11	2	Tr	Reach and grasp a small suitcase by the handle, lift it, and place it on the floor (close to own chair, along own sagittal plane)
12	3	Tr	Reach and grasp a glass, drink for 3 seconds, and replace it in the initial position
13	4	Tr	Reach and grasp a phone receiver, carry it to own ear and hold for 3 seconds, and replace it in the initial position
14	6	Tr	Reach and grasp a book (placed overhead on a shelf), put in on the table, and open it (from right side to left side)
15	8	Tr	Reach and grasp a small cup by the handle (2 fingers + thumb), drink for 3 seconds, and replace it in the initial position
16	11	Tr	Reach and grasp an apple, mimic biting, and replace it in the initial position
17	12,13	Tr	Reach and grasp a hat bby its top and place it on own head
18	12	Tr	Reach and grasp a cup by its top, lift it, and put it on the left side of the table
19	15	Tr	Receive a tray (straight ahead, with open hand) and put it in the middle of the table
20	16	Tr	Reach and grasp a key in a lock (vertical axis), extract it from the lock, and put it on the left side of the table
21	1	T-M	Reach and grasp a bottle, pour water into a glass, and replace the bottle in the initial position
22	2,3,4	T-M	Reach and grasp a tennis racket (placed along own frontal plane) and play a forehand (the participant is still seated)
23	5	T-M	Reach and grasp a toothbrush, brush teeth (horizontal axis, 1 time left-right), and put it inside a holder (on the right side of the table)
24	6	T-M	Reach and grasp a laptop, open it (without changing its position) (4 fingers + thumb)
25	7,8,9	T-M	Reach and grasp a pen (placed on the right side of the table) and draw a vertical line on the table (from the top to the bottom)
26	7	T-M	Reach and grasp a pencil (placed along own frontal plane) (3 fingers + thumb) and put it inside a square pencil holder (placed on the left side of the table)
27	9	T-M	Reach and grasp a tea bag in a cup (1 finger + thumb), remove it from the cup, and place it on the table on the right side of the table
28	10	T-M	Reach and grasp a doorknob, turn it clockwise and counterclockwise, and open the door
29	13	T-M	Reach and grasp a tennis ball (with fingertips) and place it in a basket on the floor (right)
30	14	T-M	Reach and grasp a cap (2 fingers + thumb) of a bottle (held by left hand), unscrew it, and place it overhead on a shelf

Int: intransitive; Tr: transitive; T-M: tool-mediated.

**Table 5:** List of 29 muscles recorded during the experiments at TUM (ID H_8_)

Electrode No.	Muscle
1	M. trapezius Pars descendens (TRPc)
2	M. trapezius Pars transversa (TRPt)
3	M. trapezius Pars ascendens (TRPa)
4	M. deltoideus Pars clavicularis (DLTc)
5	M. deltoideus Pars acromialis (DLTa)
6	M. deltoideus Pars spinalis (DLTs)
7	M. latissimus dorsi (LTDt)
8	M. pectoralis major Pars clavicularis (PMJc)
9	M. pectoralis major Pars sternocostalis (PMJs)
10	M. pectoralis major Pars abdominalis (PMJr)
11	M. biceps brachii Caput longum (BICl)
12	M. biceps brachii Caput breve (BICs)
13	M. triceps brachii Caput longum (TRClg)
14	M. triceps brachii Caput laterale (TRClt)
15	M. pronator teres (PRNT)
16	M. flexor carpi radialis et (if present) M. palmaris longus (FCR)
17	M. flexor carpi ulnaris (FCU)
18	M. flexor digitorum superficialis (FDS)
19	M. flexor pollicis longus (FPL)
20	M. extensor digitorum (EDT)
21	M. extensor digiti minimi (EDM)
22	M. extensor carpi ulnaris (ECU)
23	M. abductor pollicis longus et M. extensor pollicis brevis (APL&EPB)
24	M. brachioradialis (BRD)
25	M. extensor carpi radialis (ECR)
26	M. abductor digit minimi (ADM)
27	M. flexor pollicis brevis (FPB)
28	M. abductor pollicis brevis (APB)
29	M. interosseus dorsalis I (DI1)

**Table 6:** List of objects used for the fMRI experiments

Bucket	Calculator	Chalk	Cherry
Dinner plate	Espresso cup	Fishing rod	Flying disc
Hairdryer	Hammer	Telephone handset	Jar lid
Light bulb	PC mouse	Pen rope	Ice cube
Tennis racket	Toothpick	Wrench	

#### VPIT protocol

**Figure 6:**
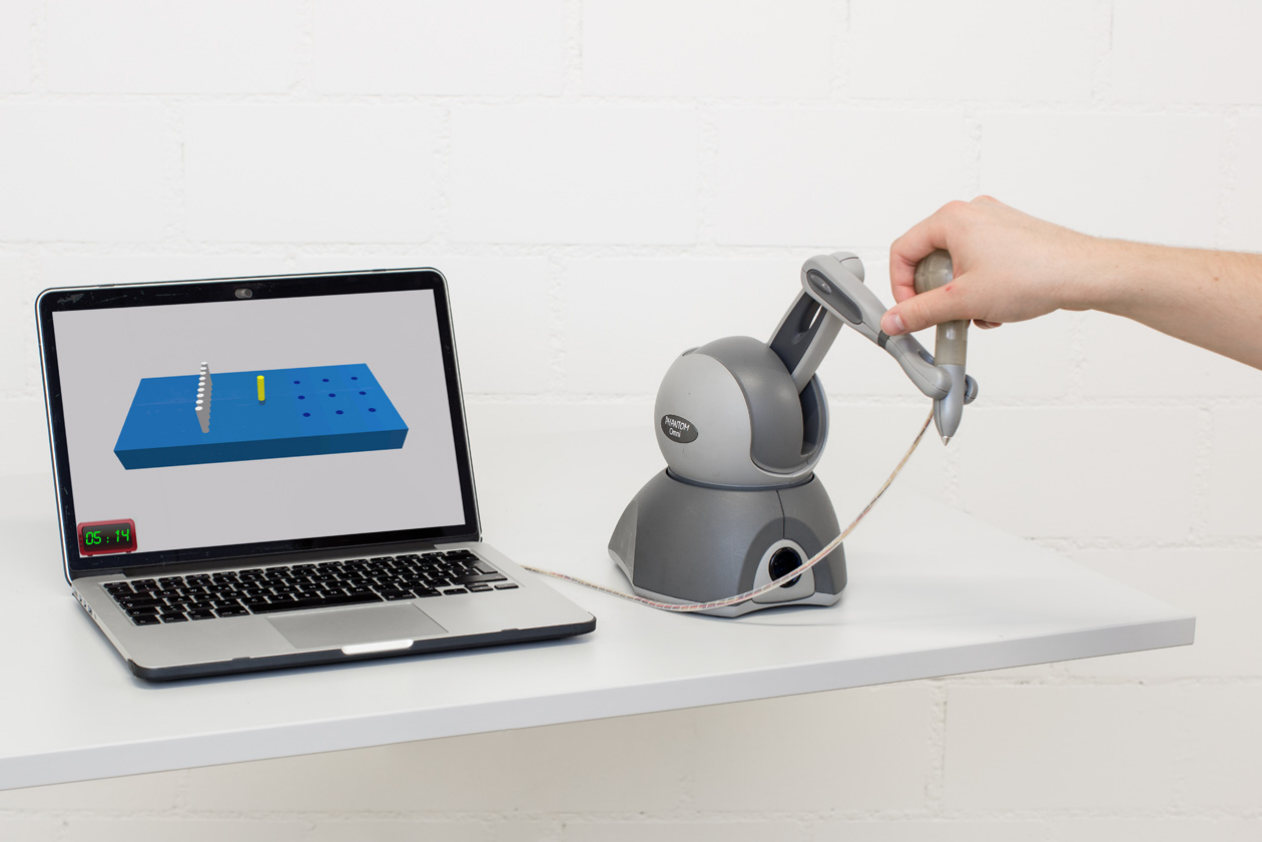
The Virtual Peg Insertion Test (VPIT) is a technology-aided assessment platform consisting of a haptic end-effector, a grasping force sensing handle, and a virtual reality environment. It allows recording of kinematic and kinetic data about sensorimotor impairments in arm and hand during a functional task.

The VPIT is performed using a commercial haptic end-effector (PHANTOM Omni, 3D Systems, Rock Hill, South Carolina, USA), a custom-made handle with force sensors (CentoNewton40, EPFL, Switzerland), and a virtual reality environment rendered on personal computer (Fig. 6) [39, 53–55]. The VPIT requires the insertion of 9 virtual pegs into 9 virtual holes through the coordination of arm and hand movements controlling the end-effector as well as the grasping forces applied to the instrumented handle attached at the end-effector. In more detail, a virtual cursor needs to be first spatially aligned with the virtual peg. Subsequently, a peg can be picked up and transported towards a hole by applying a grasping force of ≥2 N. The peg can be released in the hole by reducing the grasping force below the threshold. The virtual pegboard is thereby physically rendered through the haptic device to ease the perception of the 3D virtual reality environment.

The starting position of the participants was defined through an elbow flexion angle of ≈90°, a shoulder abduction angle of ≈45°, and a shoulder flexion angle of ≈10°. The protocol consists of an initial familiarization period, during which participants were instructed to perform the task as fast and precise as possible, followed by 5 repetitions of the task (i.e., inserting all 9 pegs 5 times). More details about the set-up and the procedure can be found in previous work [28, 39].

#### fMRI protocol

Designs for motor execution and imagery experiments were based on a previous work [56] and relied on a delayed grasping task after a visual presentation of the target objects. More specifically, in each trial, a picture of the target object was visually presented for 2 seconds, then, after a 4-second pause, an auditory cue prompted the actual task: participants had to preshape the hand as if they were grasping the target object to use it (for the execution group) or imagine a preshaping movement without moving their hand (for the imagery group). A 10-second interval separated 2 subsequent trials. Twenty different target objects were used for this study (see Table 6 for a list), and, in each experiment, movements were repeated 5 times, for a total number of 100 trials, organized in 5 fMRI runs, each lasting 5 minutes 44 seconds, including 12 seconds of rest at the beginning and at the end of each run to achieve a measure of baseline fMRI activity. The experimental paradigm for execution and imagery experiments was coded using Presentation (Neurobehavioral System, Berkeley, CA), and presented with an MR-compatible monitor at the resolution of 1,200 × 800 pixels, and a mirror mounted on the MR coil. During the observation experiment, participants watched short videos of preshaping movements towards an object from the same set adopted in the other experiments. In each trial, the video was followed by a task that implied a judgment on the target of the preshaping gesture. To create videos, we used vectors of joint angles (according to a 24 DoFs model) corresponding to the common starting posture and to the 20 final object-specific postures, recorded in a previous study [56]. Intermediate hand configurations (i.e., posture vectors) between the initial and final postures were obtained from linear interpolation between the values of each kinematic joint angle in the initial and final hand postural configurations. The resulting 30 vectors of joint angles were plotted as 3D renderings, using Mathematica 8.0 (Wolfram Research Inc, Champaign, IL, USA), saved as png images (size: 800 × 600 pixels), and converted to 1 second-long videos at a frame rate of 60 Hz. Five sets of 20 videos were created, showing the hand rendering as seen from 5 different viewpoints, obtained by changing the values of azimuth and elevation. During the fMRI experiment, participants performed 5 runs, each comprising 20 trials. During each trial, the video was presented (1 second), followed by a black fixation cross at the center of the screen (7 seconds). Then, the judgment task (2-alternatives forced choice) was presented, and participants were shown the black/white pictures of 2 objects (size: 250 × 250 pixels)—the target of the preshaping gesture previously shown and a randomly chosen alternative—and asked to press the left or right key on an MR-compatible keyboard to select the actual target of the preshaping movement. After the task, the same black fixation cross was shown for 6 seconds. Each run comprised the presentation of the full set of 20 videos (20 objects), always from the same viewpoint; the 5 different viewpoints were presented in separate runs. Each run started and ended with 10 seconds of rest and lasted in total 5 minutes 40 seconds. The experimental paradigm was delivered with an MR-compatible monitor at the resolution of 1,200 × 800 pixels, and a mirror mounted on the MR coil, using the e-Prime 2 software package (Psychology Software Tools, Pittsburgh, PA, USA). Owing to hardware failure, behavioral responses from 2 participants could not be recorded. For all experiments, participants performed a familiarization run, outside the MR scanner, to ensure that they correctly understood the procedures.

### Data Records

Data records published with this article, together with the dataset summary and ReadMe, are available through the Harvard Dataverse repository and can be downloaded []. The overall size is 36.2 GB. Data are organized in 6 folders, 1 for each research center. Within every folder, data are organized per recording modality (e.g., kinematic data, EMG, and EEG). Data provided from each institution have been separately compressed in .rar format and uploaded on the repository, in such a way to enable the download of a single block of data. For blocks heavier than 2.5 GB, we divided the file in multiple linked parts. In these cases, to properly unpack the data the reader is required to extract the file named XXX.part1, which in turn will automatically recall the subsequent parts. Each folder contains a ReadMe file that details the folder content. In the following, we provide more detailed information for each folder

#### Folder UP

In this folder, data are organized per recording modality, i.e., EEG-ECG and KIN. Each folder contains in turn 39 folders named “SXX,” where XX is the participant ID. The folder Data_KIN contains the kinematic acquisition. Files are named as “SXX_Y_Z,” where Y is the task number and Z is the repetition number (e.g., S4_23_1). Data are collected with a sampling rate of 100 Hz. Each acquisition is provided in the dedicated mat file.

An identical naming convention has been used for the corresponding (synchronized) data of EEG-ECG signals, contained in the “Data EEG - ECG” folder. This folder contains the MFF files with the EEG and ECG data (in millivolts). Data were gathered through an EGI 128-channel system (sampling rate of 500 Hz). Each acquisition is complemented by a number of markers that identify the beginning of each repetition of a single task.

Note that, for these experiments, 3 repetitions of the same task are provided. There are some cases in which the Z value (repetition number) is >3. This can be associated with cases in which we noticed (i) errors in the task execution or (ii) evident problems in the acquisition (either in kinematic data or in EEG data). In these cases, we performed additional repetitions to guarantee the minimum number of 3 samples of the same task. Acquisitions containing evident errors have been discarded from the dataset.

In addition, 2 additional folders are included, namely, “read_EEG” and “read_plot_KIN,“ in which we provide sample codes to access and plot the dataset. Further information about the data and the code is included in the ReadMe file.

#### Folder MHH

In this folder, data are organized per recording modality, i.e., EMG data and kinematic data. These 2 subfolders are divided in healthy and post-stroke participants.

Trials are named with 3 numbers (e.g., 10_8_3), where the first number (in the example 10) indicates the participant ID, the second (8 in the example) indicates the task number, and the third is the trial number. Post-stroke participants are named the same way but with the addition of an “S” at the beginning of the name.

EMG data are organized to have the rows corresponding to the time frames (sampling rate 2,000 Hz) and the columns associated with the 12 measured muscles. The kinematic data files contain the position data of thorax, upper arm, and forearm markers. The table is divided in 63 columns, with 3 columns, corresponding to the *x, y,z* position, for each of the 21 markers; the rows, starting from the third one, report the recorded marker position for each time frame (sampling rate of 200 Hz). The first 2 rows contain, respectively, the marker names and the measure unit (in millimeters).

The file read_emgfiles.m is a sample Matlab code to plot the EMG data. Additional details are provided in the ReadMe file. Participant-specific information is provided in 1 additional file, named “Patients_details_MHH_extended.doc”. There we reported the following characteristics: ID, age, sex, tested limb, impaired limb, dominant limb, time since stroke, FMA-UE, and MM score.

#### Folder UZH

In this folder, data are organized for each participant who took part in the experiment, i.e., healthy and impaired participants. Kinematic parameters are stored in a software-specific XML file format (.mvnx) that enables the import to different software tools, such as Matlab and Microsoft Excel. Each mvnx file represents 1 trial execution and is named according to the participant ID (e.g., P02), task number (T01–T30), tested upper limb (R/L), and repetition (1–3). Sample Matlab codes are provided, showing how to access and plot data. More information regarding the file structure and how to plot data is provided in the ReadMe file. Participant-specific information is provided in the additional file “ParticipantCharacteristics.xlsx”. There we reported the following information: ID, age, sex, impaired limb, dominant limb, time since stroke, FMA-UE. Note that the 20 post-stroke participants enrolled in this dataset (Group α) are a subset of the 27 who performed the VPIT protocol (Group γ). Therefore, further information on participants of this folder may also be included in the additional files included in Folder ETHZ (the ID of participants is consistent in the 2 datasets).

#### Folder TUM

In this folder, data are organized per participant. Each subfolder is divided per recording modality, i.e., EEG, EMG, and kinematic Data (MoCap folder). Matlab files are provided to access and plot data (i.e., plot_KIN.m, plot_EMG.m, and plot_EEG.m).

#### Folder ETHZ

In this folder, the provided VPIT_Data_v3.mat file contains processed and unprocessed VPIT data collected from 27 post-stroke individuals. Data are all contained in the VPIT_Data_v3.mat file, in which each row corresponds to data from 1 specific trial. The file header.xlsx contains detailed meta-information regarding the content of each column in the VPIT_Data_v3.mat. Additional information about the data, processing, and procedures is provided in the ReadMe file. Participant-specific information is provided in 2 additional files, named “patient-information.png” and “patient-information-2.png”. There we reported the following characteristics: ID, age, sex, tested limb, impaired limb, dominant limb, time since stroke, FMA-UE, ARAT, NHPT, BBT, MAS, EmNSA, and MOCA.

#### Folder IMT

In this folder, data are organized according to the Brain Imaging Data Structure (BIDS) standard [57]. Single-participant t-score maps from functional data are included in the directory for processed data (i.e., derivatives). For the execution and imagery experiments, t-scores for the fifth, sixth, and seventh tent functions (i.e., with peak at 2, 4, 6 seconds after movement onset) are selected. Each stimulus is modeled using its 5 repetitions. The .nii.gz file contains the average of the 3 selected t-score maps. For the observation experiment, t-scores for the block functions, covering the stimulus period, are selected. Each stimulus is modeled using its 5 repetitions. The 2AFC task responses, though modeled, were discarded. The .nii.gz file contains the 20 t-score maps, 1 for each stimulus. Structural data are shared as anonymized, raw images in the directories for single-participant raw files. Participants Nos. 1–9 performed the execution experiment, whereas participants Nos. 10–18 performed the imagery experiment, and participants Nos. 19–27 performed the observation experiment. Detailed information about the data analysis procedure and participants is given in the README, dataset_description.json, and participants’ .tsv files, respectively.

## Availability of Source Code and Requirements

For each set of data released with this manuscript, we included dedicated Matlab codes to access and, when possible, plot data. Please refer to the ReadMe of each folder and to the specific files for a detailed description. All the codes were tested with Matlab version R2019b (Mathworks Inc., Natick, MA, USA).

## Data Availability

All the data associated with this manuscript are available in the Harvard Dataverse repository [58].

## Abbreviations

BIDS: Brain Imaging Data Structure; DoF: degree of freedom; ECG: electrocardiography; EEG: electro-encephalography; EMG: electromyography; FD: framewise displacement; fMRI: functional magnetic resonance imaging; IQM: image quality metric; HCGSN: HydroCel Geodesic Sensor Net; IMU: Inertial Measurement Unit; IQR: interquartile range; PPCA: principal component analysis; IB: polygraph input box; sEMG: surface electromyography; SENIAM: surface EMG for non-invasive assessment of muscles; SNR: signal to noise ratio; VPIT: Virtual Peg Insertion Test.

## Ethical Approval

All the experiments conducted to build this collection of data were approved by local ethical committees. Table 1 contains additional information on the approving institution and protocol number.

## Consent for Publication

All participants gave their written informed consent for publication. All the experiments were performed in accordance with the Declaration of Helsinki, and in observation of the “Guideline for good clinical practice E6(R1)International Council for Harmonization of Technical Requirements for Pharmaceuticals for Human Use (ICH).”

## Competing Interests

The authors declare that they have no competing interests.

## Funding

This project has received funding from the European Union’s Horizon 2020 research and innovation programme under grant agreement No. 688857 (SoftPro).

## Authors’ Contributions

All the authors contributed to the design of the experimental protocol and to the development of the different set-ups. G.A., F.B., V.C., M.B., and G.V. performed the experiments at UP. R.G., C.K., and O.L. performed the experiments at ETHZ. T.H. and J.K. (TUM) performed the experiments in Leibniz Universität Hannover, Germany. G.H., A.L., and E.R. performed the experiment at IMT. J.H. and A.S. performed the experiments at UZH. E.J. and A.O. performed the experiments at MHH. G.A. and M.B. prepared the first version of the manuscript. All the authors participated in the preparation and revision of the final manuscript.

## Additional Information

Given the international effort provided to prepare this manuscript, and the firm belief that sharing and reusing human data is of paramount importance for the research community in multiple fields, such as neuroscience, motion control, robotics, rehabilitation, and clinical practice, the authors are willing to continue nourishing U-Limb with additional data, when available. Under these regards, other research groups are warmly invited to contribute to U-Limb with data on the human control of limbs, with specific focus on the upper extremities in both healthy and disease conditions. The latter can refer to any disease condition that induces a sensorimotor impairment in the upper limb (not only stroke but also traumatic brain injury, spinal cord injury, injuries to motoneurons, multiple sclerosis, cerebral palsy, Guillain-Barré syndrome, essential tremor, Parkinson disease, autosomal recessive spastic ataxia of Charlevoix–Saguenay, and so forth), which may be investigated through different acquisitions modalities, such as kinematics, EMG, EEG, fMRI, and others. To participate, please contact the Corresponding Author. New data will be associated either with a completely new submission or with an “Update” on this Data Note, for submission to *GigaScience*’s sister journal, *GigaByte*.

## Supplementary Material

giab043_GIGA-D-21-00005_Original_Submission

giab043_GIGA-D-21-00005_Revision_1

giab043_GIGA-D-21-00005_Revision_2

giab043_Response_to_Reviewer_Comments_Original_Submission

giab043_Response_to_Reviewer_Comments_Revision_1

giab043_Reviewer_1_Report_Original_Submission NÃ©stor J. Jarque-Bou -- 1/19/2021 Reviewed

giab043_Reviewer_1_Report_Revision_1 NÃ©stor J. Jarque-Bou -- 5/2/2021 Reviewed

giab043_Reviewer_2_Report_Original_Submission Michael Pereira -- 2/3/2021 Reviewed

giab043_Reviewer_2_Report_Revision_1 Michael Pereira -- 5/3/2021 Reviewed

## References

[1] Huang Y , Bianchi M, Liarokapis M, et al. Recent data sets on object manipulation: a survey. Big Data. 2016;4(4):197–216. 27992265 10.1089/big.2016.0042

[2] Jarque-Bou NJ , Scano A, Atzori M, et al. Kinematic synergies of hand grasps: a comprehensive study on a large publicly available dataset. J Neuroeng Rehab. 2019;16(1):63. 10.1186/s12984-019-0536-6 PMC6540541 31138257

[3] Santuz A , Ekizos A, Janshen L, et al. Modular control of human movement during running: an open access data set. Front Physiol. 2018;9:1509. 30420812 10.3389/fphys.2018.01509 PMC6216155

[4] Scano A , Chiavenna A, Molinari Tosatti L, et al. Muscle synergy analysis of a hand-grasp dataset: a limited subset of motor modules may underlie a large variety of grasps. Front Neurorobot. 2018;12:57. 30319387 10.3389/fnbot.2018.00057 PMC6167452

[5] Saudabayev A , Rysbek Z, Khassenova R, et al. Human grasping database for activities of daily living with depth, color and kinematic data streams. Sci Data. 2018;5:180101. 29809171 10.1038/sdata.2018.101 PMC5972673

[6] Schreiber C , Moissenet F. A multimodal dataset of human gait at different walking speeds established on injury-free adult participants. Sci Data. 2019;6(1):111. 31270327 10.1038/s41597-019-0124-4 PMC6610108

[7] Matran-Fernandez A , Martínez IJR, Poli R, et al. SEEDS, simultaneous recordings of high-density EMG and finger joint angles during multiple hand movements. Sci Data. 2019;6(1):186. 31570723 10.1038/s41597-019-0200-9 PMC6768861

[8] Jarque-Bou NJ , Atzori M, Müller H. A large calibrated database of hand movements and grasps kinematics. Sci Data. 2020;7(1):12. 31919366 10.1038/s41597-019-0349-2 PMC6952409

[9] Roda-Sales A , Vergara M, Sancho-Bru JL, et al. Human hand kinematic data during feeding and cooking tasks. Sci Data. 2019;6(1):167. 31488844 10.1038/s41597-019-0175-6 PMC6754415

[10] Jarque-Bou NJ , Vergara M, Sancho-Bru JL, et al. A calibrated database of kinematics and EMG of the forearm and hand during activities of daily living. Sci Data. 2019;6(1):270. 31712685 10.1038/s41597-019-0285-1 PMC6848200

[11] Atzori M , Gijsberts A, Castellini C, et al. Electromyography data for non-invasive naturally-controlled robotic hand prostheses. Sci Data. 2014;1(1):140053. 25977804 10.1038/sdata.2014.53 PMC4421935

[12] Mandery C , Terlemez Ö, Do M, et al. The KIT whole-body human motion database. In: 2015 International Conference on Advanced Robotics (ICAR). IEEE; 2015:329–36.

[13] Atzori M , Müller H. The Ninapro database: a resource for sEMG naturally controlled robotic hand prosthetics. In: 2015 37th Annual International Conference of the IEEE Engineering in Medicine and Biology Society (EMBC). IEEE; 2015:7151–4. 10.1109/EMBC.2015.7320041 26737941

[14] Atzori M , Gijsberts A, Heynen S, et al. Building the Ninapro database: a resource for the biorobotics community. In: 2012 4th IEEE RAS & EMBS International Conference on Biomedical Robotics and Biomechatronics (BioRob). IEEE; 2012:1258–65.

[15] Dolatabadi E , Zhi YX, Ye B, et al. The Toronto rehab stroke pose dataset to detect compensation during stroke rehabilitation therapy. In: Proceedings of the 11th EAI International Conference on Pervasive Computing Technologies for Healthcare. New York, NY: Association for Computing Machinery; 2017:375–81.

[16] Santello M , Bianchi M, Gabiccini M, et al. Hand synergies: integration of robotics and neuroscience for understanding the control of biological and artificial hands. Phys Life Rev. 2016;17:1–23. 26923030 10.1016/j.plrev.2016.02.001 PMC5839666

[17] Averta G , Della Santina C, Battaglia E, et al. Unvealing the principal modes of human upper limb movements through functional analysis. Front Robot AI. 2017;4:37.

[18] Averta G , Angelini F, Bicchi A, et al. On the role of postural synergies for grasp force generation and upper limb motion control. In: International Conference on Neurorehabilitation. Springer; 2018:344–8.

[19] Averta G , Valenza G, Catrambone V, et al. On the time-invariance properties of upper limb synergies. IEEE Trans Neural Syst Rehabil Eng. 2019;27(7):1397–406. 31135365 10.1109/TNSRE.2019.2918311

[20] MVN User Manual. https://www.xsens.com/hubfs/Downloads/usermanual/MVN_User_Manual.pdf.

[21] Schwarz A , Averta G, Veerbeek JM, et al. A functional analysis-based approach to quantify upper limb impairment level in chronic stroke patients: a pilot study. Annu Int Conf IEEE Eng Med Biol Soc. 2019;2019:4198–204. 31946795 10.1109/EMBC.2019.8857732

[22] Hermens HJ , Freriks B, Disselhorst-Klug C, et al. Development of recommendations for SEMG sensors and sensor placement procedures. J Electromyogr Kinesiol. 2000;10(5):361–74. 11018445 10.1016/s1050-6411(00)00027-4

[23] Catrambone V , Greco A, Averta G, et al. EEG processing to discriminate transitive-intransitive motor imagery tasks: preliminary evidences using support vector machines. In: 2018 40th Annual International Conference of the IEEE Engineering in Medicine and Biology Society (EMBC). IEEE; 2018:231–4. 10.1109/EMBC.2018.8512239 30440380

[24] Catrambone V , Greco A, Averta G, et al. EEG complexity maps to characterise brain dynamics during upper limb motor imagery. In: 2018 40th Annual International Conference of the IEEE Engineering in Medicine and Biology Society (EMBC). IEEE; 2018:3060–3. 10.1109/EMBC.2018.8512912 30441040

[25] Catrambone V , Greco A, Averta G, et al. Predicting object-mediated gestures from brain activity: an EEG study on gender differences. IEEE Trans Neural Syst Rehabil Eng. 2019;27(3):411–8. 30762562 10.1109/TNSRE.2019.2898469

[26] Catrambone V , Averta G, Bianchi M, et al. Toward brain-heart computer interfaces: a study on the classification of upper limb movements using multisystem directional estimates. J Neural Eng. 2021,doi:10.1088/1741-2552/abe7b9. 33601354

[27] Klem GH , Lüders HO, Jasper HH, et al. The ten twenty electrode system: International Federation of Societies for Electroencephalography and Clinical Neurophysiology. Am J EEG Technol. 1961;1(1):13–9. 10590970

[28] Kanzler CM , Rinderknecht MD, Schwarz A, et al. A data-driven framework for selecting and validating digital health metrics: use-case in neurological sensorimotor impairments. NPJ Dig Med. 2020;3:80. 10.1038/s41746-020-0286-7 PMC7260375 32529042

[29] Bischoff-Grethe A , Ozyurt IB, Busa E, et al. A technique for the deidentification of structural brain MR images. Hum Brain Mapp. 2007;28(9):892–903. 17295313 10.1002/hbm.20312 PMC2408762

[30] Cox RW . AFNI: software for analysis and visualization of functional magnetic resonance neuroimages. Comput Biomed Res. 1996;29(3):162–73. 8812068 10.1006/cbmr.1996.0014

[31] Jenkinson M , Beckmann CF, Behrens TE, et al. Fsl. Neuroimage. 2012;62(2):782. 21979382 10.1016/j.neuroimage.2011.09.015

[32] Power JD , Barnes KA, Snyder AZ, et al. Spurious but systematic correlations in functional connectivity MRI networks arise from subject motion. Neuroimage. 2012;59(3):2142–54. 22019881 10.1016/j.neuroimage.2011.10.018 PMC3254728

[33] Hu T , Kuehn J, Haddadin S. Identification of human shoulder-arm kinematic and muscular synergies during daily-life manipulation tasks. In: 2018 7th IEEE International Conference on Biomedical Robotics and Biomechatronics (Biorob). IEEE; 2018:1011–8.

[34] Merriaux P , Dupuis Y, Boutteau R, et al. A study of vicon system positioning performance. Sensors. 2017;17(7):1591. 10.3390/s17071591 PMC5551098 28686213

[35] Stegeman D , Hermens H. Standards for surface electromyography: The European project Surface EMG for non-invasive assessment of muscles (SENIAM). Enschede: Roessingh Research and Development.2007; 108–12.

[36] Hu T , Kühn J, Haddadin S. Forward and inverse dynamics modeling of human shoulder-arm musculoskeletal system with scapulothoracic constraint. Comput Methods Biomech Biomed Eng. 2020;23(11):785–803. 10.1080/10255842.2020.1764945 32552013

[37] Sinderby C , Lindstrom L, Grassino A. Automatic assessment of electromyogram quality. J Appl Physiol. 1995;79(5):1803–15. 8594044 10.1152/jappl.1995.79.5.1803

[38] MatLab Library. http://www.sce.carleton.ca/faculty/chan/matlab/matlab_library.htm. Accessed: June 03, 2021.

[39] Fluet MC , Lambercy O, Gassert R. Upper limb assessment using a virtual peg insertion test, IEEE Int Conf Rehabil Robot. 2011;2011:5975348. 22275552 10.1109/ICORR.2011.5975348

[40] Esteban O , Birman D, Schaer M, et al. MRIQC: advancing the automatic prediction of image quality in MRI from unseen sites. PLoS One. 2017;12(9):e0184661. 28945803 10.1371/journal.pone.0184661 PMC5612458

[41] Gorgolewski KJ , Alfaro-Almagro F, Auer T, et al. BIDS apps: improving ease of use, accessibility, and reproducibility of neuroimaging data analysis methods. PLoS Comput Biol. 2017; 13(3):e1005209. 28278228 10.1371/journal.pcbi.1005209 PMC5363996

[42] Averta G , Della Santina C, Valenza G, et al. Exploiting upper-limb functional principal components for human-like motion generation of anthropomorphic robots. J Neuroeng Rehab. 2020;17(1):63. 10.1186/s12984-020-00680-8 PMC7218840 32404174

[43] Averta G , Caporale D, Della Santina C, et al. A technical framework for human-like motion generation with autonomous anthropomorphic redundant manipulators. In: 2020 IEEE International Conference on Robotics and Automation (ICRA). IEEE; 2020:3853–9.

[44] Fink J . Anthropomorphism and human likeness in the design of robots and human-robot interaction. In: International Conference on Social Robotics. Springer; 2012:199–208.

[45] Kanzler CM , Rinderknecht MD, Schwarz A, et al. A data-driven framework for selecting and validating digital health metrics: use-case in neurological sensorimotor impairments. NPJ Digit Med. 2020;3, doi:10.1038/s41746-020-0286-7. PMC7260375 32529042

[46] Cubelli R , Marchetti C, Boscolo G, et al. Cognition in action: testing a model of limb apraxia. Brain Cogn. 2000; 44(2): 144–65. 11041987 10.1006/brcg.2000.1226

[47] Handjaras G , Bernardi G, Benuzzi F, et al. A topographical organization for action representation in the human brain. Hum Brain Mapp. 2015;36(10):3832–44. 26138610 10.1002/hbm.22881 PMC6869699

[48] De Renzi E , Lucchelli F. Ideational apraxia. Brain. 1988;111(5):1173–85. 3179688 10.1093/brain/111.5.1173

[49] Ochipa C , Rothi LG, Heilman KM. Ideational apraxia: a deficit in tool selection and use. Ann Neurol. 1989;25(2):190–3. 2465733 10.1002/ana.410250214

[50] Stamenova V , Roy EA, Black SE. Associations and dissociations of transitive and intransitive gestures in left and right hemisphere stroke patients. Brain Cogn. 2010;72(3):483–90. 20167414 10.1016/j.bandc.2010.01.004

[51] Cutkosky MR et al. On grasp choice, grasp models, and the design of hands for manufacturing tasks. IEEE Trans Robot Autom. 1989;5(3):269–79.

[52] Feix T , Romero J, Schmiedmayer HB, et al. The grasp taxonomy of human grasp types. IEEE Trans Hum Mach Syst. 2015;46(1):66–77.

[53] Kanzler CM , Schwarz A, Held JP, et al. Technology-aided assessment of functionally relevant sensorimotor impairments in arm and hand of post-stroke individuals. J Neuroeng Rehabil. 2020;17(1):128. 32977810 10.1186/s12984-020-00748-5 PMC7517659

[54] Kanzler CM , Gomez SM, Rinderknecht MD, et al. Influence of arm weight support on a robotic assessment of upper limb function. In: 2018 7th IEEE International Conference on Biomedical Robotics and Biomechatronics (Bioob). IEEE; 2018, doi:10.1109/BIOROB.2018.8487682.

[55] Kanzler CM , Catalano MG, Piazza C, et al. An objective functional evaluation of myoelectrically-controlled hand prostheses: a pilot study using the Virtual Peg Insertion Test. 2019 IEEE 16th International Conference on Rehabilitation Robotics (ICORR). IEEE; 2019:392–7. 10.1109/ICORR.2019.8779550 31374661

[56] Leo A , Handjaras G, Bianchi M, et al. A synergy-based hand control is encoded in human motor cortical areas. Elife. 2016;5:e13420. 26880543 10.7554/eLife.13420 PMC4786436

[57] Gorgolewski KJ , Auer T, Calhoun VD, et al. The brain imaging data structure, a format for organizing and describing outputs of neuroimaging experiments. Sci Data. 2016;3(1):160044. 27326542 10.1038/sdata.2016.44 PMC4978148

[58] Averta G , Barontini F, Catrambone V, et al. U-Limb. Harvard Dataverse. 2020. 10.7910/DVN/FU3QZ9.

