# Supplementary material for: U-Limb: A multi-modal, multi-center database on arm motion control in healthy and post-stroke conditions
Source: Gigascience. 2021 Jun 18;10(6):giab043. doi: 10.1093/gigascience/giab043 (PMC8212873; doi:10.1093/gigascience/giab043)

GigaScience

U-Limb: A multi-modal, multi-center database on arm motion control in healthy and post-stroke conditions.

--Manuscript Draft--

Manuscript Number:	GIGA-D-21-00005R2
Full Title:	U-Limb: A multi-modal, multi-center database on arm motion control in healthy and post-stroke conditions.
Article Type:	Data Note
Funding Information:	Horizon 2020 (688857) — Not applicable
Abstract:	Shading light on the neuroscientific mechanisms of human upper limb motor control, both in healthy and pathological conditions (e.g. after a stroke), can help to devise effective tools for a quantitative evaluation of the impaired conditions, and to properly inform the rehabilitative process. Furthermore, the design and control of mechatronic devices can also benefit from such neuroscientific outcomes, with important implications for assistive and rehabilitation robotics and advanced human-machine interaction. To reach these goals, we believe that an exhaustive data collection on human behavior is a mandatory step. For this reason, we release U-Limb , a large, multi-modal, multi-center data collection on human upper-limb movements, with the aim of fostering trans-disciplinary cross-fertilization. This collection of signals consists of data from 91 able bodied and 65 post-stroke subjects and is organized at three levels: (i) upper limb daily living activities, during which kinematic and physiological signals (electro-myography, electro-encephalography and electro-cardiography) were recorded; (ii) force-kinematic behavior during precise manipulation tasks with a haptic device; (iii) brain activity during hand control using functional magnetic resonance imaging.
Corresponding Author:	Giuseppe Averta<br>University of Pisa: Universita degli Studi di Pisa<br>Pisa, ITALY
Corresponding Author Secondary Information:	
Corresponding Author's Institution:	University of Pisa: Universita degli Studi di Pisa
Corresponding Author's Secondary Institution:	
First Author:	Giuseppe Averta
First Author Secondary Information:	
Order of Authors:	Giuseppe Averta
	Federica Barontini
	Vincenzo Catrambone
	Sami Haddadin
	Giacomo Handjaras
	Jeremia P.O. Held
	Tingli Hu
	Eike Jakubowitz
	Christoph M. Kanzler
	Johannes Kuehn
	Olivier Lambercy
	Andrea Leo

Powered by Editorial Manager® and ProduXion Manager® from Aries Systems Corporation

	Alina Obermeier
	Emiliano Ricciardi
	Anne Schwarz
	Gaetano Valenza
	Antonio Bicchi
	Matteo Bianchi
**Order of Authors Secondary Information:**	
**Response to Reviewers:**	I did all the edits required.
**Additional Information:**	
**Question**	**Response**
Are you submitting this manuscript to a special series or article collection?	No
**Experimental design and statistics**<br><br>Full details of the experimental design and statistical methods used should be given in the Methods section, as detailed in our Minimum Standards Reporting Checklist. Information essential to interpreting the data presented should be made available in the figure legends.<br><br>Have you included all the information requested in your manuscript?	Yes
**Resources**<br><br>A description of all resources used, including antibodies, cell lines, animals and software tools, with enough information to allow them to be uniquely identified, should be included in the Methods section. Authors are strongly encouraged to cite Research Resource Identifiers (RRIDs) for antibodies, model organisms and tools, where possible.<br><br>Have you included the information requested as detailed in our Minimum Standards Reporting Checklist?	Yes
**Availability of data and materials**	Yes

All datasets and code on which the conclusions of the paper rely must be either included in your submission or deposited in publicly available repositories (where available and ethically appropriate), referencing such data using a unique identifier in the references and in the "Availability of Data and Materials" section of your manuscript.<br><br>Have you have met the above requirement as detailed in our Minimum Standards Reporting Checklist?	

Powered by Editorial Manager® and ProduXion Manager® from Aries Systems Corporation

Manuscript

Click here to access/download;LaTeX - Main Document (TeX file);main.tex

GigaScience, 2017, 1–14

doi: xx.xxxx/xxxx
Manuscript in Preparation
Paper

PAPER

U-Limb: A multi-modal, multi-center database on arm motion control in healthy and post-stroke conditions

Giuseppe Averta[1,2,*], Federica Barontini[1,2], Vincenzo Catrambone[1], Sami Haddadin[3], Giacomo Handjaras[4], Jeremia P. O. Held[5], Tingli Hu[3], Eike Jakubowitz[6], Christoph M. Kanzler[7], Johannes Kühn[3], Olivier Lambercy[7], Andrea Leo[4], Alina Obermeier[6], Emiliano Ricciardi[4], Anne Schwarz[5], Gaetano Valenza[1], Antonio Bicchi[1,2] and Matteo Bianchi[1]

[1]Research Center "Enrico Piaggio" and Dipartimento di Ingegneria dell'Informazione, University of Pisa, Pisa, Italy and [2]Soft Robotics for Human Cooperation and Rehabilitation, Fondazione Istituto Italiano di Tecnologia, Genova, Italy and [3]Chair of Robotics Science and Systems Intelligence, Munich School of Robotics and Machine Intelligence, Technical University Munich (TUM), Munich, Germany and [4]MoMiLab Research Unit, IMT School for Advanced Studies Lucca, Lucca, Italy and [5]Division of Vascular Neurology and Neurorehabilitation, Department of Neurology, University of Zurich and University Hospital Zurich, Zurich, Switzerland and [6]Laboratory for Biomechanics and Biomaterials (LBB), Department of Orthopaedic Surgery, Hannover Medical School, Hannover, Germany and [7]Rehabilitation Engineering Laboratory, Institute of Robotics and Intelligent Systems, Department of Health Sciences and Technology, ETH Zurich, Switzerland.

*g.averta3@gmail.com

Abstract

Shading light on the neuroscientific mechanisms of human upper limb motor control, both in healthy and pathological conditions (e.g. after a stroke), can help to devise effective tools for a quantitative evaluation of the impaired conditions, and to properly inform the rehabilitative process. Furthermore, the design and control of mechatronic devices can also benefit from such neuroscientific outcomes, with important implications for assistive and rehabilitation robotics and advanced human-machine interaction. To reach these goals, we believe that an exhaustive data collection on human behavior is a mandatory step. For this reason, we release *U-Limb*, a large, multi-modal, multi-center data collection on human upper-limb movements, with the aim of fostering trans-disciplinary cross-fertilization. This collection of signals consists of data from 91 able bodied and 65 post-stroke subjects and is organized at three levels: (i) upper limb daily living activities, during which kinematic and physiological signals (electro-myography, electro-encephalography and electro-cardiography) were recorded; (ii) force-kinematic behavior during precise manipulation tasks with a haptic device; (iii) brain activity during hand control using functional magnetic resonance imaging.

Key words: Motion Control; Upper Limb; Stroke; Human Kinematics; EMG; EEG; fMRI; VPIT;

Compiled on: May 14, 2021.
Draft manuscript prepared by the author.

Key Points

- A comprehensive dataset on human upper limb during daily-living activities, which encompasses both healthy and pathological (i.e. post-stroke) conditions, to foster a quantitative evaluation of the impaired conditions, and provide guidelines to inform the rehabilitative process.
- The dataset contains multi-modal signals, which consist of kinematic-postural data; physiological recordings (electromyography, electro-encephalography and electro-cardiography); force-kinematic data during precise manipulation tasks with a haptic device; functional magnetic resonance imaging data on hand fine motor control, in imagined, performed and observed manipulation tasks. The goal is to offer a privileged point of view to unveil different yet related aspects of human upper limb motor control.
- Data were acquired at different research and clinical centers, using shared and integrated protocols, to ensure the robustness of the acquired information.

Background

An open access approach to experimental data on human sensory-motor behaviour has become extremely popular in the recent years, not only for neuroscience and clinics, but also for devising new design and control guidelines in robotics. This interest has been strengthened by the widespread adoption of deep learning techniques for analyzing human movements, which has fostered the translation of neuroscientific observations for robot control, design and planning [1]. In literature, it is possible to find a number of datasets focusing on human loco-manipulation, in which data were acquired using different acquisition modalities, ranging from RGB cameras to optical markers and electro-myographic techniques [2, 3, 4, 5, 6, 7, 8, 9, 10, 11]. Among them, it is worth mentioning the KIT Whole-Body Human Motion Database[1], a comprehensive motion capture database of whole-body human motion [12], and the NinaPro database, which consists of surface electro-myography (sEMG) data acquired from 67 intact subjects and 11 amputated subjects, who were asked to perform 50 different movements [13, 14].

Although these datasets represent an important tool for improving the knowledge on the neuroscientific aspects underpinning motor generation and control in humans, their focus was limited on specific acquisition modalities or anatomical parts. Looking at the upper limb as a whole (i.e considering the entire kinematic chain), there is poor or no evidence of databases where multi-modal and multi-center data have been collected. Furthermore, pathological conditions, such as post-stroke subject data, are rarely considered. To the best of authors' knowledge, the only example in literature is the Toronto Rehab Stroke Pose Dataset [15], which consists of upper body 3D poses recorded through Microsoft Kinect Sensors of 9 stroke patients and 10 healthy subjects performing a set of tasks using an upper limb rehabilitation robot.

In this work, we strive to release an exhaustive collection of data related to the neural and local control of upper limb muscle-skeletal system, the *U-Limb* dataset (consisting of 91 able bodied and 65 post-stroke subjects acquired), with the aim of describing upper limb motions in both healthy (i.e participants with no known history of neurological or physical issue) and pathological conditions. The two great novelties of this work are (i) multi-modality and (ii) multi-centricity, i.e. data were acquired at different research and clinical centers, using shared and integrated protocols. The choice of multi-centricity is also motivated by the need for guaranteeing the robustness of the collected data. At the same time, multi-modal acquisitions can offer a privileged point of view to unveil different yet related aspects of human upper limb motor control. For example, kinematic data can shade light on the workspace and the phenomenological characteristics of healthy movements, while offering a benchmarking to comparatively evaluate the severity of the motor impairment. Under this regard it is worth underlining that the postural data contained in the *U-Limb* dataset, which are related to daily living activities, refer to both able-bodied and stroke subjects. These subjects underwent through the same experimental protocol, which also include sEMG and electro-enecphalography (EEG) measurements, to provide information on the level of muscular tone and brain connectivity, respectively, thus offering a unique opportunity to identify quantitative tools for informing and evaluating the rehabilitative outcomes. Furthermore, these different types of information can be used to analyze whether and to which extent the abundance of healthy sensory-motor degrees of freedom of upper limb is organized in low dimensional representations, or synergies, whose study has received a lot of attention in the last decade. More specifically, the main focus of these studies has been on human hands, and it has driven important technological translational outcomes for engineering, assistive and rehabilitation robotics, and advanced human-machine interaction [16]. In parallel to daily living activities, we also report on data that target the observation of precise force-kinematic coordination in manipulation tasks with a robotic device, and functional magnetic resonance imaging (fMRI) data on hand fine motor control in imagined, performed and observed manipulation tasks. In this way, we can provide a comprehensive description of the neuroscientific aspects underpinning motion generation along the whole upper limb kinematic chain, highlighting the different aspects (kinematic, muscular, neural, dynamic) of this process.

These data were collected within the recently ended H2020 EU funded Project SoftPro (grant agreement No. 688857), whose goal was to move from the understanding of the theoretical bases of sensory-motor control of upper limb to produce a strong impact in different fields of research, clinical practice and technology. More details on data organization and collection are provided in the following sections.

Data Description

During the SoftPro Project, we collected different sets of physiological and kinematic data on human upper limb, in both healthy and pathological conditions. The latter refer to post-stroke subjects, whose clinical characteristics are reported later in the text.

Data acquisition followed three experimental protocols, i.e. the lists of tasks the subjects were asked to perform during the acquisition:

1 https://motion-database.humanoids.kit.edu/

- daily-living activities, hereinafter referred to as **SoftPro protocol**;
- hand grasping and control for the fMRI experiments, hereinafter referred to as **fMRI protocol**;
- coordination of arm and hand movements as well as grasping forces during a virtual, goal-directed object manipulation task performed with a haptic device, hereinafter referred to as **VPIT** (Virtual Peg Insertion Test) **protocol**.

The details of each protocol are reported in the dedicated section and subsections.

Data collection was organized to be multi-center and to encompass different acquisition and signal modalities. More specifically, the contributors to the generation of these datasets are: University of Pisa (UP), Istituto Italiano di Tecnologia (IIT), Hannover Medical School (MHH), Technical University of Munich (TUM), University of Zurich (UZH), Swiss Federal Institute of Technology in Zurich (ETHZ) and IMT School for Advanced Studies Lucca (IMT). Data types are:

i. kinematic recordings (optical marker positions or Inertial Measurement Units (IMUs) -based reconstructions of angular values through commercial sensing systems), hereinafter referred to as **KIN data**;
ii. Electro-Myo-Graphy (EMG) signals, hereinafter referred to as **EMG data**;
iii. Electro-Encephalo-Graphy (EEG) signals, hereinafter referred to as **EEG data**;
iv. Electro-Cardio-Graphy (ECG) signals, hereinafter referred to as **ECG data**;
v. functional Magnetic Imaging Resonance (fMRI), hereinafter referred to as **fMRI data**;
vi. kinematic end-effector, grasping force, and haptic interaction data from the VPIT protocol, hereinafter referred to as **VPIT data**.

The details of each experimental acquisition procedure are reported in the dedicated following section.

The information on the able bodied subjects (gender, average age, handedness) who took part at the experimental sessions are briefly summarized in the following:

i Group A: 39 healthy subjects, 17 female, age 26.6 ± 4.2 years, all right-handed, recorded by UP, subjects were tested on the right arm;
ii Group B: 20 healthy subjects, 8 female, age 46.77 ± 15.25 years, 18 right-handed, recorded by MHH, subjects were tested on their dominant hand;
iii Group C: 5 healthy subjects, 2 females, age 59.15 ± 15.85 years, recorded by UZH, subjects were tested on both arms;
iv Group D: 6 healthy male subjects, age 29.17 ± 5.91 years, all right-handed, recorded by TUM, Subjects were tested on the right arm;
v Group E: 27 healthy subjects, divided in three independent groups of nine subjects (5 female) each, all right-handed. Execution experiment: age 29 ± 3 years, imagery experiment: age 27 ± 6 years, observation experiment: age 25 ± 2 years, all recorded by IMT.

The details of the post-stroke subjects involved in the experiments are reported as it follows:

i Group α: 20 post-stroke subjects, 5 female, age 61 ± 10.69 years, 11 right-arm affected, recorded by UZH, subjects were tested on both arms. Note that these subjects are a subset of Group γ, and that the IDs are coherent between the two datasets. Note also that these subjects were collected with the same experimental protocol and by the same experimenter of Group C, and these may serve as control group when using data of Group α.
ii Group β: 20 post-stroke subjects, of which 6 female, age 49.88 ± 16.92 years, 12 right-arm affected, recorded by MHH, subjects were tested on the impaired arm. Note that these subjects were collected with the same experimental protocol and by the same experimenter of Group B, and these may serve as control group when using data of Group α.
iii Group γ: 27 post-stroke subjects, 14 female, age 59.0 ± 10.93 years, 26 right-handed, recorded by ETHZ. Subjects were tested on both arms. Because both the unimpaired and impaired arm were tested in Group γ, we suggest the user to consider the first set of data as control group with respect to the second.

Figure 1. Anatomical placement of active markers, and details on the marker support used for the experiments at UP. Numerical values on the dimensions of marker support are in [mm].

An overview of all the data reported in this publication is finally provided in Tab. 1, where we also indicate the contributor and the details of the Ethical Committee that gave the approval to acquire and share these data in an anonymous form. Additional details on the cohort of subjects enrolled for each group is collected in Tab. 2. All subjects gave written informed consent before the start of the experiment.

Details on the pathology level of stroke subjects

Specific details on the level of impairment for subjects of groups α, β and γ are reported in the accompanying files included in the corresponding dataset directory.

Data Type

KIN data

Kinematic data encompass both (i) optical marker positions and (ii) IMU-based angular reconstructions during the implementation of the Softpro protocol. Regarding (i), we collected different sets of data containing the measurements of 3D optical marker coordinates related to the upper limb movements. Although different across labs, the placement of markers is always sufficient - with a certain redundancy - to enable the estimation of upper-limb movements and the identification of a minimum set of Degrees of Freedom (DoFs), relying on a shared kinematic model (see for example [17]). In the following we provide additional details for each dataset, referring to the id reported in Tab 1.

H_1 Subjects of group A were enrolled in this study. 20 active markers were placed on rigid supports fastened on arm links. In particular, 4 markers were placed on the chest, 6 markers on the arm, 6 markers on the forearm and 4 markers on the hand dorsum. In addition, 20 active markers were also placed on the subject's fingers to track hand movements. Marker 3D position was recorded via a PhaseSpace motion capture system. Marker locations and id are reported in Fig. 1. Subject-specific physical distances between groups of markers and kinematic landmarks are provided in the data folder. See also [17, 18, 19] for further details.

H_4 Subjects of group B were involved in this study. Arm movements were tracked trough 21 passive markers fastened on arm skin. Marker trajectories were captured using an optical infrared motion capturing system based on 12 MX-cameras controlled by Nexus software, Version 1.8.5 (Vicon Motion System Ltd., Oxford, UK) at a sampling rate of 200

id	Type	Group	Protocol	Contributor	Ethical Committee Approval Number
H_1	KIN	A	SoftPro	UP	1072-2016
H_2	EEG	A	SoftPro	UP	1072-2016
H_3	ECG	A	SoftPro	UP	1072-2016
H_4	KIN	B	SoftPro	MHH	3364-2016
H_5	EMG	B	SoftPro	MHH	3364-2016
H_6	KIN	C	SoftPro	UZH	BASEC-ID 2016-02075
H_7	KIN	D	SoftPro	TUM	EV LUH 05/2016
H_8	EMG	D	SoftPro	TUM	EV LUH 05/2016
H_9	EEG	D	SoftPro	TUM	EV LUH 05/2016
H_{10}	fMRI	E	fMRI	IMT	1616/2003(amended), 1072/2016
P_1	KIN	α	SoftPro	UZH	BASEC-ID 2016-02075
P_2	KIN	β	SoftPro	MHH	3364-2016
P_3	EMG	β	SoftPro	MHH	3364-2016
P_4	VPIT	γ	VPIT	ETHZ	EKNZ-2016-02075, EK2017-00398

Table 1. Details on the groups of subjects enrolled in the studies. Ids H_x refer to healthy subjects, while ids P_x to pathological subjects. All the experiments were carried out in accordance with principles of the Declaration of Helsinki, and approved by the Local Institutional Research Ethical Committees. All subjects gave written informed consent before the start of the experiment. Experiments performed at UP were approved by the Ethics Committee of the Area Vasta Nord-Ovest Toscana, Italy; experiments performed at MHH were approved by the Ethics Committee of Hannover Medical School; experiments performed at UZH were approved by the Cantonal Ethics Committee Northwest and Central Switzerland; experiments performed by TUM were approved by the Ethics Committee of Leibniz Universität Hannover, Germany; experiments performed at IMT were approved by the Ethics Committee of the Area Vasta Nord-Ovest Toscana, Italy; experiments performed at ETHZ were approved by the Ethics Committee of ETH Zurich. Note that different id of this table may correspond to the same group of subjects. For example, subjects of Group A were a cohort of 39 healthy participants who performed one single experiment while kinematics, EEG and ECG recordings were simultaneously recorded (ids H_1, H_2, H_3).

Group	Contrib.	Subj. no	Age	M/F	Handedness R/L	Average FMA score
A	UP	39	26.6 ± 4.2	22/17	39/0	N/A
B	MHH	20	46.8 ± 15.3	12/8	18/2	N/A
C	UZH	5	59.2 ± 15.9	3/2	5/0	N/A
D	TUM	6	29.2 ± 6	6/0	6/0	N/A
E	IMT	27	27.0 ± 2	22/5	27/0	N/A
α	UZH	20	61.0 ± 10.7	15/5	19/1	17.8 ± 2.1 (up to 66)
β	MHH	20	49.9 ± 16.9	14/5	12/8	17.8 ± 2.1 (up to 20)
γ	ETHZ	27	59.0 ± 10.9	13/14	26/1	46.6 ± 9.3 (up to 66)

Table 2. Details on the different populations included in this manuscript. For each group of subjects (for details on the modalities please refer to Tab. 1), we report here the Contributor, the number of subjects, their average Age, the gender balance, the Handedness (right vs. left handed)and the average stroke severity in terms of FMA score.

Hz. The marker placement and their IDs are given in figure 2.

H_6 Subjects of group C were involved in this study. The data were recorded with a full-body worn IMU-based system sensor suit (Awinda, Xsens technologies B.V., Enschede, The Netherlands). The system consists of 17 inertial measurement units (IMUs) placed symmetrically on predefined body positions and fixed with Velcro straps and a size-fitting T-Shirt. The IMUs provide 3D angular velocity using rate gyroscopes, 3D acceleration using accelerometers, 3D earth magnetic field using magnetometers, as well as atmospheric pressure using the barometer in an operating frequency 2405 – 1475 MHz. Then, proprietary software was used to reconstruct the time-varying angular deviation (roll-pitch-yaw) between subsequent IMUs. For additional details please refer to the user's manual, which can be found at the following link[2].

H_7 Subjects of group D were involved in this study. Upper-body and shoulder-arm movements were tracked using 9 passive markers, recorded using a Vicon MXT10s (Vicon Motion Systems Ltd, UK, 500 Hz) system with 8 cameras. Please refer to Fig. 2 for details of marker placement.

2 https://www.xsens.com/hubfs/Downloads/usermanual/MVN_User_Manual.pdf

Figure 2. Anatomical landmarks (blue spheres) that define the marker placement for the experiments performed at TUM (H_7). C7 and T8 refer to the vertebrae numbering, the 7th cervical and the 8th thoracical respectively.

Figure 3. Markers' placement used during the experiments performed at MHH (H_4).

To enable the analysis of the effects of stroke conditions in upper limb kinematics (i.e. movements) we recorded the motion of subjects in pathological conditions. More specifically:

P_1 Subjects of group α were enrolled in this study. Arm movements were recorded using the Xsens MVN Awinda system (same setup of H_6). This consists of 17 IMU sensors, placed on the body limbs and trunk, and of a software tool that allows data collection with a frequency of 60 Hz and reconstruct the joint angular values in time, starting from acceleration signals. Part of these data have been used in [20], to which the reader can refer for further details.

P_2 Subjects of group β were involved in this study. Arm movements were tracked trough 21 passive markers fastened on arm skin. Marker placement and data acquisition were the same used for group H2 (see fig. 3).

Figure 4. Placement of EMG sensors following SENIAM guidelines

Figure 5. A schematic representation of HydroCel Geodesic Sensor Net (HCGSN-128), channels location.

Electrode No.	Muscle
1	M. Deltoideus pars clavicularis (DC)
2	M. Biceps brachii (BB)
3	M. Triceps brachii (TB)
4	M. Flexor digitorum superficialis (FDS)
5	M. Extensor digitorum (ED)
6	M. Brachioradialis (BR)
7	M. Flexor carpi ulnaris (FCU)
8	M. Extensor carpi ulnaris (ECU)
9	M. Pronator teres (PT)
10	M. Flexor carpi radialis (FCR)
11	M. Abductor pollicis brevis (APB)
12	M. Abductor digiti minimi (ADM)

Table 3. List of 12 muscles recorded during the experiments at MHH

EMG Data

Muscular data were recorded during the experiments id H_5, H_8 and P_3. More specifically:

H_5 Subjects of Group B were enrolled in this study. A wireless surface EMG (sEMG) system (Trigno Delsys Inc., Natick USA) was used to measure the activity of 12 upper- and forearm muscles with 2000 fps (Tab. 3, see also Fig. 4). Mini sensors were used for smaller muscles (No. 9 – 12) to reduce cross talk artifacts. The 12 bipolar electrodes were placed following the SENIAM guidelines.

H_8 Subjects of group D were involved in this study. Data were collected using a Refa system (TMSi, Netherlands) with 29 bipolar channels. The 29×2 microelectrodes were placed, following the SENIAM guidelines [21], on the muscles reported in Tab. 5 (table is reported at the end of this manuscript).

P_3 Subjects of group β were involved in this study. The experimental framework used is the same of H_5 (see Tab. 3).

EEG Data

Cortical activity was recorded during the experiments id H_2 and H_9. More specifically:

H_2 Subjects of group A were enrolled in this study. Continuous EEG was recorded using a 128-channel Geodesic high-density EEG System (Electrical Geodesics Inc., Eugene, OR, USA) through a pre-cabled HydroCel Geodesic Sensor Net (HCGSN-128), sampling rate of 500 Hz with the vertex as online reference; sensor-skin impedances were maintained below 5-10 kΩ for each sensor. The “ground” sensor on the Net is an “isolated common,” which means it is tied to the zero level or common of the isolated amp circuit’s power supply. A schematic representation of channels location is provided in Figure 5. These data were used for the analyses reported in [22, 23, 24, 25], to which the reader is invited to refer for further technical details.

H_9 Subjects of group D were involved in this study. An actiCHamp active EEG electrode net of 32 unipolar channels (Brain Products GmbH, Germany) – which corresponds to the 10-20 system [26] – was used at 10 kHz to record brain activity.

ECG Data

Heart electrical activity was recorded during the experiment id H_3. More specifically:

H_3 Subjects of group A were enrolled in this study. Continuous ECG was recorded using the Polygraph Input Box (PIB), the EGI’s physiological measurement Geodesic System (Electrical Geodesics Inc., Eugene, OR, USA). It allows the simultaneous measurement of peripheral nervous system activity and EEG, indeed the acquisition was performed together with experiment ids H_2 and H_1. The PIB includes a bipolar channel inputs for the measurement of ECG. The input box accommodates the most common sensor connector (the 1.5 mm female safety connector) that is used in both clinical and research settings. Signals were acquired with a sampling rate of 500 Hz, applying two standard ECG sensors, the first to the lower left ribcage and the second to the upper right collarbone/clavicle, accordingly to the constructor design.

VPIT Data

Kinematic and haptic interaction data were transferred through a FireWire connection from the end-effector to a personal computer. Grasping force data were recorded through a NI (National Instruments, Austin, USA) Data Acquisition Card. The virtual reality environment of the VPIT was implemented in C++ and OpenGL. All data were sampled at 1 kHz. Missing data segments, which occurred due to a delayed communication of the C++ software, of at least 50 samples were linearly interpolated. Further, the sensor readings were low-pass filtered with a zero-phase Butterworth filter of second order and 10 Hz cut-off frequency. As the VPIT comprises multiple movement phases with different characteristics, a temporal segmentation of the continuous data streams is required to select specific parts of the movements that are relevant to describe impairments in the targeted sensorimotor functions. In more detail, the *transport* (ballistic movement after picking up a peg) and *return* (ballistic movement after releasing a peg in a hole) phases focus especially on the gross movements of the task. The start and end of these phases were identified by the moment the cursor velocity increased above and dropped below 5% of peak velocity, respectively. To quantify fine target adjustments when reaching for a target or hole, the data was segmented into the *peg approach* and *hole approach* phases. Lastly, the grasping force data was additionally divided into the *force buildup* and *force release* phases. These periods were detected by first identifying the largest maximum/minimum in the force rate profile and subsequently quantifying when the force rate dropped below and raise above 10% of the maximum/minimum force rate. More details about the data processing can be found in previous work [27].

FMRI

All fMRI data were acquired using a Philips Ingenia 3-Tesla scanner, with a 12-channel head phased array coil. Data consisted of anatomical and functional images. For anatomical images, a MP-RAGE sequence was acquired, with TR = 7 ms, TE = 3.17 ms, Flip Angle = 9°, Field of View = 224x224 mm, 156 sagittal slices, voxel size = 1x1x1 mm. To acquire functional images, a Gradient-Echo EPI sequence was used, with TR = 200 ms, TE= 30 ms Flip Angle = 75°, SENSE acceleration, factor = 2.5, Field of View = 256x256 mm, 38 interleaved axial slices, acquisition voxel size = 3x3x3 mm. Images were reconstructed with a 128x128 matrix, reconstructed voxel size was 2x2x3 mm. The top-to-bottom extent along the Z-axis was 114 mm; this ensured total brain coverage, excluding part of the cerebellum. Functional runs comprised four additional

dummy volumes, discarded by the scanner and not transferred.

Structural images were anonymized with mri_deface [28], in order to remove any anatomical detail that can allow subjects' identification. For functional MRI, the initial stages of preprocessing and the estimation of single-subject BOLD responses were performed using AFNI [29] and FSL 5.01 [30]. First, all fMRI data underwent removal of signal spikes, temporal realignment of slices, rigid-body registration to the mean image of the first run and estimation of the six motion parameters. Motion spikes were then estimated as time-points exceeding 0.5 mm of Framewise Displacement (FD) [31]; iterative spatial smoothing up to 4 mm Full Width at Half Maximum (FWHM) was subsequently performed, and the signal of each run was expressed as a percentage of the mean. Afterwards, stimulus-evoked fMRI responses were estimated for each task using a General Linear Model: the onsets of the five repetitions of each stimulus were entered into the model as regressors of interest, and the six motion parameters plus the raw value of the FD metric and polynomial trends up to the fourth order were used as regressors of no interest. The five repetitions of each stimulus were combined; for the execution and imagery experiments, we modeled the entire stimulation period (0-16 seconds) with nine tent functions peaking at 2.5 seconds. The average t-score maps from the fifth, sixth and seventh functions, that covered activity from two to six seconds after movement onset, were used as estimates of movement-relaed BOLD activity. A standard block function, convolved with the hemodynamic response, was used for the observation experiment; the modeled function started with the presentation of the video clip, and lasted one second. To avoid that baseline fMRI activity could reflect the two-alternatives task, this was modeled with a 2 seconds-long block function and the estimated BOLD responses were discarded. T-score maps from the tent functions (for the execution and imagery experiments) and from the block functions relative to the movie clip (for the observation experiment) were selected for data sharing.

Experimental setup differences among research centers

All the data acquisitions were preformed according to an integrated set of protocols. For what concerns the SoftPro protocol, the different research centers shared the same list of actions. However, specific cases required some adaptation of the general framework. Differences with respect to the general setup are reported in this section.

- Experiments of Group D were carried out inside an electromagnetically isolated chamber. For this reason, subjects were not able to execute task 22 (tennis smash) of the Softpro protocol. This task was replaced with the following one: *Reach and grasp a smartphone, unlock the screen, dial a number, and put it back to the initial position.* See also [32] for additional details.

Analyses & Technical Validation

Kinematic data

Quality of kinematic data has been tested through the evaluation of Signal to Noise Ratio (SNR).

ID H_1

Data of these experiments were collected using the PhaseSpace motion capture system, a commercial device that tracks precise motion data with sub-millimeter resolution (the amount of static marker jitter is less than 0.5 mm, usually 0.1 mm). 10 stereo-cameras were placed around the subject so to fully cover the scene (360 deg). The system was fully calibrated before the acquisition of each subject, following the standard procedure described by the manufacturer. Markers ID are automatically associated by the proprietary software tool. For these data, we quantified the SNR by selecting the three seconds of rest before the execution of each task to estimate measurement noise, and a sample of three seconds of signal during the execution of the task itself (vectors of same length). We used for this analysis one marker placed on the hand dorsum, i.e. the worst case scenario because of the reduced distance between markers. Then, from the x,y,z vectors of markers' trajectories we calculated the norm and removed the mean. From signal and noise vectors, SNR was calculated through the Matlab `snr` routine. We randomly selected 20 trials from the dataset and quantified the SNR for each sample. We obtained a median value equal to 37.54, interquartile range 4.56. These data were used for kinematic reconstructions that were employed for the principal component analysis and functional principal component analysis, which outcomes are discussed in [19] and [17], respectively. The reader can refer to those works for an example on how to pre-process and analyze the data. We also report a pseudocode (see Alg. 1) of the motion identification procedure employed in [17] to calculate joint angular values from readings of the motion capture system. This should serve as an example of data analysis that can be tailored on different acquisition systems.

ID H_4

Data of these experiments were collected through the Vicon motion capture system, a commercial device that ensure sub-millimeter errors in static conditions (see [33]). 12 cameras were used to record the scene from multiple perspectives. Marker labelling and trajectory reconstruction were performed through the proprietary software Nexus v1.8.5. For these data, SNR was quantified following the same procedure of H_1. From a random selection of 20 trials, we obtained a median value equal to 44.12, interquartile range 3.09.

ID H_6

Data of these experiments were collected through a IMU-based sensor suit, a commercial device by Xsens technologies B.V., Enschede, The Netherlands. The producer declares an accuracy in angles estimation of 0.2 deg for roll/pitch and 0.5 deg for heading angles in static conditions. These values are increased to the value of 1 deg in dynamic conditions. The whole acquisition system was properly calibrated, following the manufacturer's guidelines, before the acquisition of each subject. For these data, we quantified SNR following the same procedure used with the previous cases. SNR was evaluated on the norm of roll/pitch/yaw angles of the arm w.r.t. the chest (shoulder DoFs). Our analysis on a random selection of 20 trials reported a median value equal to 40.73, interquartile range 5.87.

ID H_7

Data of these experiments were collected through a Vicon motion capture system, similar to the one used in H_4. As previously stated, this system ensure sub-millimeter errors in static conditions (see [33]). Also in this case we quantified the SNR of data associated to the 3D position of markers placed on the hand dorsum. Our analysis on a random selection of 20 trials resulted in a median value equal to 45.0, interquartile range 6.48.

EMG data

All the experiments which involved the recording of EMG data were performed by expert experimenters who followed the SENIAM guidelines for skin preparation and electrodes placements [34]. This represents a gold standard in EMG signals

Algorithm 1 Pseudocode for the motion identification procedure used in [17]

```
procedure Setup
    X ← Load Markers data                         ▷ Load 3D position of markers
    Mod ← Load Kinematic model                    ▷ e.g. following Denavit–Hartenberg parametrization
    FK ← ForwardKinematics(Mod)                   ▷ define a model-based map from joints angular values to 3D markers positions
    Set KinPars IG                                ▷ Initial Guess of Mod parameters
    Set KinPars LB                                ▷ Lower Bound of Mod parameters
    Set KinPars UB                                ▷ Upper Bound of Mod parameters

procedure Model Calibration
    X_r ← Rotate(X)                               ▷ Rotate X w.r.t reference markers on the chest
    OptPars ← InteriorPoint(X, FK, IG, LB, UB)    ▷ Find optimal kinematic parameters using Interior Point method

procedure Motion Identification
    Set KalmanPars                                ▷ Set parameters for Extended Kalman Filter (EKF)
    i = 1
    N ← Size(X)                                   ▷ Number of samples in X
    do
        X_i ← Select(X_r,i)                       ▷ Pick i-th sample from X
        q_i ← EKF(X_i, OptPars, FK, KalmanPars)   ▷ Identify joint angular values with EKF
        Q ← Append(q_i)                           ▷ Build a dataset of joint angular values
        i++
    while i ≠ N
```

recordings and treatment, which guarantees the highest data quality. Before the placement of EMG sensors, the corresponding skin areas were cleaned through abrasive and conductive cleaning pastes (skin impedance was controlled < 30kΩ). Before each acquisition, the recorded data were carefully visually checked on-line by an expert experimenter, and sensor locations were adjusted if necessary. Part of these data were successfully used for the identification of task-dependent muscle synergies in [32] and for the validation of a human shoulder-arm musculoskeletal dynamic model in [35], to which the interested reader is referred for an example on how to pre-process and analyze the data. It is worth mentioning that in literature EMG data typically undergo through a number of pre-processing steps to increase the quality of the collected signal and make it usable for further analyses. Since in this publication we are releasing raw data, it is difficult to find references for quantitative SNR calculated on raw data. To evaluate the SNR on the raw data released with this publication, we first performed a high-pass filtering on each bipolar channel (4th order Butterworth filter, cut-off frequency equal to 10Hz) to remove baseline shifts. Then, we calculated the SNR for each sample and for each bipolar channel. The estimation of the SNR is based on [36], a Matlab implementation can be found at the link below [3]. This evaluation of the SNR defines the noise as an unidentifiable high frequency component concentrated on the upper 20% of the frequency range (ensuring all frequencies are above 500 Hz). The module of the noise is then estimated as the average of all the power densities in the upper 20% frequency range. Then, the SNR is estimated as the ratio between the sum of all the power densities and the noise. In the data released with this publication, the median value of the SNR is always higher than 10^2.

EEG

EEG data presented were already successfully exploited in different works and from different perspectives [22, 23, 24, 25]. As well known, many different pre-processing pipelines were presented in literature to properly analyse EEG signals, they can vary according to the specific further analyses that are intended to be performed on the dataset. For this reason, in [22, 23, 24, 25] different processing steps were applied to remove artifacts and prepare the data for further analyses. A detailed description of the processing steps that have been implemented is thereby provided.

VPIT

The VPIT test is based on a CE marked haptic device, i.e. PHANTOM Omni, SensAble Technologies, Inc., USA, with a nominal position resolution higher than 450 dpi (0.055 mm). Grasping forces are recorded through three single-axis force sensors (CentoNewton 40, EPFL, Switzerland). Each sensor can accurately record force values in the range of 0 – 40 N, with a resolution of 0.05 N. The linear relationship between forces applied and voltages produced by the force sensors has been verified in [37]. To do this, the sensor was dynamically loaded and unloaded (up to 100 N/s) to three force levels (approximately 10, 20 and 30 N) against a commercial load cell (Mini 40, ATI Industrial Automation, USA) while the voltage output of the piezoresistive sensor was measured. Force data were lowpass filtered at 50 Hz and show good linearity characteristic ($V = 0.0915F + 0.726$; $R^2 = 0.9987$, where F is the applied force and V the voltage measured by the force sensor).

fMRI

Quality check of fMRI data was performed using MRIQC [38]. MRIQC is a software package, part of the bids-apps [39] that performs several processing steps in order to derive different parameters regarding image quality, such as SNR measures, motion estimates (e.g., framewise displacement) that are graphically reported as as Image Quality Metrics (IQMs) from each run in each subject. Here, we ran MRIQC on raw functional data, and plots with IQMs and mean images from single runs are included in the QC folder, which is organized in the same way as the folder containing data. Group analysis – i.e., averages and distributions of IQMs across subjects – are also included. For further information on the quality check pipeline, please refer to http://mriqc.org.

3 http://www.sce.carleton.ca/faculty/chan/matlab/matlab_library.htm

Discussion and Potential Implications

The aim of this paper is to provide an exhaustive description of the experimental protocols and acquisition techniques that finally led to the release of the dataset U-Limb. This dataset has a value *per se*, since it represents an extraordinary and unique source of information, with multiple sensory modalities that concur to shade light on different aspects underpinning the motor control of human upper limb. We do firmly believe that the release of this dataset, together with all the information needed to reproduce the experiments, can be a key component for fostering data re-usage and benchmarking, and finally advancing the research in the field of motor control. The objective is to contribute to the establishment of a trans-disciplinary community and to the definition of well-accepted guidelines for data collection. Of note, some of the data reported in this paper have already been employed and analyzed for different research purposes, and the scientific outcomes has impacted or could positively impact various fields, as already mentioned in the introductory part of the paper. In the following we report some examples of the applications of our data and discuss the trans-disciplinary impact. First and foremost, neuroscientific research can benefit from the analysis of U-Limb data. Thanks to the adoption of integrated experimental protocols, the kinematic, muscular, dynamic mechanisms, as well as the central and autonomous nervous system components related to motion execution can be investigated, at different levels of the kinematic chain (e.g. fMRI data focuses on the hand; kinematic data focuses on the whole upper limb chain). In [17] a functional Principal Component Analysis (fPCA) was applied to the kinematic data of healthy subjects, labelled as H_1, to identify the principal functional modes of human upper limb movements. Long story short, the idea was to decompose the temporal trajectories of upper limb joints in terms of a basis of functions. The results showed that a combination of few functional principal components is sufficient to reconstruct a large part of the variability of joint evolutions over time, in activities of daily living. This observation has led to the definition of a planning problem for the generation of human-like movements in robot manipulators. Briefly, the human upper limb principal motion modes computed through functional analysis were embedded in the robot trajectory optimization, thus intrinsically ensuring robot human-likeness in free motions and for obstacle avoidance [40, 41]. This point is of paramount importance in advanced human-robot interaction and assistive applications, to guarantee the safety of the human operator and the acceptability of the robotic technologies [42]. The kinematic data labelled as H_1 were also analyzed in [19], to characterize the upper limb poses at each time frame, through a technique that was named Repeated-Principal Component Analysis (R-PCA). The outcomes demonstrated that the subspace identified by the first three principal components takes into account most of the motion variability, and these results were proven to be stable over time and consistent across subjects. These finding could inform the definition of control laws for upper limb robotic devices, relying on a time-invariant low-dimensional approximation of upper limb kinematics, within the general framework of synergistic control [16]. For what concerns the kinematic data on post stroke subjects, it is worth reporting the results described in [20]. Briefly, the data labelled as P_1 were analyzed to evaluate the variations of functional principal components applied to the reconstruction of joint angle trajectories. These variations were compared between two conditions, i.e. the affected and non-affected arm, to devise a dissimilarity index for achieving an accurate and quantitative assessment of upper limb motion impairment induced by stroke. This point is extremely important to overcome the limitations of current evaluation procedures, which are mostly based on ordinal scaling, operator-dependent, and subject to floor and ceiling effects, to pave the path for a more analytical assessment that could inform the rehabilitation procedures. On the same line, the kinematic and haptic interaction data labelled as P_4 were used to devise quantitative metrics to evaluate the neurological sensorimotor impairment of upper limb kineto-dynamic behavior, in virtual peg-in-hole tasks [43]. It is worth highlighting here one of the characteristics that make the U-Limb dataset unique: i.e. the possibility to have data that cover different yet related aspects of human upper limb motor control, which allow to analyze it under different perspectives and points of view (for the examples reported before, a purely kinematic point of view for P_1, the kineto-dynamic coordination in virtual manipulation tasks for P_4). Considering the EEG data labelled as H_2, in [22, 23, 24] they were used to automatically discriminate transitive, intransitive and tool-mediated imaginary actions (as described in the *Softpro protocol*) using EEG dynamics, and relying on non linear support vector machine and Fuzzy Entropy techniques. Interestingly, in [24] different combinations of EEG-derived spatial and frequency information were investigated to find the most accurate feature vector, and gender differences between accuracies achieved with male and female data were observed. These results could open to gender-based models for the development of optimized brain machine interfaces. To conclude, U-Limb can positively impact different research fields, which encompass neuroscience and motor control; clinical assessment and rehabilitation; robotics and advanced human machine interfaces.

Methods

Experimental Protocols

SoftPro Protocol

Activities of Daily Living is a term commonly used in rehabilitation to indicate a set of everyday tasks. More recently, the usage of this class of movements has become central also in robotics to evaluate the usage of artificial systems in daily actions. The criteria for the selection of a comprehensive list of activities include (i) the specific hand grasping configuration and (ii) the direction of motion for the whole upper limb.

In the attempt of exhaustively consider all the possible combinations of (i) and (ii), we identified 30 tasks, which were divided in three different classes: intransitive, transitive and tool-mediated actions. Intransitive tasks collect movements without contact with external objects, Transitive tasks are actions which involve an external object and, finally, tool-mediated tasks are actions in which an object is used to interact with another object. This particular classification takes inspiration from the analysis presented in [44], which was proven to be reflected at the cortical level in imaging studies, e.g. [45], that show differences in cortical activation between actions belonging to the three different classes, with prefrontal and parietal regions of the left hemisphere tuned towards tool-mediated and transitive actions, whereas the right hemisphere shows a preference for meaningful, intransitive gestures. This organization has been confirmed by clinical observations as well: classic neurological studies show that, following cortical stroke, patients can develop class-specific deficits for tool-mediated actions [46, 47], and deficits for transitive or intransitive gestures have been described as a result of greater involvement of the left or right hemisphere, respectively [48].

Within a specific class, the selected actions cover different hand grasping configuration in order to span most of the postures of the main hand grasping taxonomies [49, 50]. A detailed list of actions is reported in table 4. In each row of the table, the first element reports the task number, the second

Table 4. List of action that defines the SoftPro protocol. The label *Int* stands for Intransitive tasks, the label *Tr* for Transitive tasks and the label *T-M* for Tool-Mediated tasks.

#	# [49]	Class	Description
1		Int	Ok gesture (lifting hand from the table)
2		Int	Thumb down (lifting hand from the table)
3		Int	Exultation (extending the arm up in the air with closed fist)
4		Int	Hitchhiking (extending the arm along the frontal plane, laterally, parallel to the floor, with extended elbow, closed fist, extended thumb)
5		Int	Block out sun from own face (touching the face with the palm and covering the eyes)
6		Int	Greet (with open hand, moving wrist) (3 times)
7		Int	Military salute (with lifted elbow)
8		Int	Stop gesture (extending the arm along the sagittal plane, parallel to the floor, open palm)
9		Int	Pointing (with index finger) of something straight ahead (with outstretched arm)
10		Int	Silence gesture (bringing the index finger, with the remainder of the hand closed, on the lips)
11	2	Tr	Reach and grasp a small suitcase from the handle, lift it and place it on the floor (close to own chair, along own sagittal plane)
12	3	Tr	Reach and grasp a glass, drink for 3 seconds and place it in the initial position
13	4	Tr	Reach and grasp a phone receiver, carry it to own ear for 3 seconds and place it in the initial position
14	6	Tr	Reach and grasp a book (placed overhead on a shelf), put in on the table and open it (from right side to left side)
15	8	Tr	Reach and grasp a small cup from the handle (2 fingers + thumb), drink for 3 seconds and place it in the initial position
16	11	Tr	Reach and grasp an apple, mimic biting and put it in the initial position
17	12,13	Tr	Reach and grasp a hat from its top and place it on own head
18	12	Tr	Reach and grasp a cup from its top, lift it and put it on the left side of the table
19	15	Tr	Receive a tray (straight ahead, with open hand) and put it in the middle of the table
20	16	Tr	Reach and grasp a key in a lock (vertical axis), extract it from the lock and put it on the left side of the table
21	1	T-M	Reach and grasp a bottle, pour water into a glass and put the bottle in the initial position
22	2,3,4	T-M	Reach and grasp a tennis racket (placed along own frontal plane) and play a forehand (the subject is still seated)
23	5	T-M	Reach and grasp a toothbrush, brush teeth (horizontal axis, one time left-right) and put it inside a holder (on the right side of the table)
24	6	T-M	Reach and grasp a laptop, open it (without changing its position) (4 fingers + thumb)
25	7,8,9	T-M	Reach and grasp a pen (placed on the right side of the table) and draw a vertical line on the table (from the top to the bottom)
26	7	T-M	Reach and grasp a pencil (placed along own frontal plane) (3 fingers + thumb) and put it inside a squared pencil holder (placed on the left side of the table)
27	9	T-M	Reach and grasp a tea bag in a cup (1 finger + thumb), remove it from the cup and place it on the table on the right side of the table
28	10	T-M	Reach and grasp a doorknob, turn it clockwise and counterclockwise and open the door
29	13	T-M	Reach and grasp a tennis ball (with fingertips) and place it in a basket on the floor (right)
30	14	T-M	Reach and grasp a cap (2 fingers + thumb) of a bottle (held by left hand), unscrew it and place it overhead on a shelf

links to the grasp taxonomy [49], the third indicates the class of movement and, finally, the fourth reports a brief description of the task. More details can be found in [19]. During the experiment, each task was repeated at least three times, resulting in a minimum number of 90 independent acquisitions for each subject. The temporal timeline for task execution was: 1) three seconds of rest, 2) task execution at a self-paced speed, 3) three seconds of rest. Regarding UP (IDs $\{H_1, H_2, H_3\}$) a custom C++ routine was used to associate the pressure of a keyboard key with: i) the start of 3D markers position acquisition and ii) the placement of a temporal marker in the acquisition flow of EEG/ECG recordings. The same tool was used to interrupt the task acquisition on both sides. Absolute timing is also provided in the related dataset. An analogous procedure was used at MHH (IDs $\{H_4, H_5\}$ and $\{P_2, P_3\}$), and at TUM (IDs $\{H_7, H_8, H_9\}$), where an EtherCAT system with NI 9144 (National Instruments) controlled using the Simulink tool of Matlab, was employed to send start/stop trigger signals to the acquisition systems.

VPIT protocol

The VPIT is performed using a commercial haptic end-effector (PHANTOM Omni, 3D Systems, CA, USA), a custom-made handle with force sensors (CentoNewton40, EPFL, Switzerland), and a virtual reality environment rendered on personal computer (Figure 6) [37, 51, 52, 53]. The VPIT requires the insertion of nine virtual pegs into nine virtual holes through the coordination of arm and hand movements controlling the end-effector as well as the grasping forces applied to the instrumented handle attached at the end-effector. In more detail, a virtual cursor needs to be first spatially aligned with the virtual peg. Subsequently, a peg can be picked up and transported towards a hole by applying a grasping force of at least 2 N. The peg can be released in the hole by reducing the grasping force below the threshold. The virtual pegboard is thereby physically rendered through the haptic device to ease the perception of the 3D virtual reality environment.

Figure 6. The Virtual Peg Insertion Test (VPIT) is a technology-aided assessment platform consisting of a haptic end-effector, a grasping force sensing handle, and a virtual reality environment. It allows to record kinematic and kinetic data about sensorimotor impairments in arm and hand during a functional task.

The starting position of the subjects was defined through an elbow flexion angle of ≈ 90 deg, a shoulder abduction angle of ≈ 45 deg, and a shoulder flexion angle of ≈ 10 deg. The protocol consists of an initial familiarization period, during which subjects were instructed to perform the task as fast and precise as possible, followed by five repetitions of the task (i.e., inserting all nine pegs five times). More details about the setup and

Electrode No.	Muscle
1	M. trapezius Pars descendens (TRPc)
2	M. trapezius Pars transversa (TRPt)
3	M. trapezius Pars ascendens (TRPa)
4	M. deltoideus Pars clavicularis (DLTc)
5	M. deltoideus Pars acromialis (DLTa)
6	M. deltoideus Pars spinalis (DLTs)
7	M. latissimus dorsi (LTDt)
8	M. pectoralis major Pars clavicularis (PMJc)
9	M. pectoralis major Pars sternocostalis (PMJs)
10	M. pectoralis major Pars abdominalis (PMJr)
11	M. biceps brachii Caput longum (BICl)
12	M. biceps brachii Caput breve (BICs)
13	M. triceps brachii Caput longum (TRClg)
14	M. triceps brachii Caput laterale (TRClt)
15	M. pronator teres (PRNT)
16	M. flexor carpi radialis et (if present) M. palmaris longus (FCR)
17	M. flexor carpi ulnaris (FCU)
18	M. flexor digitorum superficialis (FDS)
19	M. flexor pollicis longus (FPL)
20	M. extensor digitorum (EDT)
21	M. extensor digiti minimi (EDM)
22	M. extensor carpi ulnaris (ECU)
23	M. abductor pollicis longus et M. extensor pollicis brevis (APL&EPB)
24	M. brachioradialis (BRD)
25	M. extensor carpi radialis (ECR)
26	M. abductor digit minimi (ADM)
27	M. flexor pollicis brevis (FPB)
28	M. abductor pollicis brevis (APB)
29	M. interosseus dorsalis I (DI1)

Table 5. List of 29 muscles recorded during the experiments at TUM (id H_8)

the procedure can be found in previous work [37, 27].

fMRI protocol

Design for motor execution and imagery experiments were based on a previous work [54], and relied on a delayed grasping task after a visual presentation of the target objects. More specifically, in each trial, a picture of the target object was visually presented for 2 seconds, then, after a 4 seconds pause, an auditory cue prompted the actual task: subjects had to preshape the hand as if they were grasping the target object to use it (for the execution group) or imagine a preshaping movement, without moving their hand (for the imagery group). A 10 seconds interval separated two subsequent trials. Twenty different target objects were used for this study, as in (see Table 6 for a list) and, in each experiment, movements were repeated five times, for a total number of 100 trials, organized in five fMRI runs, each lasting 5′44″ minutes, including twelve seconds of rest at the beginning and at the end of each run to achieve a measure of baseline fMRI activity. The experimental paradigm for execution and imagery experiments was coded using Presentation (Neurobehavioral System, Berkeley, CA, http://www.neurobs.com), and presented with a MR-compatible monitor at the resolution of 1200x800 pixels, and a mirror mounted on the MR coil. During the observation experiment, subjects watched short videos of preshaping movements towards an object from the same set adopted in the other experiments. In each trial, the video was followed by a task that implied a judgment on the target of the preshaping gesture. To create videos, we used vectors of joint angles (according to a 24 DoFs model) corresponding to the common starting posture and to the twenty final object-specific postures, recorded in a previous study [54]. Intermediate hand

Bucket	Calculator	Chalk	Cherry
Dinner plate	Espresso cup	Fishing rod	Frisbee
Hairdryer	Hammer	Telephone handset	Jar lid
Light bulb	Pc mouse	PenRope	Ice cube
Tennis racket	Toothpick	Wrench	

Table 6. List of objects used for the fMRI experiments.

configurations (i.e., posture vectors) between the initial and final postures were obtained from linear interpolation between the values of each kinematic joint angle in the initial and final hand postural configurations. The resulting 30 vectors of joint angles were plotted as 3D renderings, using Mathematica 8.0 (Wolfram Research Inc, Champaign, IL, USA), saved as png images (size: 800x600px), and converted to one second-long videos at a frame rate of 60 Hz. Five sets of 20 videos were created, showing the hand rendering as seen from five different viewpoints, obtained by changing the values of azimuth and elevation. During the fMRI experiment, subjects performed five runs, each comprising 20 trials. During each trial, the video was presented (1 second), followed by a black fixation cross at the center of the screen (7 seconds). Then, the judgment task (two-alternatives forced choice) was presented, and subjects were shown the black/white pictures of two objects (size: 250x250 px) – the target of the preshaping gesture previously shown and a randomly-chosen alternative – and asked to press the left or right key on a MR-compatible keyboard to select the actual target of the preshaping movement. After the task, the same black fixation cross was shown for 6 seconds. Each run comprised the presentation of the full set of twenty videos (20 objects), always from the same viewpoint; the five different viewpoints were presented in separate runs. Each run started and ended with 10 seconds of rest, and lasted in total 5 minutes and 40 seconds. The experimental paradigm was delivered with a MR-compatible monitor at the resolution of 1200x800 pixels, and a mirror mounted on the MR coil, using the e-Prime 2 software package (Psychology Software Tools, Pittsburgh, PA, USA). Due to hardware failure, behavioral responses from two subjects could not be recorded. For all experiments, subjects performed a familiarization run, outside the MR scanner, to ensure that they correctly understood the procedures.

Data Records

Data records published with this paper, together with the dataset summary and ReadMe, are available through the Harvard Dataverse repository. Data can be downloaded through the link in footnote [4]. The overall size is 36.2 GB. Data are organized in 6 folders, one for each research center. Within every folder, data are organized per recording modality (e.g. kinematic data, EMG and EEG). Data provided from each institution have been separately compressed in `.rar` format and uploaded on the repository, in such a way to enable the download of single block of data. For block heavier than 2.5 Gb, we divided the file in multiple linked parts. In these cases, to properly unpack the data the reader is required to extract the file named XXX.part1, which in turn will automatically recall the subsequent parts. Each folder contain a ReadMe file that details the folder content. In the following, we provide more detailed information for each folder.

4 https://dataverse.harvard.edu/privateurl.xhtml?token=d8ce17bf-70a5-4f43-b3e0-8b4ea333bdcf

Folder UP

In this folder, data are organized per recording modality, i.e. EEG-ECG and KIN. Each folder contains in turn 39 folder named "SXX", where XX is the subject ID. The folder Data_KIN contains the kinematic acquisition. Files are named as "SXX_Y_Z", where Y is the Task number and Z is the repetition number (e.g. *S4_23_1*). Data are collected with a sampling rate equal to 100 Hz. Each acquisition is provided in the dedicated mat file.

An identical naming has been used for the corresponding (synchronized) data of EEG-ECG signals, contained in the "Data EEG - ECG" folder. This folder contains the MFF files with the EEG and ECG data (in milliVolt(mV)). Data were gathered through EGI 128-channel system (sampling rate 500 Hz). Each acquisition is complemented with a number of markers that identify the beginning of each repetition of a single task.

Note that, for these experiments, three repetitions of the same task are provided. There are some cases in which the Z value (repetition number) is higher than 3. This can be associated to cases in which we noticed: i) errors in the task execution; ii) evident problems in the acquisition (either in kinematic data or in EEG data). In these cases, we performed additional repetitions to guarantee the minimum number of three samples of the same task. Acquisitions containing evident errors have been discarded from the dataset.

In addition, two additional folders are included, namely "read_EEG" and "read_plot_KIN ", in which we provide sample codes to access and plot the dataset. Further information about the data and the code are included in the ReadMe file.

Folder MHH

In this folder, data are organized per recording modality, i.e. EMG data and Kinematic data. These two subfolders are divided in Healthy and Stroke subjects.

Trials are named through 3 numbers (e.g. 10_8_3) where the first number (in the example 10) indicates the subject id, the second (8 in the example) indicates the task number and, finally, the third is the trial number. Stroke subjects are named with the same policy with and additional "S" at the beginning of the name.

EMG data are organized to have the rows corresponding to the time frames (sampling rate 2000 Hz), and the columns associated to the 12 measured muscles. The kinematic data files contain the position data of thorax, upper-arm and forearm markers. The table is divided in 63 columns - with 3 columns, corresponding to the x, y, z position, for each of the 21 markers; the rows, starting from the 3rd one, report the recorded marker position for each time frame (sampling rate 200 Hz). The first two rows contain respectively the marker names and the measure unit (in mm).

The file *read_emgfiles.m* is a sample Matlab code to plot the EMG data. Additional details are provided in the ReadMe file. Subject-specific information are provided in one additional file, named “Patients_details_MHH_extended.doc”. There we reported the following characteristics: ID, Age, Gender, Tested limb, Impaired limb, Dominant limb, Time since stroke, FMA-UE and MM score.

Folder UZH

In this folder, data are organized for each subject who took part to the experiment, i.e. healthy and impaired subjects. Kinematic parameters are stored in software specific XML file format (.mvnx) that enable the import to different software tools, such as MATLAB and Microsoft Excel. Each mvnx-file represents one trial execution and is named according to the subject ID (e.g. P02), task number (T01-T30), tested upper limb (R/L) and repetition (1-3). Sample Matlab codes are provided, showing how to access and plot data. More information regarding the file structure and how to plot data are provided in the ReadMe file. Subject-specific information are provided in the additional file “ParticipantCharacteristics.xlsx”. There we reported the following information: ID, Age, Gender, Impaired limb, Dominant limb, Time since stroke, FMA-UE. Note that the 20 stroke subjects enrolled in this dataset (Group α) are a subset of the 27 who performed the VPIT protocol (Group γ). Therefore, further information on subjects of this folder may also be included in the additional files included in Folder ETHZ (the ID of subjects is coherent in the two datasets).

Folder TUM

In this folder, data are organized per subjects. Each subfolder is divided per recording modality, i.e. EEG, EMG, and Kinemaitc Data (MoCap folder). Matlab files are provided to access and plot data (i.e. *plot_KIN.m*, *plot_EMG.m* and *plot_EEG.m*).

Folder ETHZ

In this folder, the provided `VPIT_Data_v3.mat` file contains processed and unprocessed VPIT data collected from 27 chronic post-stroke individuals. Data are all contained in the *VPIT_Data_v3.mat* file, in which each row corresponds to data from one specific trial. The file *header.xlsx* contains detailed meta information regarding the content of each column in the `VPIT_Data_v3.mat`. Additional information about the data, processing and procedures are provided in the ReadMe file. Subject-specific information are provided in two additional files, named “patient-information.png” and “patient-information-2.png”. There we reported the following characteristics: ID, Age, Gender, Tested limb, Impaired limb, Dominant limb, Time since stroke, FMA-UE, ARAT, NHPT, BBT, MAS, EmNSA and MOCA.

Folder IMT

In this folder, data are organized according to the Brain Imaging Data Structure (BIDS) standard [55]. Single-subject t-score maps from functional data are included in the directory for processed data (i.e., derivatives). For the execution and imagery experiments, t-scores for the fifth, sixth and seventh tent functions (i.e., with peak at 2, 4, 6 seconds after movement onset) are selected. Each stimulus is modeled using its five repetitions. The `.nii.gz` file contains the average of the three selected t-score maps. For the observation experiment, t-scores for the block functions, covering the stimulus period, are selected. Each stimulus is modeled using its five repetitions. The 2AFC task responses, though modeled, were discarded. The `.nii.gz` file contains the twenty t-score maps, one for each stimulus. Structural data are shared as anonymized, raw images in the directories for single-subject raw files. Subjects from sub-01 to sub-09 performed the execution experiment, whereas subjects from sub-10 to sub-18 performed the imagery experiment, and subjects from sub-19 to sub-27 performed the observation experiment. Detailed information about the data analysis procedure and subjects are given in the README, `dataset_description.json` and subjects’ `.tsv` files, respectively.

Availability of source code and requirements

For each set of data released with this manuscript, we included dedicated Matlab codes to access and, when possible, plot data. Please refer to the ReadMe of each folder and to the specific files for a detailed description. All the codes were tested with Matlab version R2019b (The Mathworks Inc., Natick, MA, USA).

Abbr.	Definition
EMG	Electro-MyoGraphy
EEG	Electro-EncephaloGraphy
ECG	Electro-CardioGraphy
sEMG	Surface Electro-MyoGraphy
fMRI	Functional Magnetic Resonance Imaging
VPIT	Virtual Peg Insertion Test
IMUs	Inertial Measurement Units
HCGSN	HydroCel Geodesic Sensor Net
PIB	Polygraph Input Box
FD	Framewise Displacement
FWHM	Full Width at Half Maximum
SNR	Signal to Noise Ratio
DoF	Degree of Freedom
IQMs	Image Quality Metrics
R-PCA	Repeated-Principal Component Analysis
fPCA	functional Principal Component Analysis
mV	milliVolt
BIDS	Brain Imaging Data Structure

Table 7. List of abbreviations.

Availability of supporting data and materials

All the data associated to this manuscript are available in the Harvard Dataverse repository [56].

Declarations

List of abbreviations

This section collects a list of all the abbreviations employed in this manuscript, reported in Tab. 7.

Ethical Approval (optional)

All the experiments conducted to build this collection of data were approved by local ethical committees. Please refer to Tab. 1 for additional information on the approving institution and protocol number.

Consent for publication

All subject gave their written informed consent for publication. All the experiments were performed in accordance with the Declaration of Helsinki, and in observation of the "Guideline for good clinical practice E6(R1)International Council for Harmonization of Technical Requirements for Pharmaceuticals for Human Use (ICH)."

Competing Interests

The authors declare no competing financial interests.

Funding

This project has received funding from the European Union's Horizon 2020 research and innovation programme under grant agreement No. 688857 (SoftPro).

Author's Contributions

All the authors contributed to the design of the experimental protocol and to the development of the different setups. GA, FB, VC, MB and GV performed the experiments at UP. RG, CK, OL performed the experiments at ETHZ. TH, JK (TUM) performed the experiments in Leibniz Universität Hannover, Germany. GH, AL, ER performed the experiment at IMT. JH and AS performed the experiments at UZH. EJ and AO performed the experiments at MHH. GA, MB prepared the first version of the manuscript. All the authors participated to the preparation and the revision of the manuscript in its present shape.

Contributing to this work

Given the international effort provided to prepare this manuscript, and the firm belief that sharing and reusing human data is of paramount importance for the research community in multiple fields, such as Neuroscience, Motion Control, Robotics, Rehabilitation, and Clinical Practice, the Authors are willing to continue nourishing *U-Limb* with additional data, when available. Under these regards, other research groups are warmly invited to contribute to *U-Limb* with data on the human control of limbs, with specific focus to the upper extremities in both healthy and pathological conditions. The latter can refer to any pathological condition that induce a sensory-motor impairment in the upper limb (not only stroke, but also traumatic brain injury, spinal cord injury, injuries to motoneurons, multiple sclerosis, cerebral palsy, Guillain-Barre syndrome, essential tremor, Parkinson's disease, Autosomal Recessive Spastic Ataxia of Charlevoix-Saguenay, etc.), which may be investigated through different acquisitions modalities, such as kinematics, EMG, EEG, fMRI and others. To participate, please contact the Corresponding Author. New data will be associated either to a completely new submission or to an "Update" on this Data Note, for submission to GigaScience's sister journal, GigaByte.

Acknowledgements

Not applicable.

References

1. Huang Y, Bianchi M, Liarokapis M, Sun Y. Recent data sets on object manipulation: A survey. Big data 2016;4(4):197–216.
2. Jarque-Bou NJ, Scano A, Atzori M, Müller H. Kinematic synergies of hand grasps: a comprehensive study on a large publicly available dataset. Journal of neuroengineering and rehabilitation 2019;16(1):63.
3. Santuz A, Ekizos A, Janshen L, Mersmann F, Bohm S, Baltzopoulos V, et al. Modular control of human movement during running: an open access data set. Frontiers in physiology 2018;9:1509.
4. Scano A, Chiavenna A, Molinari Tosatti L, Müller H, Atzori M. Muscle synergy analysis of a hand-grasp dataset: a limited subset of motor modules may underlie a large variety of grasps. Frontiers in neurorobotics 2018;12:57.
5. Saudabayev A, Rysbek Z, Khassenova R, Varol HA. Human grasping database for activities of daily living with depth, color and kinematic data streams. Scientific data 2018;5:180101.
6. Schreiber C, Moissenet F. A multimodal dataset of human gait at different walking speeds established on injury-free adult participants. Scientific data 2019;6(1):1–7.
7. Matran-Fernandez A, Martínez IJR, Poli R, Cipriani C, Citi L. SEEDS, simultaneous recordings of high-density EMG

and finger joint angles during multiple hand movements. Scientific data 2019;6(1):1–10.
8. Jarque-Bou NJ, Atzori M, Müller H. A large calibrated database of hand movements and grasps kinematics. Scientific data 2020;7(1):1–10.
9. Roda-Sales A, Vergara M, Sancho-Bru JL, Gracia-Ibáñez V, Jarque-Bou NJ. Human hand kinematic data during feeding and cooking tasks. Scientific data 2019;6(1):1–10.
10. Jarque-Bou NJ, Vergara M, Sancho-Bru JL, Gracia-Ibáñez V, Roda-Sales A. A calibrated database of kinematics and EMG of the forearm and hand during activities of daily living. Scientific data 2019;6(1):1–11.
11. Atzori M, Gijsberts A, Castellini C, Caputo B, Hager AGM, Elsig S, et al. Electromyography data for non-invasive naturally-controlled robotic hand prostheses. Scientific data 2014;1(1):1–13.
12. Mandery C, Terlemez Ö, Do M, Vahrenkamp N, Asfour T. The KIT whole-body human motion database. In: 2015 International Conference on Advanced Robotics (ICAR) IEEE; 2015. p. 329–336.
13. Atzori M, Müller H. The Ninapro database: a resource for sEMG naturally controlled robotic hand prosthetics. In: 2015 37th Annual International Conference of the IEEE Engineering in Medicine and Biology Society (EMBC) IEEE; 2015. p. 7151–7154.
14. Atzori M, Gijsberts A, Heynen S, Hager AGM, Deriaz O, Van Der Smagt P, et al. Building the Ninapro database: A resource for the biorobotics community. In: 2012 4th IEEE RAS & EMBS International Conference on Biomedical Robotics and Biomechatronics (BioRob) IEEE; 2012. p. 1258–1265.
15. Dolatabadi E, Zhi YX, Ye B, Coahran M, Lupinacci G, Mihailidis A, et al. The toronto rehab stroke pose dataset to detect compensation during stroke rehabilitation therapy. In: Proceedings of the 11th EAI International Conference on Pervasive Computing Technologies for Healthcare; 2017. p. 375–381.
16. Santello M, Bianchi M, Gabiccini M, Ricciardi E, Salvietti G, Prattichizzo D, et al. Hand synergies: Integration of robotics and neuroscience for understanding the control of biological and artificial hands. Physics of life reviews 2016;17:1–23.
17. Averta G, Della Santina C, Battaglia E, Felici F, Bianchi M, Bicchi A. Unvealing the principal modes of human upper limb movements through functional analysis. Frontiers in Robotics and AI 2017;4:37.
18. Averta G, Angelini F, Bicchi A, Valenza G, Bianchi M. On the Role of Postural Synergies for Grasp Force Generation and Upper Limb Motion Control. In: International Conference on NeuroRehabilitation Springer; 2018. p. 344–348.
19. Averta G, Valenza G, Catrambone V, Barontini F, Scilingo EP, Bicchi A, et al. On the time-invariance properties of upper limb synergies. IEEE Transactions on Neural Systems and Rehabilitation Engineering 2019;27(7):1397–1406.
20. Schwarz A, Averta G, Veerbeek JM, Luft AR, Held JPO, Valenza G, et al. A functional analysis-based approach to quantify upper limb impairment level in chronic stroke patients: a pilot study. In: 2019 41st Annual International Conference of the IEEE Engineering in Medicine and Biology Society (EMBC); 2019. p. 4198–4204.
21. Hermens HJ, Freriks B, Disselhorst-Klug C, Rau G. Development of recommendations for SEMG sensors and sensor placement procedures. Journal of electromyography and Kinesiology 2000;10(5):361–374.
22. Catrambone V, Greco A, Averta G, Bianchi M, Vanello N, Bicchi A, et al. EEG processing to discriminate transitive-intransitive motor imagery tasks: Preliminary evidences using support vector machines. In: 2018 40th Annual International Conference of the IEEE Engineering in Medicine and Biology Society (EMBC) IEEE; 2018. p. 231–234.
23. Catrambone V, Greco A, Averta G, Bianchi M, Bicchi A, Scilingo EP, et al. EEG complexity maps to characterise brain dynamics during upper limb motor imagery. In: 2018 40th Annual International Conference of the IEEE Engineering in Medicine and Biology Society (EMBC) IEEE; 2018. p. 3060–3063.
24. Catrambone V, Greco A, Averta G, Bianchi M, Valenza G, Scilingo EP. Predicting object-mediated gestures from brain activity: an EEG study on gender differences. IEEE Transactions on Neural Systems and Rehabilitation Engineering 2019;27(3):411–418.
25. Catrambone V, Averta G, Bianchi M, Valenza G. Toward brain-heart computer interfaces: a study on the classification of upper limb movements using multisystem directional estimates. Journal of Neural Engineering 2021;.
26. Klem G, et al. The ten twenty electrode system: international federation of societies for electroencephalography and clinical neurophysiology. American J EEG Technol 1961;1(1):13–19.
27. Kanzler CM, Rinderknecht MD, Schwarz A, Lamers I, Gagnon C, Held JP, et al. A data-driven framework for selecting and validating digital health metrics: use-case in neurological sensorimotor impairments. NPJ Digital Medicine 2020;3(1):1–17.
28. Bischoff-Grethe A, Ozyurt IB, Busa E, Quinn BT, Fennema-Notestine C, Clark CP, et al. A technique for the deidentification of structural brain MR images. Human brain mapping 2007;28(9):892–903.
29. Cox RW. AFNI: software for analysis and visualization of functional magnetic resonance neuroimages. Computers and Biomedical research 1996;29(3):162–173.
30. Jenkinson M, Beckmann CF, Behrens TE, Woolrich MW, Smith SM. Fsl. Neuroimage 2012;62(2):782–790.
31. Power JD, Barnes KA, Snyder AZ, Schlaggar BL, Petersen SE. Spurious but systematic correlations in functional connectivity MRI networks arise from subject motion. Neuroimage 2012;59(3):2142–2154.
32. Hu T, Kuehn J, Haddadin S. Identification of Human Shoulder-Arm Kinematic and Muscular Synergies During Daily-Life Manipulation Tasks. In: 2018 7th IEEE International Conference on Biomedical Robotics and Biomechatronics (Biorob) IEEE; 2018. p. 1011–1018.
33. Merriaux P, Dupuis Y, Boutteau R, Vasseur P, Savatier X. A study of vicon system positioning performance. Sensors 2017;17(7):1591.
34. Stegeman D, Hermens H. Standards for surface electromyography: The European project Surface EMG for non-invasive assessment of muscles (SENIAM) 2007;.
35. Hu T, Kühn J, Haddadin S. Forward and inverse dynamics modeling of human shoulder-arm musculoskeletal system with scapulothoracic constraint. Computer methods in biomechanics and biomedical engineering 2020;23(11):785–803.
36. Sinderby C, Lindstrom L, Grassino A. Automatic assessment of electromyogram quality. Journal of Applied Physiology 1995;79(5):1803–1815.
37. Fluet MC, Lambercy O, Gassert R. Upper limb assessment using a virtual peg insertion test. In: 2011 ieee international conference on rehabilitation robotics IEEE; 2011. p. 1–6.
38. Esteban O, Birman D, Schaer M, Koyejo OO, Poldrack RA, Gorgolewski KJ. MRIQC: Advancing the automatic prediction of image quality in MRI from unseen sites. PloS one 2017;12(9):e0184661.
39. Gorgolewski KJ, Alfaro-Almagro F, Auer T, Bellec P, Capotă

M, Chakravarty MM, et al. BIDS apps: Improving ease of use, accessibility, and reproducibility of neuroimaging data analysis methods. PLoS computational biology 2017;13(3):e1005209.
40. Averta G, Della Santina C, Valenza G, Bicchi A, Bianchi M. Exploiting upper-limb functional principal components for human-like motion generation of anthropomorphic robots. Journal of NeuroEngineering and Rehabilitation 2020;17:1–15.
41. Averta G, Caporale D, Della Santina C, Bicchi A, Bianchi M. A technical framework for human-like motion generation with autonomous anthropomorphic redundant manipulators. In: Robotics and Automation (ICRA), 2020 IEEE International Conference On. IEEE; 2020. .
42. Fink J. Anthropomorphism and human likeness in the design of robots and human-robot interaction. In: International Conference on Social Robotics Springer; 2012. p. 199–208.
43. ;.
44. Cubelli R, Marchetti C, Boscolo G, Della Sala S. Cognition in action: Testing a model of limb apraxia. Brain and cognition 2000;44(2):144–165.
45. Handjaras G, Bernardi G, Benuzzi F, Nichelli PF, Pietrini P, Ricciardi E. A topographical organization for action representation in the human brain. Human brain mapping 2015;36(10):3832–3844.
46. De Renzi E, Lucchelli F. Ideational apraxia. Brain 1988;111(5):1173–1185.
47. Ochipa C, Rothi LG, Heilman KM. Ideational apraxia: A deficit in tool selection and use. Annals of Neurology: Official Journal of the American Neurological Association and the Child Neurology Society 1989;25(2):190–193.
48. Stamenova V, Roy EA, Black SE. Associations and dissociations of transitive and intransitive gestures in left and right hemisphere stroke patients. Brain and cognition 2010;72(3):483–490.
49. Cutkosky MR, et al. On grasp choice, grasp models, and the design of hands for manufacturing tasks. IEEE Transactions on robotics and automation 1989;5(3):269–279.
50. Feix T, Romero J, Schmiedmayer HB, Dollar AM, Kragic D. The grasp taxonomy of human grasp types. IEEE Transactions on human-machine systems 2015;46(1):66–77.
51. Kanzler CM, Schwarz A, Held JP, Luft AR, Gassert R, Lambercy O. Technology-aided assessment of functionally relevant sensorimotor impairments in arm and hand of post-stroke individuals. bioRxiv 2020;.
52. Kanzler CM, Gomez SM, Rinderknecht MD, Gassert R, Lambercy O. Influence of arm weight support on a robotic assessment of upper limb function. In: 2018 7th IEEE International Conference on Biomedical Robotics and Biomechatronics (Biorob) IEEE; 2018. p. 1–6.
53. Kanzler CM, Catalano MG, Piazza C, Bicchi A, Gassert R, Lambercy O. An objective functional evaluation of myoelectrically-controlled hand prostheses: a pilot study using the Virtual Peg Insertion Test. In: 2019 IEEE 16th International Conference on Rehabilitation Robotics (ICORR) IEEE; 2019. p. 392–397.
54. Leo A, Handjaras G, Bianchi M, Marino H, Gabiccini M, Guidi A, et al. A synergy-based hand control is encoded in human motor cortical areas. Elife 2016;5:e13420.
55. Gorgolewski KJ, Auer T, Calhoun VD, Craddock RC, Das S, Duff EP, et al. The brain imaging data structure, a format for organizing and describing outputs of neuroimaging experiments. Scientific data 2016;3(1):1–9.
56. Averta G, Barontini F, Catrambone V, Haddadin S, Handjaras G, Held JPO, et al., U-Limb. Harvard Dataverse; 2020. https://doi.org/10.7910/DVN/FU3QZ9.

Click here to access/download;Manuscript;cover_letter.pdf

UNIVERSITÀ DI PISA

Research Center E. Piaggio
University of Pisa

Prof. Dr. Scott Edmunds
Editor-in-Chief
Gigascience
26th of April 2021

Submission of the revised version for the manuscript "U-Limb: A multi-modal, multi-center database on arm motion control in healthy and post-stroke conditions" to Gigascience

Dear Prof Scott Edmunds,

Please find attached for your consideration the revised version of our manuscript (GIGA-D-21-00005) entitled "U-Limb: A multi-modal, multi-center database on arm motion control in healthy and post-stroke conditions" for possible publication in Gigascience.

We would like to take this opportunity to thank the Editor and the Reviewers for their valuable effort in reviewing this paper and for the very useful comments and suggestions. We take all of them seriously into account to produce what we hope may be a better work. A detailed list of the most salient changes and answers to reviewers' comments follows (in blue). All the modified and new parts of the current version of the manuscript are highlighted in red.

Yours sincerely,

Dr. Giuseppe Averta, on behalf of all the authors

Click here to access/download;Manuscript;Answers_to_reviewers.pdf

Answers to Referees

Editor

[E-Q1] Your manuscript "U-Limb: A multi-modal, multi-center database on arm motion control in healthy and post-stroke conditions" (GIGA-D-21-00005) has been assessed by our reviewers. It is of interest, but the reviewers have raised a number of points which we believe would improve the manuscript and may allow a revised version to be published in GigaScience.

[E-A1] We thank the Editor for the encouraging words. We are grateful to the Editorial Staff and the Reviewers for their useful and constructive comments. We took them carefully into account to produce what we believe is a better manuscript. In the following, we report a detailed answer to each comment together with a description of the corrective actions we implemented. The new text in the current version of the paper is highlighted in red.

[E-Q2] Regarding minor point #2 of reviewer 1 (the links to the data) I checked again and the links are working for me in principle, it's probably just a formatting thing in the document.

[E-A2] We thank the Editor for double-checking it. In hindsight we acknowledge that there was a typo in the previous version of the manuscript. We fixed it and the link correctly works now.

Referee #1

[R1-Q1] Open access experimental data is key to foster the progress in many scientific domains, such as neuroscience, rehabilitation, physiotherapy, robotics, prosthetics and biomechanics. Upper limb as a whole is a complex musculoskeletal system and there is a need of neural and local control of muscle-skeletal data of healthy and pathological subjects in order of understanding better the theoretical bases of sensory-motor control of upper limb.

Therefore, collecting and releasing an exhaustive data related to physiological and kinematic data is critical. Because of this, the current study is on a topic of relevance and general interest to the readers of the journal. However, I have several doubts about the methodology employed as well as the suitability of the data shared.

[R1-A1] We thank the Reviewer for the nice summary of our work, and for his positive words on the relevance of our work to the readers of the journal. We worked hard to address all the Reviewer's comments that we took into account to produce what we hope may be a better paper. Below we report a detailed response to each comment.

[R1-Q2] The data presented can be very useful and the amount of experimental data carried out is very considerable. However, I have three main concerns that could limit its usage: the lack of mention of some important upper limb datasets, how kinematic data is shared and the lack of more specific location of EMG sensors.

[R1-A2] We thank the Reviewer for having raised these points. Below we provide a detailed response to individually address each of these three concerns, together with a description of the corrective actions we implemented to improve the clarity and the readability of the manuscript. We hope these actions may have clarified the Reviewer's doubts.

[R1-Q3] Background. I miss some relevant datasets of the upper limb (more in particular, at hand and forearm level) in which physiological data has been shared, such as kinematics and/or EMG, focusing on activities of daily living, with standardized protocols, and sharing not only raw data but also physiological angles.

[R1-A3] We thank the Reviewer for the suggestion of including additional datasets of upper limb, considering physiological data collected in activities of daily living, which we were not aware of. In hindsight we acknowledge that this definitely helps to better clarify our contribution with respect to the related state of the art. We included the following references in the new version of the paper.

- Jarque-Bou NJ, Atzori M, Müller H. A large calibrated database of hand movements and grasps kinematics. Scientific data 2020;7(1):1–10.

- Roda-Sales A, Vergara M, Sancho-Bru JL, Gracia-Ibáñez V, Jarque-Bou NJ. Human hand kinematic data during feeding and cooking tasks. Scientific data 2019;6(1):1–10.

- Jarque-Bou NJ, Vergara M, Sancho-Bru JL, Gracia-Ibáñez V, Roda-Sales A. A calibrated database of kinematics and EMG of the forearm and hand during activities of daily living. Scientific data 2019;6(1):

- Atzori M, Gijsberts A, Castellini C, Caputo B, Hager AGM, Elsig S, et al. Electromyography data for non-invasive naturally-controlled robotic hand prostheses. Scientific data 2014;1(1):1–13

[R1-Q4] Kin data. Kinematic data is presented as raw data: 1) x-y-z position of markers or 2) IMU-based angular reconstructions with a specific format (.mvnx). Raw data is very useful for several cases, but it also difficult its use in others. Although the location of the marker is shown in Figures 1 and 2, it could be very helpful if the anatomical angles between the joints were also included in the data (raw data plus calibrated anatomical data). This fact could facilitate the use of data for people without sufficient technical knowledge to obtain the anatomical angles from markers position. Furthermore, it is known that using calibrated anatomical angles with similar protocols is very useful for comparing results between subjects, sessions, and labs, as these anatomical values are less affected by operator differences (when placing markers) or different laboratory conditions. In addition, a clearer statement about the specific kinematic data structure should be mentioned within the text.

[R1-A4] We thank the Reviewer for having raised up this crucial point. We acknowledge that the release of joint angular values could be useful for fostering data re-usage by people without sufficient technical background. At the same time, our original idea of releasing raw data was motivated by the fact that this format is independent from the specific data analysis in use (including pre-processing and calibration phases). Indeed, different procedures can be used for joint angle identification starting from motion capture data. Considering the reasons above, we opted for a trade-off solution. We clearly report in the text the references where the procedures for data identification were explained, together with pseudocode showing the implementation of the identification procedure employed for the kinematic data id H1 (new Algorithm 1 in the manuscript). This procedure can be generalized to the other datasets acquired through a motion capture system. It is worth noticing again that this is only one of the possible

procedures for angle identification and we do not want forcing other researchers to use the same procedures. However, we do believe that in this manner we provided the theoretical and technical tools for implementing a possible identification pipeline for all the kinematic data. We do really hope that this trade-off solution may accommodate the reasonable request by the Reviewer (the release of angular values) with the authors' motivations. Finally, regarding the IMU data, we would like to underline that the .mvnx format is the standard format provided by the Xsense sensing suit used for data collection. There are free libraries provided by the manufacturer that enable the interface between Xsense proprietary software and other analysis tools, such as Matlab. We included the code required to import data in Matlab together with the dataset.

[R1-Q5] EMG data. Recording data from different laboratories could lead to different muscular activity measurements, even though the same muscle appears to be recorded. Therefore, for each experiment performed, a photography o scheme indicating the position of the electrodes should be included in the data files. This is not mandatory, as I know that maybe the authors do not have that information, but all the possible information about sensors position is recommended. In the same way, some figures could be helpful to clarify these positions (as similar done with kinematic markers in figures 1 and 2). This information could be helpful to discuss future results when using the data.

[R1-A5] We thank the Reviewer for having raised this point. We agree with the fact that a picture of the experimental setup could be useful. Unfortunately, we did not collect these pictures at the time of the experiments. To increase the comprehension on sensor position we included a new figure in the manuscript where we show the positioning of EMG sensors (new fig. 4 of the manuscript, also attached below). We also provide the list of the recorded muscles that, in our opinion, is a fundamental information that the reader cannot infer from a picture of the sensing setup. We would like to underline that in all the experimental procedures we followed SENIAM guidelines for the placement of the electrodes and there was a continuous information exchange between the different research groups during data collection. We do believe that this reasonably guaranteed an uniform placement of the sensors across the different research centers.

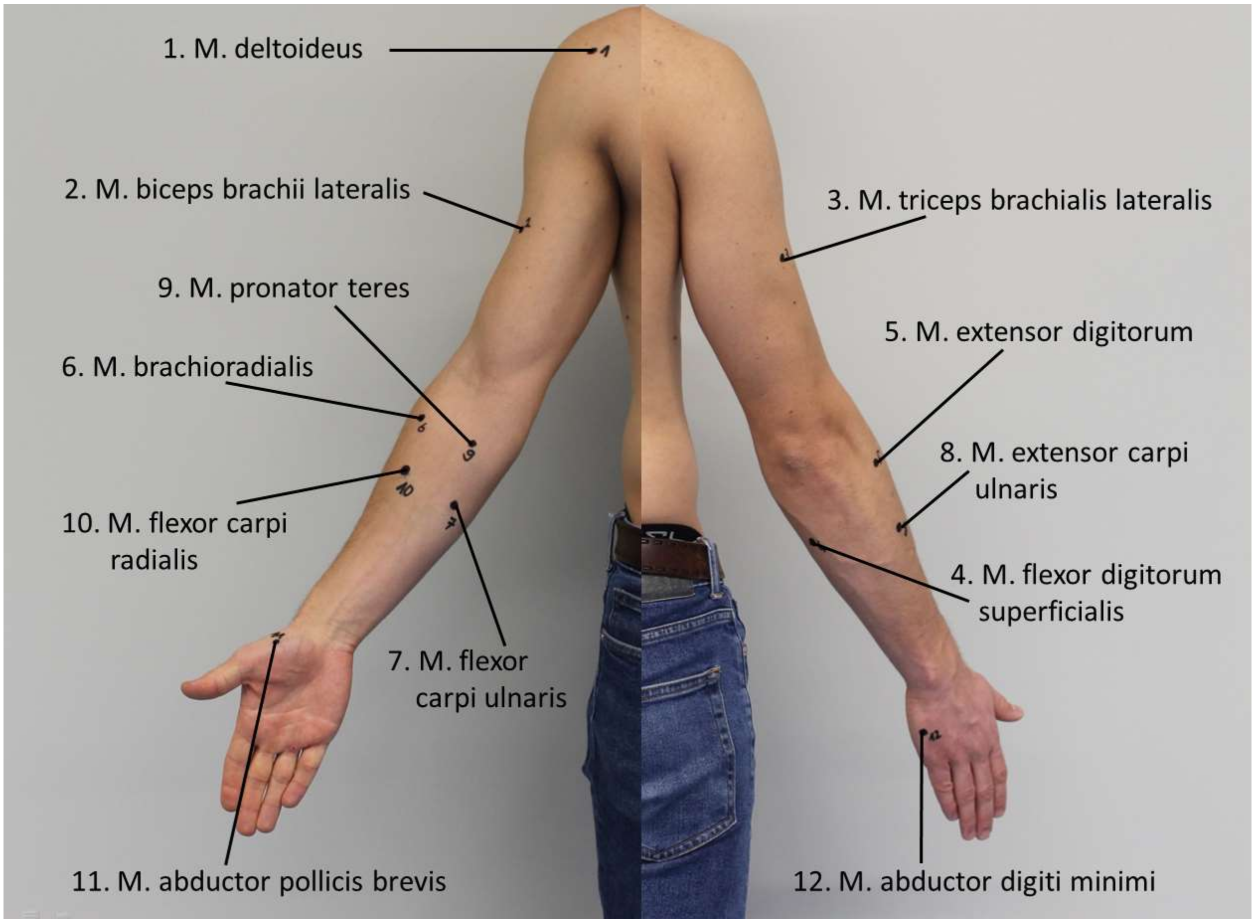

Figure 4 Placement of EMG sensors following SENIAM guidelines

[R1-Q6] Figures 1-3. Please, improve the quality of these figures. The current figures have not enough quality to be published in their present form.

[R1-A6] We thank the Reviewer for noticing this issue. We modified the three figures and improved their quality. We hope they are clearer now. Figures are also reported below for reader's convenience.

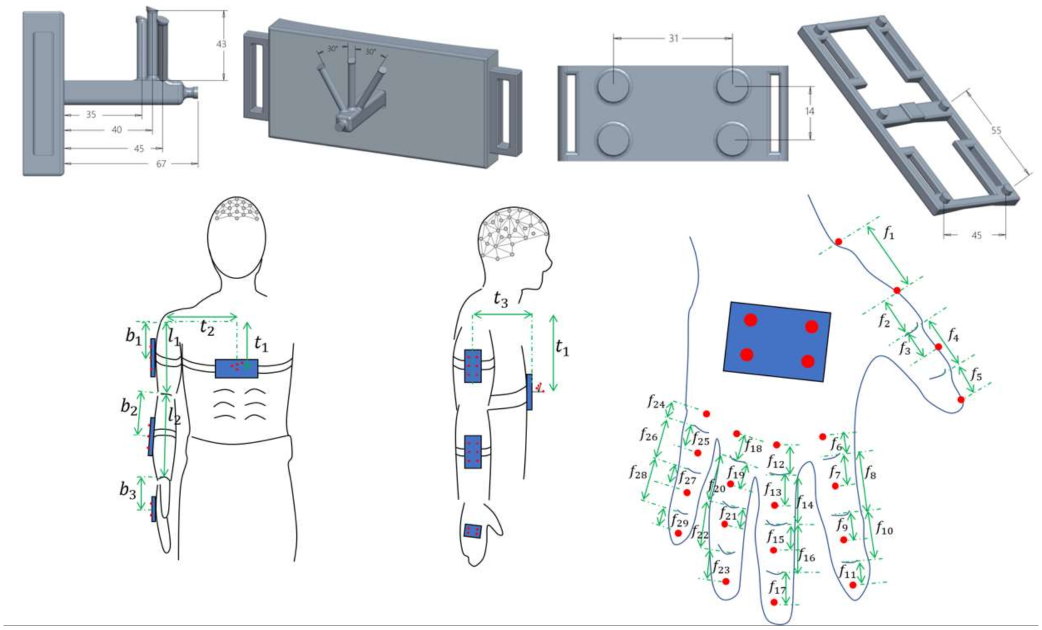

Figure 1

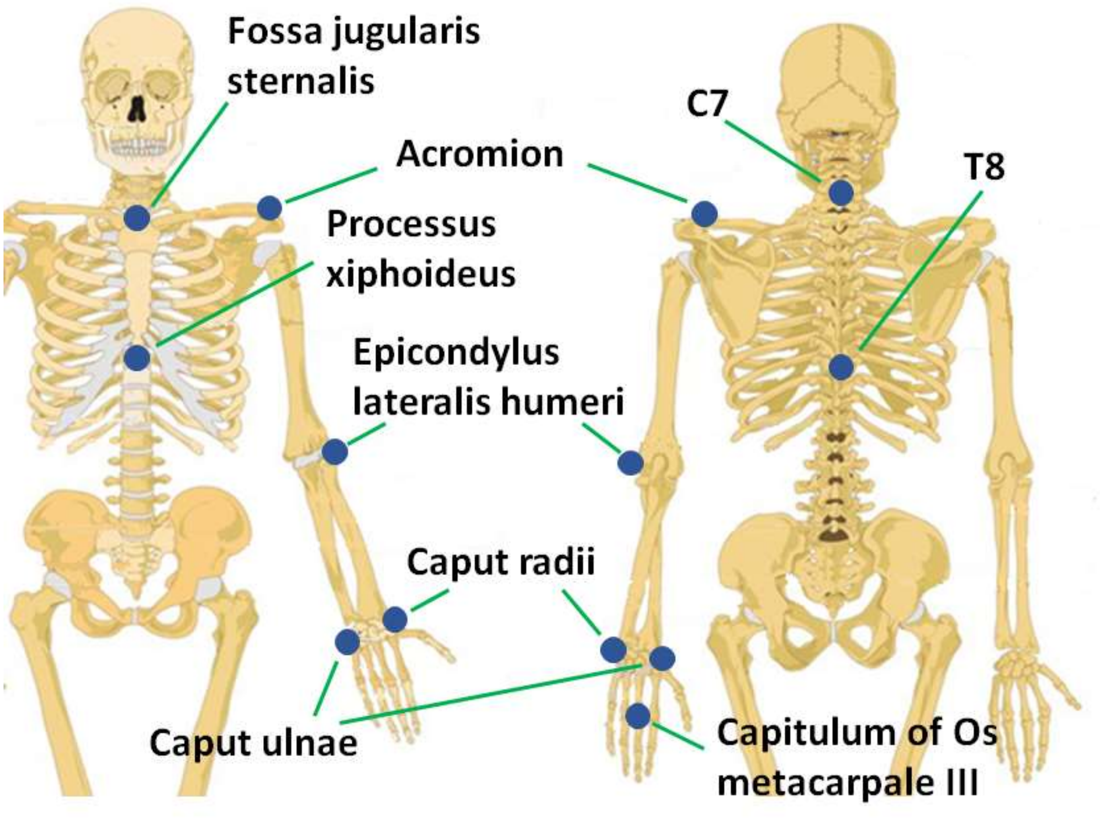

Figure 2

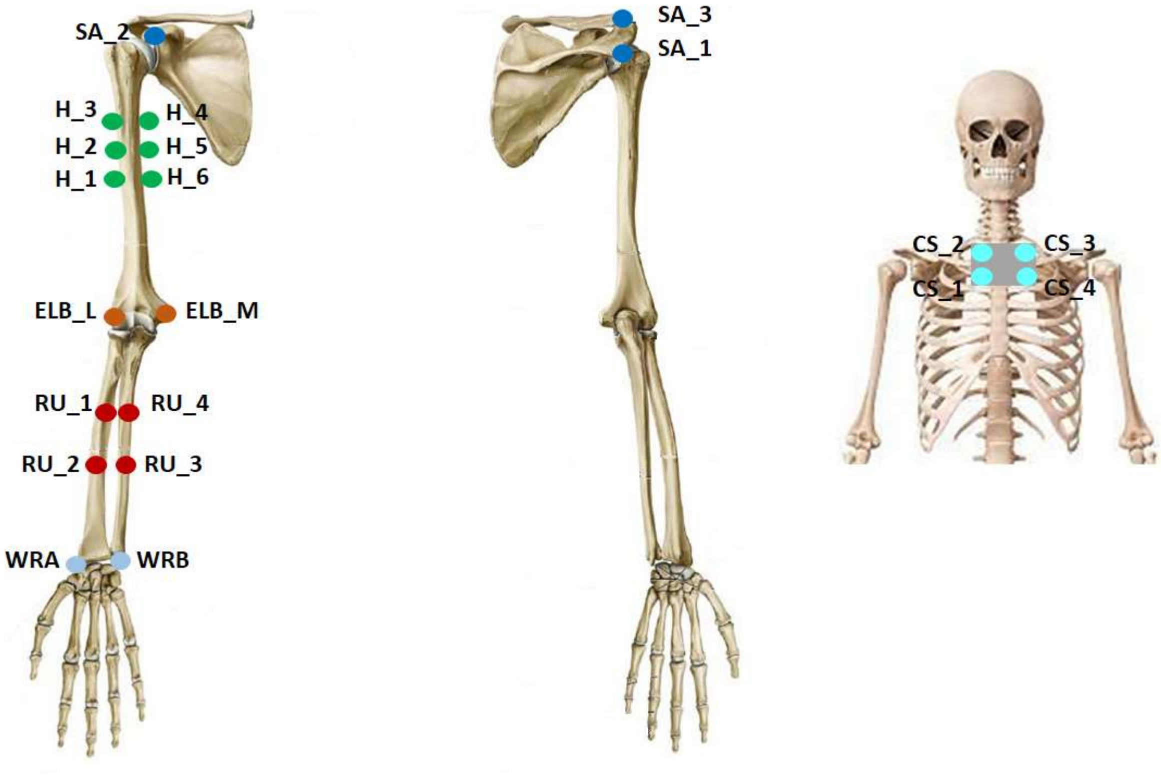

Figure 3

[R1-Q7] DOI and link from "Availability of supporting data and materials" section are not working. Please fix them.

[R1-A7] We thank the reviewer for noticing this issue. In hindsight we acknowledge that there was a typo in the previous version of the manuscript. We fixed it and the link correctly works now. We also verified all the other links provided in the paper.

Referee #2

[R2-Q1] In this manuscript, Averta and colleagues describe a dataset, consisting of healthy subjects and stroke patients performing daily activities while kinematic and physiological data were recorded. The dataset also provides fMRI data during executed, imagined and observed hand movements and end-effector kinematics during a virtual peg insertion test. The manuscript is nicely written and the data are well described. I downloaded the UP dataset and it was easy to get the EEG and kinematics data. I believe this is a great initiative, very much needed and I fully agree with the authors that this dataset will constitute a very useful resource for the neuroscience and robotics communities. I have only a few minor remarks, mainly clarifications that I hope the authors will find useful.

[R2-A1] We thank the Reviewer for the nice and accurate summary of our work. We are pleased to read that our effort was appreciated by the Reviewer. At the same time, we are grateful to the Reviewer for their useful comments that we took into account in producing what we hope may a be a better manuscript. In the following, we report a detailed answer to each comment.

[R2-Q2] There are surely many interesting questions that could be answered using the patient data. However, I think it begs the question of whether a control group can be found in the healthy subject data, which would match on basic demographics (e.g. age, sex and handedness). Maybe the authors could briefly comment on this point.

[R2-A2] We thank the Reviewer for raising this point, which is highly relevant for the future usage of our data. Indeed, as mentioned, it is important to have a control group (healthy/able-bodied/not affected) when using patient data that would ideally math based on demographics. While we did an effort to reach the same gender balance in both patient and control, it was difficult to match the age. Indeed, the healthy subjects were mainly young persons, e.g. university students enrolled in the study, while the age of stroke patients was considerably higher. For the sake of completeness, we report all the required information on healthy and stroke subjects for all the datasets in terms of age, sex, handedness in Table 2, which is now extended to report also info on handedness. More specifically, as reported in Table 2, for data of group α (collected by UZH) the control group is C, for data of group β (collected by MHH) the control group is B. Regarding group γ, subjects were tested on both the impaired and the unimpaired arm, and the latter set of data may serve as control. We acknowledge that this point was not clear in the paper, and we managed to mention this correspondence in the manuscript. The section that contains the details on post-stroke subjects is now modified as in the following (in red the new sentences):

> *i Group α: 20 post-stroke subjects, 5 female, age 61±10.69 years, 11 right-arm affected, recorded by UZH, subjects were tested on both arms. Note that these subjects are a subset of Group γ, and that the IDs are coherent between the two datasets. Note also that these subjects were collected with the same experimental protocol and by the same experimenters of Group C, and these may serve as control group when using data of Groupα.*
>
> *ii Groupβ: 20 post-stroke subjects, of which 6 female, age49.88±16.92 years, 12 right-arm affected, recorded by MHH, subjects were tested on the impaired arm. Note that these subjects were collected with the same experimental protocol and by the same experimenters of Group B, and these may serve as control group when using data of Groupα.*

iii Group γ: 27 post-stroke subjects, 14 female, age 59.0±10.93 years, 26 right-handed, recorded by ETHZ. Subjects were tested on both arms. Because both the unimpaired and impaired arm were tested in Group γ, we suggest the user to consider the first set of data as control group with respect to the second.

[R2-Q3] 2) I found it a bit difficult to understand whether some subjects were recorded concurrently with multiple modalities or not. I presume that all subjects in group A were recorded for all four H1, H2, H3 and H4, not that some subjects in group A performed H1, others H2, etc... This might be due to the use of the term "healthy subjects" (Hx; 0 < x < 11) for what is in my understanding recording modalities (e.g. Table 1, "Data type" section). Maybe this could be briefly clarified.

[R2-A3] As correctly pointed out by the Reviewer, subjects included in the same Group (see Tab 2) performed one single experiments, in which multiple sensing modalities were used. For example, subjects of Group A did the experiment while kinematics, EEG and ECG were recorded simultaneously. The id used in Table 1, instead, is required to provide further information regarding technical details of each sensing modality, and is extensively used in section Data Type. We clarified this aspect, as suggested by the Reviewer, in the caption of Table 1, by adding the following sentence:

Note that different id of this table may correspond to the same group of subjects. For example, subjects of Group A were a cohort of 39 healthy participants who performed one single experiment while kinematics, EEG and ECG recordings were simultaneously recorded (id H_1, H_2, H_3)

[R2-Q4] Finally, since data recorded with other protocols than softpro were included, I am curious as to whether the authors plan to consider additional contributions from other groups too. Obviously, I understand that curating such a dataset would require a significant amount of work, however if such inclusions are planned, it might be useful to explicitly state it in the manuscript.

[R2-A4] We thank the Reviewer for suggesting this interesting idea. We are more than happy to welcome additional efforts to our collection of data. To support this idea, we included a completely new section right before the references, named "Contributing to this Work", where we explain how to add further data to U-Limb. We hope this may be in line with the policy of the journal. Below we report the text of the new section:

Given the international effort provided to prepare this manuscript, and the firm belief that sharing and reusing human data is of paramount importance for the research community in multiple fields, such as Neuroscience, Motion Control, Robotics, Rehabilitation, and Clinical Practice, the Authors are willing to continue nourishing U-Limb with additional data, when available. Under these regards, other research groups are warmly invited to contribute to U-Limb with data on the human control of limbs, with specific focus to the upper extremities in both healthy and pathological conditions. The latter can refer to any pathological condition that induce a sensory-motor impairment in the upper limb (not only stroke, but also traumatic brain injury, spinal cord injury, injuries to motoneurons, multiple sclerosis, cerebral palsy, Guillain-Barre syndrome, essential tremor, Parkinson's disease, Autosomal Recessive Spastic Ataxia of Charlevoix–Saguenay, etc.), which may be investigated through different acquisitions modalities, such as kinematics, EMG, EEG, fMRI and others. To participate, please contact the Corresponding Author. New data will be associated either to a completely new Data Note or to a Commentary to Gigascience, depending on the proposed contribution.

Details:

[R2-Q5] * I would have been interested to have an expected range for the number of trials per subject in the manuscript (I only found it for the fMRI data: 100 trials).

[R2-A5] We thank the Reviewer for reporting this issue. We noticed that this information was indeed missing in the paper. We included the number of expected trials for each subject for the SoftPro protocol by adding the following sentences:

> *During the experiment, each task was repeated at least three times, resulting in a minimum number of 90 independent acquisitions for each subject.*

Regarding the VPIT protocol, instead, such information was already included in the manuscript in section "Experimental Protocols/VPIT protocol" and consists of 5 repetitions for each subject.

[R2-Q6] * I did not find information about where the ground electrode (and sometimes the reference electrode) where placed for the EEG.

[R2-A6] We are sorry for the inconvenience. We cannot provide the information required, because, as explained in the EGI producer Technical Manual: <<*There is no "ground" sensor per se. The subject is never connected to earth ground. This would make the subject vulnerable to electrical hazard, just as standing in water makes one vulnerable to electric shock. The "ground" sensor on the Net is actually an "isolated common," which means it is tied to the zero level or common of the isolated amp circuit's power supply. This supply is isolated, so it is not connected to earth, the computer, or anything else. Thus, an electrical hazard would not make a dangerous circuit with the isolated common.*>>

Regarding the reference, EGI default reference is at the vertex for reasons of symmetry. If the user prefers to change the reference, this could be performed easily via software (as done in [20,21,22,23]).

We clarified this point also in the paper (page 5), by adding:

> *The "ground" sensor on the Net is an "isolated common," which means it is tied to the zero level or common of the isolated amp circuit's power supply. A schematic representation of channels location is provided in Figure 4.*

Furthermore, we now also included a schematic representation of HydroCel Geodesic Sensor Net (HCGSN-128), which was missing in the previous version of the manuscript (new Figure 5 of the manuscript), where the reference electrode is represented.

[R2-Q7] * I think the only way to get the electrode positions is through plotting the positions from the "chanlocs" structure. Is there maybe an electrode position schema that could be included to the UP dataset?

[R2-A7] The HydroCel Geodesic Sensor Net (HCGSN-128) is a widely used sensor net, but we understand that including an electrode location scheme would help the readability of the paper and improve the clarity of the dataset description. In the revised manuscript, we now provide it as the new Figure 4.

Here we report the Figure for Reviewer's convenience.

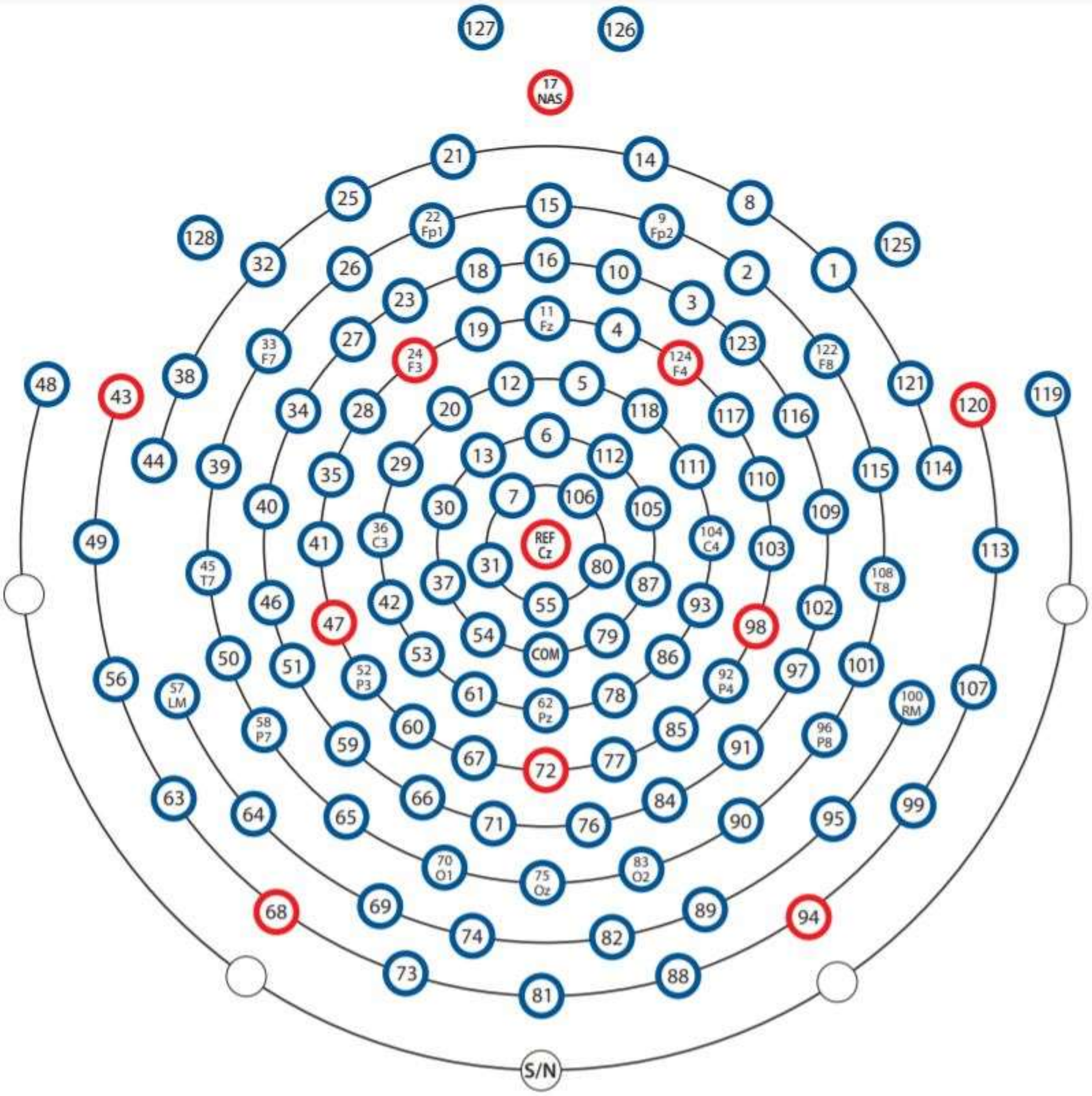

Figure 5 HydroCel Geodesic Sensor Net (HCGSN-128) scheme.

[R2-Q8] * I did not find what event the markers correspond to. I assume it is the beginning of the trial, or the movement onset, but it would be nice to have this explicitly stated, also in order to be certain of how to synchronize different recording signals (e.g. EEG and kinematics).

[R2-A8] Regarding the markers included in the EEG recordings, as correctly mentioned by the Reviewer, these correspond to the beginning of each trial. We clarified this important technical point at page 10, by adding:

> *Each acquisition is complemented with a number of markers that identify the beginning of each repetition of a single task.*

[R2-Q9] * "neural hand control" in the abstract sounded a bit weird.

[R2-A9] We edited the definition in the abstract, and this is now reported as “brain activity”. We thank the Reviewer for having raised up this point.

Manuscript

Click here to access/download;Manuscript;GigaScience_Journal_Manuscrip

GigaScience, 2017, 1–14

doi: xx.xxxx/xxxx
Manuscript in Preparation
Paper

PAPER

U-Limb: A multi-modal, multi-center database on arm motion control in healthy and post-stroke conditions

Giuseppe Averta[1,2,*], Federica Barontini[1,2], Vincenzo Catrambone[1], Sami Haddadin[3], Giacomo Handjaras[4], Jeremia P. O. Held[5], Tingli Hu[3], Eike Jakubowitz[6], Christoph M. Kanzler[7], Johannes Kühn[3], Olivier Lambercy[7], Andrea Leo[4], Alina Obermeier[6], Emiliano Ricciardi[4], Anne Schwarz[5], Gaetano Valenza[1], Antonio Bicchi[1,2] and Matteo Bianchi[1]

[1]Research Center "Enrico Piaggio" and Dipartimento di Ingegneria dell'Informazione, University of Pisa, Pisa, Italy and [2]Soft Robotics for Human Cooperation and Rehabilitation, Fondazione Istituto Italiano di Tecnologia, Genova, Italy and [3]Chair of Robotics Science and Systems Intelligence, Munich School of Robotics and Machine Intelligence, Technical University Munich (TUM), Munich, Germany and [4]MoMiLab Research Unit, IMT School for Advanced Studies Lucca, Lucca, Italy and [5]Division of Vascular Neurology and Neurorehabilitation, Department of Neurology, University of Zurich and University Hospital Zurich, Zurich, Switzerland and [6]Laboratory for Biomechanics and Biomaterials (LBB), Department of Orthopaedic Surgery, Hannover Medical School, Hannover, Germany and [7]Rehabilitation Engineering Laboratory, Institute of Robotics and Intelligent Systems, Department of Health Sciences and Technology, ETH Zurich, Switzerland.

[*]g.averta3@gmail.com

Abstract

Shading light on the neuroscientific mechanisms of human upper limb motor control, both in healthy and pathological conditions (e.g. after a stroke), can help to devise effective tools for a quantitative evaluation of the impaired conditions, and to properly inform the rehabilitative process. Furthermore, the design and control of mechatronic devices can also benefit from such neuroscientific outcomes, with important implications for assistive and rehabilitation robotics and advanced human-machine interaction. To reach these goals, we believe that an exhaustive data collection on human behavior is a mandatory step. For this reason, we release *U-Limb*, a large, multi-modal, multi-center data collection on human upper-limb movements, with the aim of fostering trans-disciplinary cross-fertilization. This collection of signals consists of data from 91 able bodied and 65 post-stroke subjects and is organized at three levels: (i) upper limb daily living activities, during which kinematic and physiological signals (electro-myography, electro-encephalography and electro-cardiography) were recorded; (ii) force-kinematic behavior during precise manipulation tasks with a haptic device; (iii) brain activity during hand control using functional magnetic resonance imaging.

Key words: Motion Control; Upper Limb; Stroke; Human Kinematics; EMG; EEG; fMRI; VPIT;

Compiled on: May 13, 2021.
Draft manuscript prepared by the author.

Key Points

- A comprehensive dataset on human upper limb during daily-living activities, which encompasses both healthy and pathological (i.e. post-stroke) conditions, to foster a quantitative evaluation of the impaired conditions, and provide guidelines to inform the rehabilitative process.
- The dataset contains multi-modal signals, which consist of kinematic-postural data; physiological recordings (electromyography, electro-encephalography and electro-cardiography); force-kinematic data during precise manipulation tasks with a haptic device; functional magnetic resonance imaging data on hand fine motor control, in imagined, performed and observed manipulation tasks. The goal is to offer a privileged point of view to unveil different yet related aspects of human upper limb motor control.
- Data were acquired at different research and clinical centers, using shared and integrated protocols, to ensure the robustness of the acquired information.

Background

An open access approach to experimental data on human sensory-motor behaviour has become extremely popular in the recent years, not only for neuroscience and clinics, but also for devising new design and control guidelines in robotics. This interest has been strengthened by the widespread adoption of deep learning techniques for analyzing human movements, which has fostered the translation of neuroscientific observations for robot control, design and planning [1]. In literature, it is possible to find a number of datasets focusing on human loco-manipulation, in which data were acquired using different acquisition modalities, ranging from RGB cameras to optical markers and electro-myographic techniques [2, 3, 4, 5, 6, 7, 8, 9, 10, 11]. Among them, it is worth mentioning the KIT Whole-Body Human Motion Database[1], a comprehensive motion capture database of whole-body human motion [12], and the NinaPro database, which consists of surface electro-myography (sEMG) data acquired from 67 intact subjects and 11 amputated subjects, who were asked to perform 50 different movements [13, 14].

Although these datasets represent an important tool for improving the knowledge on the neuroscientific aspects underpinning motor generation and control in humans, their focus was limited on specific acquisition modalities or anatomical parts. Looking at the upper limb as a whole (i.e considering the entire kinematic chain), there is poor or no evidence of databases where multi-modal and multi-center data have been collected. Furthermore, pathological conditions, such as post-stroke subject data, are rarely considered. To the best of authors' knowledge, the only example in literature is the Toronto Rehab Stroke Pose Dataset [15], which consists of upper body 3D poses recorded through Microsoft Kinect Sensors of 9 stroke patients and 10 healthy subjects performing a set of tasks using an upper limb rehabilitation robot.

In this work, we strive to release an exhaustive collection of data related to the neural and local control of upper limb muscle-skeletal system, the *U-Limb* dataset (consisting of 91 able bodied and 65 post-stroke subjects acquired), with the aim of describing upper limb motions in both healthy (i.e participants with no known history of neurological or physical issue) and pathological conditions. The two great novelties of this work are (i) multi-modality and (ii) multi-centricity, i.e. data were acquired at different research and clinical centers, using shared and integrated protocols. The choice of multi-centricity is also motivated by the need for guaranteeing the robustness of the collected data. At the same time, multi-modal acquisitions can offer a privileged point of view to unveil different yet related aspects of human upper limb motor control. For example, kinematic data can shade light on the workspace and the phenomenological characteristics of healthy movements, while offering a benchmarking to comparatively evaluate the severity of the motor impairment. Under this regard it is worth underlining that the postural data contained in the *U-Limb* dataset, which are related to daily living activities, refer to both able-bodied and stroke subjects. These subjects underwent through the same experimental protocol, which also include sEMG and electro-enecphalography (EEG) measurements, to provide information on the level of muscular tone and brain connectivity, respectively, thus offering a unique opportunity to identify quantitative tools for informing and evaluating the rehabilitative outcomes. Furthermore, these different types of information can be used to analyze whether and to which extent the abundance of healthy sensory-motor degrees of freedom of upper limb is organized in low dimensional representations, or synergies, whose study has received a lot of attention in the last decade. More specifically, the main focus of these studies has been on human hands, and it has driven important technological translational outcomes for engineering, assistive and rehabilitation robotics, and advanced human-machine interaction [16]. In parallel to daily living activities, we also report on data that target the observation of precise force-kinematic coordination in manipulation tasks with a robotic device, and functional magnetic resonance imaging (fMRI) data on hand fine motor control in imagined, performed and observed manipulation tasks. In this way, we can provide a comprehensive description of the neuroscientific aspects underpinning motion generation along the whole upper limb kinematic chain, highlighting the different aspects (kinematic, muscular, neural, dynamic) of this process.

These data were collected within the recently ended H2020 EU funded Project SoftPro (grant agreement No. 688857), whose goal was to move from the understanding of the theoretical bases of sensory-motor control of upper limb to produce a strong impact in different fields of research, clinical practice and technology. More details on data organization and collection are provided in the following sections.

Data Description

During the SoftPro Project, we collected different sets of physiological and kinematic data on human upper limb, in both healthy and pathological conditions. The latter refer to post-stroke subjects, whose clinical characteristics are reported later in the text.

Data acquisition followed three experimental protocols, i.e. the lists of tasks the subjects were asked to perform during the acquisition:

1 https://motion-database.humanoids.kit.edu/

- daily-living activities, hereinafter referred to as **SoftPro protocol**;
- hand grasping and control for the fMRI experiments, hereinafter referred to as **fMRI protocol**;
- coordination of arm and hand movements as well as grasping forces during a virtual, goal-directed object manipulation task performed with a haptic device, hereinafter referred to as **VPIT** (Virtual Peg Insertion Test) **protocol**.

The details of each protocol are reported in the dedicated section and subsections.

Data collection was organized to be multi-center and to encompass different acquisition and signal modalities. More specifically, the contributors to the generation of these datasets are: University of Pisa (UP), Istituto Italiano di Tecnologia (IIT), Hannover Medical School (MHH), Technical University of Munich (TUM), University of Zurich (UZH), Swiss Federal Institute of Technology in Zurich (ETHZ) and IMT School for Advanced Studies Lucca (IMT). Data types are:

i. kinematic recordings (optical marker positions or Inertial Measurement Units (IMUs) -based reconstructions of angular values through commercial sensing systems), hereinafter referred to as **KIN data**;
ii. Electro-Myo-Graphy (EMG) signals, hereinafter referred to as **EMG data**;
iii. Electro-Encephalo-Graphy (EEG) signals, hereinafter referred to as **EEG data**;
iv. Electro-Cardio-Graphy (ECG) signals, hereinafter referred to as **ECG data**;
v. functional Magnetic Imaging Resonance (fMRI), hereinafter referred to as **fMRI data**;
vi. kinematic end-effector, grasping force, and haptic interaction data from the VPIT protocol, hereinafter referred to as **VPIT data**.

The details of each experimental acquisition procedure are reported in the dedicated following section.

The information on the able bodied subjects (gender, average age, handedness) who took part at the experimental sessions are briefly summarized in the following:

i Group A: 39 healthy subjects, 17 female, age 26.6 ± 4.2 years, all right-handed, recorded by UP, subjects were tested on the right arm;
ii Group B: 20 healthy subjects, 8 female, age 46.77 ± 15.25 years, 18 right-handed, recorded by MHH, subjects were tested on their dominant hand;
iii Group C: 5 healthy subjects, 2 females, age 59.15 ± 15.85 years, recorded by UZH, subjects were tested on both arms;
iv Group D: 6 healthy male subjects, age 29.17 ± 5.91 years, all right-handed, recorded by TUM, Subjects were tested on the right arm;
v Group E: 27 healthy subjects, divided in three independent groups of nine subjects (5 female) each, all right-handed. Execution experiment: age 29 ± 3 years, imagery experiment: age 27 ± 6 years, observation experiment: age 25 ± 2 years, all recorded by IMT.

The details of the post-stroke subjects involved in the experiments are reported as it follows:

i Group α: 20 post-stroke subjects, 5 female, age 61±10.69 years, 11 right-arm affected, recorded by UZH, subjects were tested on both arms. Note that these subjects are a subset of Group γ, and that the IDs are coherent between the two datasets. Note also that these subjects were collected with the same experimental protocol and by the same experimenter of Group C, and these may serve as control group when using data of Group α.
ii Group β: 20 post-stroke subjects, of which 6 female, age 49.88 ± 16.92 years, 12 right-arm affected, recorded by MHH, subjects were tested on the impaired arm. Note that these subjects were collected with the same experimental protocol and by the same experimenter of Group B, and these may serve as control group when using data of Group α.
iii Group γ: 27 post-stroke subjects, 14 female, age 59.0 ± 10.93 years, 26 right-handed, recorded by ETHZ. Subjects were tested on both arms. Because both the unimpaired and impaired arm were tested in Group γ, we suggest the user to consider the first set of data as control group with respect to the second.

Figure 1. Anatomical placement of active markers, and details on the marker support used for the experiments at UP. Numerical values on the dimensions of marker support are in [mm].

An overview of all the data reported in this publication is finally provided in Tab. 1, where we also indicate the contributor and the details of the Ethical Committee that gave the approval to acquire and share these data in an anonymous form. Additional details on the cohort of subjects enrolled for each group is collected in Tab. 2. All subjects gave written informed consent before the start of the experiment.

Details on the pathology level of stroke subjects

Specific details on the level of impairment for subjects of groups α, β and γ are reported in the accompanying files included in the corresponding dataset directory.

Data Type

KIN data

Kinematic data encompass both (i) optical marker positions and (ii) IMU-based angular reconstructions during the implementation of the Softpro protocol. Regarding (i), we collected different sets of data containing the measurements of 3D optical marker coordinates related to the upper limb movements. Although different across labs, the placement of markers is always sufficient - with a certain redundancy - to enable the estimation of upper-limb movements and the identification of a minimum set of Degrees of Freedom (DoFs), relying on a shared kinematic model (see for example [17]). In the following we provide additional details for each dataset, referring to the id reported in Tab 1.

H_1 Subjects of group A were enrolled in this study. 20 active markers were placed on rigid supports fastened on arm links. In particular, 4 markers were placed on the chest, 6 markers on the arm, 6 markers on the forearm and 4 markers on the hand dorsum. In addition, 20 active markers were also placed on the subject's fingers to track hand movements. Marker 3D position was recorded via a PhaseSpace motion capture system. Marker locations and id are reported in Fig. 1. Subject-specific physical distances between groups of markers and kinematic landmarks are provided in the data folder. See also [17, 18, 19] for further details.

H_4 Subjects of group B were involved in this study. Arm movements were tracked trough 21 passive markers fastened on arm skin. Marker trajectories were captured using an optical infrared motion capturing system based on 12 MX-cameras controlled by Nexus software, Version 1.8.5 (Vicon Motion System Ltd., Oxford, UK) at a sampling rate of 200

id	Type	Group	Protocol	Contributor	Ethical Committee Approval Number
H_1	KIN	A	SoftPro	UP	1072-2016
H_2	EEG	A	SoftPro	UP	1072-2016
H_3	ECG	A	SoftPro	UP	1072-2016
H_4	KIN	B	SoftPro	MHH	3364-2016
H_5	EMG	B	SoftPro	MHH	3364-2016
H_6	KIN	C	SoftPro	UZH	BASEC-ID 2016-02075
H_7	KIN	D	SoftPro	TUM	EV LUH 05/2016
H_8	EMG	D	SoftPro	TUM	EV LUH 05/2016
H_9	EEG	D	SoftPro	TUM	EV LUH 05/2016
H_{10}	fMRI	E	fMRI	IMT	1616/2003(amended), 1072/2016
P_1	KIN	α	SoftPro	UZH	BASEC-ID 2016-02075
P_2	KIN	β	SoftPro	MHH	3364-2016
P_3	EMG	β	SoftPro	MHH	3364-2016
P_4	VPIT	γ	VPIT	ETHZ	EKNZ-2016-02075, EK2017-00398

Table 1. Details on the groups of subjects enrolled in the studies. Ids H_x refer to healthy subjects, while ids P_x to pathological subjects. All the experiments were carried out in accordance with principles of the Declaration of Helsinki, and approved by the Local Institutional Research Ethical Committees. All subjects gave written informed consent before the start of the experiment. Experiments performed at UP were approved by the Ethics Committee of the Area Vasta Nord-Ovest Toscana, Italy; experiments performed at MHH were approved by the Ethics Committee of Hannover Medical School; experiments performed at UZH were approved by the Cantonal Ethics Committee Northwest and Central Switzerland; experiments performed by TUM were approved by the Ethics Committee of Leibniz Universität Hannover, Germany; experiments performed at IMT were approved by the Ethics Committee of the Area Vasta Nord-Ovest Toscana, Italy; experiments performed at ETHZ were approved by the Ethics Committee of ETH Zurich. Note that different id of this table may correspond to the same group of subjects. For example, subjects of Group A were a cohort of 39 healthy participants who performed one single experiment while kinematics, EEG and ECG recordings were simultaneously recorded (ids H_1, H_2, H_3).

Group	Contrib.	Subj. no	Age	M/F	Handedness R/L	Average FMA score
A	UP	39	26.6 ± 4.2	22/17	39/0	N/A
B	MHH	20	46.8 ± 15.3	12/8	18/2	N/A
C	UZH	5	59.2 ± 15.9	3/2	5/0	N/A
D	TUM	6	29.2 ± 6	6/0	6/0	N/A
E	IMT	27	27.0 ± 2	22/5	27/0	N/A
α	UZH	20	61.0 ± 10.7	15/5	19/1	17.8 ± 2.1 (up to 66)
β	MHH	20	49.9 ± 16.9	14/5	12/8	17.8 ± 2.1 (up to 20)
γ	ETHZ	27	59.0 ± 10.9	13/14	26/1	46.6 ± 9.3 (up to 66)

Table 2. Details on the different populations included in this manuscript. For each group of subjects (for details on the modalities please refer to Tab. 1), we report here the Contributor, the number of subjects, their average Age, the gender balance, the Handedness (right vs. left handed)and the average stroke severity in terms of FMA score.

Hz. The marker placement and their IDs are given in figure 2.

H_6 Subjects of group C were involved in this study. The data were recorded with a full-body worn IMU-based system sensor suit (Awinda, Xsens technologies B.V., Enschede, The Netherlands). The system consists of 17 inertial measurement units (IMUs) placed symmetrically on predefined body positions and fixed with Velcro straps and a size-fitting T-Shirt. The IMUs provide 3D angular velocity using rate gyroscopes, 3D acceleration using accelerometers, 3D earth magnetic field using magnetometers, as well as atmospheric pressure using the barometer in an operating frequency 2405 – 1475 MHz. Then, proprietary software was used to reconstruct the time-varying angular deviation (roll-pitch-yaw) between subsequent IMUs. For additional details please refer to the user's manual, which can be found at the following link[2].

H_7 Subjects of group D were involved in this study. Upper-body and shoulder-arm movements were tracked using 9 passive markers, recorded using a Vicon MXT10s (Vicon Motion Systems Ltd, UK, 500 Hz) system with 8 cameras. Please refer to Fig. 2 for details of marker placement.

2 https://www.xsens.com/hubfs/Downloads/usermanual/MVN_User_Manual.pdf

Figure 2. Anatomical landmarks (blue spheres) that define the marker placement for the experiments performed at TUM (H_7). C7 and T8 refer to the vertebrae numbering, the 7th cervical and the 8th thoracical respectively.

Figure 3. Markers' placement used during the experiments performed at MHH (H_4).

To enable the analysis of the effects of stroke conditions in upper limb kinematics (i.e. movements) we recorded the motion of subjects in pathological conditions. More specifically:

P_1 Subjects of group α were enrolled in this study. Arm movements were recorded using the Xsens MVN Awinda system (same setup of H_6). This consists of 17 IMU sensors, placed on the body limbs and trunk, and of a software tool that allows data collection with a frequency of 60 Hz and reconstruct the joint angular values in time, starting from acceleration signals. Part of these data have been used in [20], to which the reader can refer for further details.

P_2 Subjects of group β were involved in this study. Arm movements were tracked trough 21 passive markers fastened on arm skin. Marker placement and data acquisition were the same used for group H2 (see fig. 3).

Figure 4. Placement of EMG sensors following SENIAM guidelines

Figure 5. A schematic representation of HydroCel Geodesic Sensor Net (HCGSN-128), channels location.

Electrode No.	Muscle
1	M. Deltoideus pars clavicularis (DC)
2	M. Biceps brachii (BB)
3	M. Triceps brachii (TB)
4	M. Flexor digitorum superficialis (FDS)
5	M. Extensor digitorum (ED)
6	M. Brachioradialis (BR)
7	M. Flexor carpi ulnaris (FCU)
8	M. Extensor carpi ulnaris (ECU)
9	M. Pronator teres (PT)
10	M. Flexor carpi radialis (FCR)
11	M. Abductor pollicis brevis (APB)
12	M. Abductor digiti minimi (ADM)

Table 3. List of 12 muscles recorded during the experiments at MHH

EMG Data

Muscular data were recorded during the experiments id H_5, H_8 and P_3. More specifically:

H_5 Subjects of Group B were enrolled in this study. A wireless surface EMG (sEMG) system (Trigno Delsys Inc., Natick USA) was used to measure the activity of 12 upper- and forearm muscles with 2000 fps (Tab. 3, see also Fig. 4). Mini sensors were used for smaller muscles (No. 9 – 12) to reduce cross talk artifacts. The 12 bipolar electrodes were placed following the SENIAM guidelines.

H_8 Subjects of group D were involved in this study. Data were collected using a Refa system (TMSi, Netherlands) with 29 bipolar channels. The 29×2 microelectrodes were placed, following the SENIAM guidelines [21], on the muscles reported in Tab. 5 (table is reported at the end of this manuscript).

P_3 Subjects of group β were involved in this study. The experimental framework used is the same of H_5 (see Tab. 3).

EEG Data

Cortical activity was recorded during the experiments id H_2 and H_9. More specifically:

H_2 Subjects of group A were enrolled in this study. Continuous EEG was recorded using a 128-channel Geodesic high-density EEG System (Electrical Geodesics Inc., Eugene, OR, USA) through a pre-cabled HydroCel Geodesic Sensor Net (HCGSN-128), sampling rate of 500 Hz with the vertex as online reference; sensor-skin impedances were maintained below 5-10 kΩ for each sensor. The “ground” sensor on the Net is an “isolated common,” which means it is tied to the zero level or common of the isolated amp circuit's power supply. A schematic representation of channels location is provided in Figure 5. These data were used for the analyses reported in [22, 23, 24, 25], to which the reader is invited to refer for further technical details.

H_9 Subjects of group D were involved in this study. An actiCHamp active EEG electrode net of 32 unipolar channels (Brain Products GmbH, Germany) – which corresponds to the 10-20 system [26] – was used at 10 kHz to record brain activity.

ECG Data

Heart electrical activity was recorded during the experiment id H_3. More specifically:

H_3 Subjects of group A were enrolled in this study. Continuous ECG was recorded using the Polygraph Input Box (PIB), the EGI's physiological measurement Geodesic System (Electrical Geodesics Inc., Eugene, OR, USA). It allows the simultaneous measurement of peripheral nervous system activity and EEG, indeed the acquisition was performed together with experiment ids H_2 and H_1. The PIB includes a bipolar channel inputs for the measurement of ECG. The input box accommodates the most common sensor connector (the 1.5 mm female safety connector) that is used in both clinical and research settings. Signals were acquired with a sampling rate of 500 Hz, applying two standard ECG sensors, the first to the lower left ribcage and the second to the upper right collarbone/clavicle, accordingly to the constructor design.

VPIT Data

Kinematic and haptic interaction data were transferred through a FireWire connection from the end-effector to a personal computer. Grasping force data were recorded through a NI (National Instruments, Austin, USA) Data Acquisition Card. The virtual reality environment of the VPIT was implemented in C++ and OpenGL. All data were sampled at 1 kHz. Missing data segments, which occurred due to a delayed communication of the C++ software, of at least 50 samples were linearly interpolated. Further, the sensor readings were low-pass filtered with a zero-phase Butterworth filter of second order and 10 Hz cut-off frequency. As the VPIT comprises multiple movement phases with different characteristics, a temporal segmentation of the continuous data streams is required to select specific parts of the movements that are relevant to describe impairments in the targeted sensorimotor functions. In more detail, the *transport* (ballistic movement after picking up a peg) and *return* (ballistic movement after releasing a peg in a hole) phases focus especially on the gross movements of the task. The start and end of these phases were identified by the moment the cursor velocity increased above and dropped below 5% of peak velocity, respectively. To quantify fine target adjustments when reaching for a target or hole, the data was segmented into the *peg approach* and *hole approach* phases. Lastly, the grasping force data was additionally divided into the *force buildup* and *force release* phases. These periods were detected by first identifying the largest maximum/minimum in the force rate profile and subsequently quantifying when the force rate dropped below and raise above 10% of the maximum/minimum force rate. More details about the data processing can be found in previous work [27].

FMRI

All fMRI data were acquired using a Philips Ingenia 3-Tesla scanner, with a 12-channel head phased array coil. Data consisted of anatomical and functional images. For anatomical images, a MP-RAGE sequence was acquired, with TR = 7 ms, TE = 3.17 ms, Flip Angle = 9°, Field of View = 224x224 mm, 156 sagittal slices, voxel size = 1x1x1 mm. To acquire functional images, a Gradient-Echo EPI sequence was used, with TR = 200 ms, TE= 30 ms Flip Angle = 75°, SENSE acceleration, factor = 2.5, Field of View = 256x256 mm, 38 interleaved axial slices, acquisition voxel size = 3x3x3 mm. Images were reconstructed with a 128x128 matrix, reconstructed voxel size was 2x2x3 mm. The top-to-bottom extent along the Z-axis was 114 mm; this ensured total brain coverage, excluding part of the cerebellum. Functional runs comprised four additional

dummy volumes, discarded by the scanner and not transferred.

Structural images were anonymized with mri_deface [28], in order to remove any anatomical detail that can allow subjects' identification. For functional MRI, the initial stages of preprocessing and the estimation of single-subject BOLD responses were performed using AFNI [29] and FSL 5.01 [30]. First, all fMRI data underwent removal of signal spikes, temporal realignment of slices, rigid-body registration to the mean image of the first run and estimation of the six motion parameters. Motion spikes were then estimated as time-points exceeding 0.5 mm of Framewise Displacement (FD) [31]; iterative spatial smoothing up to 4 mm Full Width at Half Maximum (FWHM) was subsequently performed, and the signal of each run was expressed as a percentage of the mean. Afterwards, stimulus-evoked fMRI responses were estimated for each task using a General Linear Model: the onsets of the five repetitions of each stimulus were entered into the model as regressors of interest, and the six motion parameters plus the raw value of the FD metric and polynomial trends up to the fourth order were used as regressors of no interest. The five repetitions of each stimulus were combined; for the execution and imagery experiments, we modeled the entire stimulation period (0-16 seconds) with nine tent functions peaking at 2.5 seconds. The average t-score maps from the fifth, sixth and seventh functions, that covered activity from two to six seconds after movement onset, were used as estimates of movement-relaed BOLD activity. A standard block function, convolved with the hemodynamic response, was used for the observation experiment; the modeled function started with the presentation of the video clip, and lasted one second. To avoid that baseline fMRI activity could reflect the two-alternatives task, this was modeled with a 2 seconds-long block function and the estimated BOLD responses were discarded. T-score maps from the tent functions (for the execution and imagery experiments) and from the block functions relative to the movie clip (for the observation experiment) were selected for data sharing.

Experimental setup differences among research centers

All the data acquisitions were preformed according to an integrated set of protocols. For what concerns the SoftPro protocol, the different research centers shared the same list of actions. However, specific cases required some adaptation of the general framework. Differences with respect to the general setup are reported in this section.

- Experiments of Group D were carried out inside an electromagnetically isolated chamber. For this reason, subjects were not able to execute task 22 (tennis smash) of the Softpro protocol. This task was replaced with the following one: *Reach and grasp a smartphone, unlock the screen, dial a number, and put it back to the initial position.* See also [32] for additional details.

Analyses & Technical Validation

Kinematic data

Quality of kinematic data has been tested through the evaluation of Signal to Noise Ratio (SNR).

ID H_1

Data of these experiments were collected using the PhaseSpace motion capture system, a commercial device that tracks precise motion data with sub-millimeter resolution (the amount of static marker jitter is less than 0.5 mm, usually 0.1 mm). 10 stereo-cameras were placed around the subject so to fully cover the scene (360 deg). The system was fully calibrated before the acquisition of each subject, following the standard procedure described by the manufacturer. Markers ID are automatically associated by the proprietary software tool. For these data, we quantified the SNR by selecting the three seconds of rest before the execution of each task to estimate measurement noise, and a sample of three seconds of signal during the execution of the task itself (vectors of same length). We used for this analysis one marker placed on the hand dorsum, i.e. the worst case scenario because of the reduced distance between markers. Then, from the x,y,z vectors of markers' trajectories we calculated the norm and removed the mean. From signal and noise vectors, SNR was calculated through the Matlab `snr` routine. We randomly selected 20 trials from the dataset and quantified the SNR for each sample. We obtained a median value equal to 37.54, interquartile range 4.56. These data were used for kinematic reconstructions that were employed for the principal component analysis and functional principal component analysis, which outcomes are discussed in [19] and [17], respectively. The reader can refer to those works for an example on how to pre-process and analyze the data. We also report a pseudocode (see Alg. 1) of the motion identification procedure employed in [17] to calculate joint angular values from readings of the motion capture system. This should serve as an example of data analysis that can be tailored on different acquisition systems.

ID H_4

Data of these experiments were collected through the Vicon motion capture system, a commercial device that ensure sub-millimeter errors in static conditions (see [33]). 12 cameras were used to record the scene from multiple perspectives. Marker labelling and trajectory reconstruction were performed through the proprietary software Nexus v1.8.5. For these data, SNR was quantified following the same procedure of H_1. From a random selection of 20 trials, we obtained a median value equal to 44.12, interquartile range 3.09.

ID H_6

Data of these experiments were collected through a IMU-based sensor suit, a commercial device by Xsens technologies B.V., Enschede, The Netherlands. The producer declares an accuracy in angles estimation of 0.2 deg for roll/pitch and 0.5 deg for heading angles in static conditions. These values are increased to the value of 1 deg in dynamic conditions. The whole acquisition system was properly calibrated, following the manufacturer's guidelines, before the acquisition of each subject. For these data, we quantified SNR following the same procedure used with the previous cases. SNR was evaluated on the norm of roll/pitch/yaw angles of the arm w.r.t. the chest (shoulder DoFs). Our analysis on a random selection of 20 trials reported a median value equal to 40.73, interquartile range 5.87.

ID H_7

Data of these experiments were collected through a Vicon motion capture system, similar to the one used in H_4. As previously stated, this system ensure sub-millimeter errors in static conditions (see [33]). Also in this case we quantified the SNR of data associated to the 3D position of markers placed on the hand dorsum. Our analysis on a random selection of 20 trials resulted in a median value equal to 45.0, interquartile range 6.48.

EMG data

All the experiments which involved the recording of EMG data were performed by expert experimenters who followed the SENIAM guidelines for skin preparation and electrodes placements [34]. This represents a gold standard in EMG signals

Algorithm 1 Pseudocode for the motion identification procedure used in [17]

```
procedure Setup
    X ← Load Markers data                              ▷ Load 3D position of markers
    Mod ← Load Kinematic model                         ▷ e.g. following Denavit–Hartenberg parametrization
    FK ← ForwardKinematics(Mod)                        ▷ define a model-based map from joints angular values to 3D markers positions
    Set KinPars IG                                     ▷ Initial Guess of Mod parameters
    Set KinPars LB                                     ▷ Lower Bound of Mod parameters
    Set KinPars UB                                     ▷ Upper Bound of Mod parameters

procedure Model Calibration
    X_r ← Rotate(X)                                    ▷ Rotate X w.r.t reference markers on the chest
    OptPars ← InteriorPoint(X, FK, IG, LB, UB)         ▷ Find optimal kinematic parameters using Interior Point method

procedure Motion Identification
    Set KalmanPars                                     ▷ Set parameters for Extended Kalman Filter (EKF)
    i = 1
    N ← Size(X)                                        ▷ Number of samples in X
    do
        X_i ← Select(X_r,i)                            ▷ Pick i-th sample from X
        q_i ← EKF(X_i, OptPars, FK, KalmanPars)        ▷ Identify joint angular values with EKF
        Q ← Append(q_i)                                ▷ Build a dataset of joint angular values
        i++
    while i ≠ N
```

recordings and treatment, which guarantees the highest data quality. Before the placement of EMG sensors, the corresponding skin areas were cleaned through abrasive and conductive cleaning pastes (skin impedance was controlled < 30kΩ). Before each acquisition, the recorded data were carefully visually checked on-line by an expert experimenter, and sensor locations were adjusted if necessary. Part of these data were successfully used for the identification of task-dependent muscle synergies in [32] and for the validation of a human shoulder-arm musculoskeletal dynamic model in [35], to which the interested reader is referred for an example on how to pre-process and analyze the data. It is worth mentioning that in literature EMG data typically undergo through a number of pre-processing steps to increase the quality of the collected signal and make it usable for further analyses. Since in this publication we are releasing raw data, it is difficult to find references for quantitative SNR calculated on raw data. To evaluate the SNR on the raw data released with this publication, we first performed a high-pass filtering on each bipolar channel (4th order Butterworth filter, cut-off frequency equal to 10Hz) to remove baseline shifts. Then, we calculated the SNR for each sample and for each bipolar channel. The estimation of the SNR is based on [36], a Matlab implementation can be found at the link below [3]. This evaluation of the SNR defines the noise as an unidentifiable high frequency component concentrated on the upper 20% of the frequency range (ensuring all frequencies are above 500 Hz). The module of the noise is then estimated as the average of all the power densities in the upper 20% frequency range. Then, the SNR is estimated as the ratio between the sum of all the power densities and the noise. In the data released with this publication, the median value of the SNR is always higher than 10^2.

EEG

EEG data presented were already successfully exploited in different works and from different perspectives [22, 23, 24, 25]. As well known, many different pre-processing pipelines were presented in literature to properly analyse EEG signals, they can vary according to the specific further analyses that are intended to be performed on the dataset. For this reason, in [22, 23, 24, 25] different processing steps were applied to remove artifacts and prepare the data for further analyses. A detailed description of the processing steps that have been implemented is thereby provided.

VPIT

The VPIT test is based on a CE marked haptic device, i.e. PHANTOM Omni, SensAble Technologies, Inc., USA, with a nominal position resolution higher than 450 dpi (0.055 mm). Grasping forces are recorded through three single-axis force sensors (CentoNewton 40, EPFL, Switzerland). Each sensor can accurately record force values in the range of 0 – 40 N, with a resolution of 0.05 N. The linear relationship between forces applied and voltages produced by the force sensors has been verified in [37]. To do this, the sensor was dynamically loaded and unloaded (up to 100 N/s) to three force levels (approximately 10, 20 and 30 N) against a commercial load cell (Mini 40, ATI Industrial Automation, USA) while the voltage output of the piezoresistive sensor was measured. Force data were lowpass filtered at 50 Hz and show good linearity characteristic ($V = 0.0915F + 0.726$; $R^2 = 0.9987$, where F is the applied force and V the voltage measured by the force sensor).

fMRI

Quality check of fMRI data was performed using MRIQC [38]. MRIQC is a software package, part of the bids-apps [39] that performs several processing steps in order to derive different parameters regarding image quality, such as SNR measures, motion estimates (e.g., framewise displacement) that are graphically reported as as Image Quality Metrics (IQMs) from each run in each subject. Here, we ran MRIQC on raw functional data, and plots with IQMs and mean images from single runs are included in the QC folder, which is organized in the same way as the folder containing data. Group analysis - i.e., averages and distributions of IQMs across subjects - are also included. For further information on the quality check pipeline, please refer to http://mriqc.org.

3 http://www.sce.carleton.ca/faculty/chan/matlab/matlab_library.htm

Discussion and Potential Implications

The aim of this paper is to provide an exhaustive description of the experimental protocols and acquisition techniques that finally led to the release of the dataset U-Limb. This dataset has a value *per se*, since it represents an extraordinary and unique source of information, with multiple sensory modalities that concur to shade light on different aspects underpinning the motor control of human upper limb. We do firmly believe that the release of this dataset, together with all the information needed to reproduce the experiments, can be a key component for fostering data re-usage and benchmarking, and finally advancing the research in the field of motor control. The objective is to contribute to the establishment of a trans-disciplinary community and to the definition of well-accepted guidelines for data collection. Of note, some of the data reported in this paper have already been employed and analyzed for different research purposes, and the scientific outcomes has impacted or could positively impact various fields, as already mentioned in the introductory part of the paper. In the following we report some examples of the applications of our data and discuss the trans-disciplinary impact. First and foremost, neuroscientific research can benefit from the analysis of U-Limb data. Thanks to the adoption of integrated experimental protocols, the kinematic, muscular, dynamic mechanisms, as well as the central and autonomous nervous system components related to motion execution can be investigated, at different levels of the kinematic chain (e.g. fMRI data focuses on the hand; kinematic data focuses on the whole upper limb chain). In [17] a functional Principal Component Analysis (fPCA) was applied to the kinematic data of healthy subjects, labelled as H_1, to identify the principal functional modes of human upper limb movements. Long story short, the idea was to decompose the temporal trajectories of upper limb joints in terms of a basis of functions. The results showed that a combination of few functional principal components is sufficient to reconstruct a large part of the variability of joint evolutions over time, in activities of daily living. This observation has led to the definition of a planning problem for the generation of human-like movements in robot manipulators. Briefly, the human upper limb principal motion modes computed through functional analysis were embedded in the robot trajectory optimization, thus intrinsically ensuring robot human-likeness in free motions and for obstacle avoidance [40, 41]. This point is of paramount importance in advanced human-robot interaction and assistive applications, to guarantee the safety of the human operator and the acceptability of the robotic technologies [42]. The kinematic data labelled as H_1 were also analyzed in [19], to characterize the upper limb poses at each time frame, through a technique that was named Repeated-Principal Component Analysis (R-PCA). The outcomes demonstrated that the subspace identified by the first three principal components takes into account most of the motion variability, and these results were proven to be stable over time and consistent across subjects. These finding could inform the definition of control laws for upper limb robotic devices, relying on a time-invariant low-dimensional approximation of upper limb kinematics, within the general framework of synergistic control [16]. For what concerns the kinematic data on post stroke subjects, it is worth reporting the results described in [20]. Briefly, the data labelled as P_1 were analyzed to evaluate the variations of functional principal components applied to the reconstruction of joint angle trajectories. These variations were compared between two conditions, i.e. the affected and non-affected arm, to devise a dissimilarity index for achieving an accurate and quantitative assessment of upper limb motion impairment induced by stroke. This point is extremely important to overcome the limitations of current evaluation procedures, which are mostly based on ordinal scaling, operator-dependent, and subject to floor and ceiling effects, to pave the path for a more analytical assessment that could inform the rehabilitation procedures. On the same line, the kinematic and haptic interaction data labelled as P_4 were used to devise quantitative metrics to evaluate the neurological sensorimotor impairment of upper limb kineto-dynamic behavior, in virtual peg-in-hole tasks [43]. It is worth highlighting here one of the characteristics that make the U-Limb dataset unique: i.e. the possibility to have data that cover different yet related aspects of human upper limb motor control, which allow to analyze it under different perspectives and points of view (for the examples reported before, a purely kinematic point of view for P_1, the kineto-dynamic coordination in virtual manipulation tasks for P_4). Considering the EEG data labelled as H_2, in [22, 23, 24] they were used to automatically discriminate transitive, intransitive and tool-mediated imaginary actions (as described in the *Softpro protocol*) using EEG dynamics, and relying on non linear support vector machine and Fuzzy Entropy techniques. Interestingly, in [24] different combinations of EEG-derived spatial and frequency information were investigated to find the most accurate feature vector, and gender differences between accuracies achieved with male and female data were observed. These results could open to gender-based models for the development of optimized brain machine interfaces. To conclude, U-Limb can positively impact different research fields, which encompass neuroscience and motor control; clinical assessment and rehabilitation; robotics and advanced human machine interfaces.

Methods

Experimental Protocols

SoftPro Protocol

Activities of Daily Living is a term commonly used in rehabilitation to indicate a set of everyday tasks. More recently, the usage of this class of movements has become central also in robotics to evaluate the usage of artificial systems in daily actions. The criteria for the selection of a comprehensive list of activities include (i) the specific hand grasping configuration and (ii) the direction of motion for the whole upper limb.

In the attempt of exhaustively consider all the possible combinations of (i) and (ii), we identified 30 tasks, which were divided in three different classes: intransitive, transitive and tool-mediated actions. Intransitive tasks collect movements without contact with external objects, Transitive tasks are actions which involve an external object and, finally, tool-mediated tasks are actions in which an object is used to interact with another object. This particular classification takes inspiration from the analysis presented in [44], which was proven to be reflected at the cortical level in imaging studies, e.g. [45], that show differences in cortical activation between actions belonging to the three different classes, with prefrontal and parietal regions of the left hemisphere tuned towards tool-mediated and transitive actions, whereas the right hemisphere shows a preference for meaningful, intransitive gestures. This organization has been confirmed by clinical observations as well: classic neurological studies show that, following cortical stroke, patients can develop class-specific deficits for tool-mediated actions [46, 47], and deficits for transitive or intransitive gestures have been described as a result of greater involvement of the left or right hemisphere, respectively [48].

Within a specific class, the selected actions cover different hand grasping configuration in order to span most of the postures of the main hand grasping taxonomies [49, 50]. A detailed list of actions is reported in table 4. In each row of the table, the first element reports the task number, the second

Table 4. List of action that defines the SoftPro protocol. The label *Int* stands for Intransitive tasks, the label *Tr* for Transitive tasks and the label *T-M* for Tool-Mediated tasks.

#	# [49]	Class	Description
1		Int	Ok gesture (lifting hand from the table)
2		Int	Thumb down (lifting hand from the table)
3		Int	Exultation (extending the arm up in the air with closed fist)
4		Int	Hitchhiking (extending the arm along the frontal plane, laterally, parallel to the floor, with extended elbow, closed fist, extended thumb)
5		Int	Block out sun from own face (touching the face with the palm and covering the eyes)
6		Int	Greet (with open hand, moving wrist) (3 times)
7		Int	Military salute (with lifted elbow)
8		Int	Stop gesture (extending the arm along the sagittal plane, parallel to the floor, open palm)
9		Int	Pointing (with index finger) of something straight ahead (with outstretched arm)
10		Int	Silence gesture (bringing the index finger, with the remainder of the hand closed, on the lips)
11	2	Tr	Reach and grasp a small suitcase from the handle, lift it and place it on the floor (close to own chair, along own sagittal plane)
12	3	Tr	Reach and grasp a glass, drink for 3 seconds and place it in the initial position
13	4	Tr	Reach and grasp a phone receiver, carry it to own ear for 3 seconds and place it in the initial position
14	6	Tr	Reach and grasp a book (placed overhead on a shelf), put in on the table and open it (from right side to left side)
15	8	Tr	Reach and grasp a small cup from the handle (2 fingers + thumb), drink for 3 seconds and place it in the initial position
16	11	Tr	Reach and grasp an apple, mimic biting and put it in the initial position
17	12,13	Tr	Reach and grasp a hat from its top and place it on own head
18	12	Tr	Reach and grasp a cup from its top, lift it and put it on the left side of the table
19	15	Tr	Receive a tray (straight ahead, with open hand) and put it in the middle of the table
20	16	Tr	Reach and grasp a key in a lock (vertical axis), extract it from the lock and put it on the left side of the table
21	1	T-M	Reach and grasp a bottle, pour water into a glass and put the bottle in the initial position
22	2,3,4	T-M	Reach and grasp a tennis racket (placed along own frontal plane) and play a forehand (the subject is still seated)
23	5	T-M	Reach and grasp a toothbrush, brush teeth (horizontal axis, one time left-right) and put it inside a holder (on the right side of the table)
24	6	T-M	Reach and grasp a laptop, open it (without changing its position) (4 fingers + thumb)
25	7,8,9	T-M	Reach and grasp a pen (placed on the right side of the table) and draw a vertical line on the table (from the top to the bottom)
26	7	T-M	Reach and grasp a pencil (placed along own frontal plane) (3 fingers + thumb) and put it inside a squared pencil holder (placed on the left side of the table)
27	9	T-M	Reach and grasp a tea bag in a cup (1 finger + thumb), remove it from the cup and place it on the table on the right side of the table
28	10	T-M	Reach and grasp a doorknob, turn it clockwise and counterclockwise and open the door
29	13	T-M	Reach and grasp a tennis ball (with fingertips) and place it in a basket on the floor (right)
30	14	T-M	Reach and grasp a cap (2 fingers + thumb) of a bottle (held by left hand), unscrew it and place it overhead on a shelf

links to the grasp taxonomy [49], the third indicates the class of movement and, finally, the fourth reports a brief description of the task. More details can be found in [19]. During the experiment, each task was repeated at least three times, resulting in a minimum number of 90 independent acquisitions for each subject. The temporal timeline for task execution was: 1) three seconds of rest, 2) task execution at a self-paced speed, 3) three seconds of rest. Regarding UP (IDs $\{H_1, H_2, H_3\}$) a custom C++ routine was used to associate the pressure of a keyboard key with: i) the start of 3D markers position acquisition and ii) the placement of a temporal marker in the acquisition flow of EEG/ECG recordings. The same tool was used to interrupt the task acquisition on both sides. Absolute timing is also provided in the related dataset. An analogous procedure was used at MHH (IDs $\{H_4, H_5\}$ and $\{P_2, P_3\}$), and at TUM (IDs $\{H_7, H_8, H_9\}$), where an EtherCAT system with NI 9144 (National Instruments) controlled using the Simulink tool of Matlab, was employed to send start/stop trigger signals to the acquisition systems.

VPIT protocol

The VPIT is performed using a commercial haptic end-effector (PHANTOM Omni, 3D Systems, CA, USA), a custom-made handle with force sensors (CentoNewton40, EPFL, Switzerland), and a virtual reality environment rendered on personal computer (Figure 6) [37, 51, 52, 53]. The VPIT requires the insertion of nine virtual pegs into nine virtual holes through the coordination of arm and hand movements controlling the end-effector as well as the grasping forces applied to the instrumented handle attached at the end-effector. In more detail, a virtual cursor needs to be first spatially aligned with the virtual peg. Subsequently, a peg can be picked up and transported towards a hole by applying a grasping force of at least 2 N. The peg can be released in the hole by reducing the grasping force below the threshold. The virtual pegboard is thereby physically rendered through the haptic device to ease the perception of the 3D virtual reality environment.

Figure 6. The Virtual Peg Insertion Test (VPIT) is a technology-aided assessment platform consisting of a haptic end-effector, a grasping force sensing handle, and a virtual reality environment. It allows to record kinematic and kinetic data about sensorimotor impairments in arm and hand during a functional task.

The starting position of the subjects was defined through an elbow flexion angle of $\approx$ 90 deg, a shoulder abduction angle of $\approx$ 45 deg, and a shoulder flexion angle of $\approx$ 10 deg. The protocol consists of an initial familiarization period, during which subjects were instructed to perform the task as fast and precise as possible, followed by five repetitions of the task (i.e., inserting all nine pegs five times). More details about the setup and

Electrode No.	Muscle
1	M. trapezius Pars descendens (TRPc)
2	M. trapezius Pars transversa (TRPt)
3	M. trapezius Pars ascendens (TRPa)
4	M. deltoideus Pars clavicularis (DLTc)
5	M. deltoideus Pars acromialis (DLTa)
6	M. deltoideus Pars spinalis (DLTs)
7	M. latissimus dorsi (LTDt)
8	M. pectoralis major Pars clavicularis (PMJc)
9	M. pectoralis major Pars sternocostalis (PMJs)
10	M. pectoralis major Pars abdominalis (PMJr)
11	M. biceps brachii Caput longum (BICl)
12	M. biceps brachii Caput breve (BICs)
13	M. triceps brachii Caput longum (TRClg)
14	M. triceps brachii Caput laterale (TRClt)
15	M. pronator teres (PRNT)
16	M. flexor carpi radialis et (if present) M. palmaris longus (FCR)
17	M. flexor carpi ulnaris (FCU)
18	M. flexor digitorum superficialis (FDS)
19	M. flexor pollicis longus (FPL)
20	M. extensor digitorum (EDT)
21	M. extensor digiti minimi (EDM)
22	M. extensor carpi ulnaris (ECU)
23	M. abductor pollicis longus et M. extensor pollicis brevis (APL&EPB)
24	M. brachioradialis (BRD)
25	M. extensor carpi radialis (ECR)
26	M. abductor digit minimi (ADM)
27	M. flexor pollicis brevis (FPB)
28	M. abductor pollicis brevis (APB)
29	M. interosseus dorsalis I (DI1)

Table 5. List of 29 muscles recorded during the experiments at TUM (id H_8)

the procedure can be found in previous work [37, 27].

fMRI protocol

Design for motor execution and imagery experiments were based on a previous work [54], and relied on a delayed grasping task after a visual presentation of the target objects. More specifically, in each trial, a picture of the target object was visually presented for 2 seconds, then, after a 4 seconds pause, an auditory cue prompted the actual task: subjects had to preshape the hand as if they were grasping the target object to use it (for the execution group) or imagine a preshaping movement, without moving their hand (for the imagery group). A 10 seconds interval separated two subsequent trials. Twenty different target objects were used for this study, as in (see Table 6 for a list) and, in each experiment, movements were repeated five times, for a total number of 100 trials, organized in five fMRI runs, each lasting 5′44″ minutes, including twelve seconds of rest at the beginning and at the end of each run to achieve a measure of baseline fMRI activity. The experimental paradigm for execution and imagery experiments was coded using Presentation (Neurobehavioral System, Berkeley, CA, http://www.neurobs.com), and presented with a MR-compatible monitor at the resolution of 1200x800 pixels, and a mirror mounted on the MR coil. During the observation experiment, subjects watched short videos of preshaping movements towards an object from the same set adopted in the other experiments. In each trial, the video was followed by a task that implied a judgment on the target of the preshaping gesture. To create videos, we used vectors of joint angles (according to a 24 DoFs model) corresponding to the common starting posture and to the twenty final object-specific postures, recorded in a previous study [54]. Intermediate hand configurations (i.e., posture vectors) between the initial and final postures were obtained from linear interpolation between the values of each kinematic joint angle in the initial and final hand postural configurations. The resulting 30 vectors of joint angles were plotted as 3D renderings, using Mathematica 8.0 (Wolfram Research Inc, Champaign, IL, USA), saved as png images (size: 800x600px), and converted to one second-long videos at a frame rate of 60 Hz. Five sets of 20 videos were created, showing the hand rendering as seen from five different viewpoints, obtained by changing the values of azimuth and elevation. During the fMRI experiment, subjects performed five runs, each comprising 20 trials. During each trial, the video was presented (1 second), followed by a black fixation cross at the center of the screen (7 seconds). Then, the judgment task (two-alternatives forced choice) was presented, and subjects were shown the black/white pictures of two objects (size: 250x250 px) – the target of the preshaping gesture previously shown and a randomly-chosen alternative – and asked to press the left or right key on a MR-compatible keyboard to select the actual target of the preshaping movement. After the task, the same black fixation cross was shown for 6 seconds. Each run comprised the presentation of the full set of twenty videos (20 objects), always from the same viewpoint; the five different viewpoints were presented in separate runs. Each run started and ended with 10 seconds of rest, and lasted in total 5 minutes and 40 seconds. The experimental paradigm was delivered with a MR-compatible monitor at the resolution of 1200x800 pixels, and a mirror mounted on the MR coil, using the e-Prime 2 software package (Psychology Software Tools, Pittsburgh, PA, USA). Due to hardware failure, behavioral responses from two subjects could not be recorded. For all experiments, subjects performed a familiarization run, outside the MR scanner, to ensure that they correctly understood the procedures.

Bucket	Calculator	Chalk	Cherry
Dinner plate	Espresso cup	Fishing rod	Frisbee
Hairdryer	Hammer	Telephone handset	Jar lid
Light bulb	Pc mouse	PenRope	Ice cube
Tennis racket	Toothpick	Wrench	

Table 6. List of objects used for the fMRI experiments.

Data Records

Data records published with this paper, together with the dataset summary and ReadMe, are available through the Harvard Dataverse repository. Data can be downloaded through the link in footnote [4]. The overall size is 36.2 GB. Data are organized in 6 folders, one for each research center. Within every folder, data are organized per recording modality (e.g. kinematic data, EMG and EEG). Data provided from each institution have been separately compressed in .rar format and uploaded on the repository, in such a way to enable the download of single block of data. For block heavier than 2.5 Gb, we divided the file in multiple linked parts. In these cases, to properly unpack the data the reader is required to extract the file named XXX.part1, which in turn will automatically recall the subsequent parts. Each folder contain a ReadMe file that details the folder content. In the following, we provide more detailed information for each folder.

4 https://dataverse.harvard.edu/privateurl.xhtml?token=d8ce17bf-70a5-4f43-b3e0-8b4ea333bdcf

Folder UP
In this folder, data are organized per recording modality, i.e. EEG-ECG and KIN. Each folder contains in turn 39 folder named "SXX", where XX is the subject ID. The folder Data_KIN contains the kinematic acquisition. Files are named as "SXX_Y_Z", where Y is the Task number and Z is the repetition number (e.g. *S4_23_1*). Data are collected with a sampling rate equal to 100 Hz. Each acquisition is provided in the dedicated mat file.

An identical naming has been used for the corresponding (synchronized) data of EEG-ECG signals, contained in the "Data EEG - ECG" folder. This folder contains the MFF files with the EEG and ECG data (in milliVolt(mV)). Data were gathered through EGI 128-channel system (sampling rate 500 Hz). Each acquisition is complemented with a number of markers that identify the beginning of each repetition of a single task.

Note that, for these experiments, three repetitions of the same task are provided. There are some cases in which the Z value (repetition number) is higher than 3. This can be associated to cases in which we noticed: i) errors in the task execution; ii) evident problems in the acquisition (either in kinematic data or in EEG data). In these cases, we performed additional repetitions to guarantee the minimum number of three samples of the same task. Acquisitions containing evident errors have been discarded from the dataset.

In addition, two additional folders are included, namely "read_EEG" and "read_plot_KIN ", in which we provide sample codes to access and plot the dataset. Further information about the data and the code are included in the ReadMe file.

Folder MHH
In this folder, data are organized per recording modality, i.e. EMG data and Kinematic data. These two subfolders are divided in Healthy and Stroke subjects.

Trials are named through 3 numbers (e.g. 10_8_3) where the first number (in the example 10) indicates the subject id, the second (8 in the example) indicates the task number and, finally, the third is the trial number. Stroke subjects are named with the same policy with and additional "S" at the beginning of the name.

EMG data are organized to have the rows corresponding to the time frames (sampling rate 2000 Hz), and the columns associated to the 12 measured muscles. The kinematic data files contain the position data of thorax, upper-arm and forearm markers. The table is divided in 63 columns - with 3 columns, corresponding to the x, y, z position, for each of the 21 markers; the rows, starting from the 3rd one, report the recorded marker position for each time frame (sampling rate 200 Hz). The first two rows contain respectively the marker names and the measure unit (in mm).

The file *read_emgfiles.m* is a sample Matlab code to plot the EMG data. Additional details are provided in the ReadMe file. Subject-specific information are provided in one additional file, named “Patients_details_MHH_extended.doc”. There we reported the following characteristics: ID, Age, Gender, Tested limb, Impaired limb, Dominant limb, Time since stroke, FMA-UE and MM score.

Folder UZH
In this folder, data are organized for each subject who took part to the experiment, i.e. healthy and impaired subjects. Kinematic parameters are stored in software specific XML file format (.mvnx) that enable the import to different software tools, such as MATLAB and Microsoft Excel. Each mvnx-file represents one trial execution and is named according to the subject ID (e.g. P02), task number (T01-T30), tested upper limb (R/L) and repetition (1-3). Sample Matlab codes are provided, showing how to access and plot data. More information regarding the file structure and how to plot data are provided in the ReadMe file. Subject-specific information are provided in the additional file “ParticipantCharacteristics.xlsx”. There we reported the following information: ID, Age, Gender, Impaired limb, Dominant limb, Time since stroke, FMA-UE. Note that the 20 stroke subjects enrolled in this dataset (Group α) are a subset of the 27 who performed the VPIT protocol (Group γ). Therefore, further information on subjects of this folder may also be included in the additional files included in Folder ETHZ (the ID of subjects is coherent in the two datasets).

Folder TUM
In this folder, data are organized per subjects. Each subfolder is divided per recording modality, i.e. EEG, EMG, and Kinemaitc Data (MoCap folder). Matlab files are provided to access and plot data (i.e. *plot_KIN.m*, *plot_EMG.m* and *plot_EEG.m*).

Folder ETHZ
In this folder, the provided `VPIT_Data_v3.mat` file contains processed and unprocessed VPIT data collected from 27 chronic post-stroke individuals. Data are all contained in the *VPIT_Data_v3.mat* file, in which each row corresponds to data from one specific trial. The file *header.xlsx* contains detailed meta information regarding the content of each column in the `VPIT_Data_v3.mat`. Additional information about the data, processing and procedures are provided in the ReadMe file. Subject-specific information are provided in two additional files, named “patient-information.png” and “patient-information-2.png”. There we reported the following characteristics: ID, Age, Gender, Tested limb, Impaired limb, Dominant limb, Time since stroke, FMA-UE, ARAT, NHPT, BBT, MAS, EmNSA and MOCA.

Folder IMT
In this folder, data are organized according to the Brain Imaging Data Structure (BIDS) standard [55]. Single-subject t-score maps from functional data are included in the directory for processed data (i.e., derivatives). For the execution and imagery experiments, t-scores for the fifth, sixth and seventh tent functions (i.e., with peak at 2, 4, 6 seconds after movement onset) are selected. Each stimulus is modeled using its five repetitions. The `.nii.gz` file contains the average of the three selected t-score maps. For the observation experiment, t-scores for the block functions, covering the stimulus period, are selected. Each stimulus is modeled using its five repetitions. The 2AFC task responses, though modeled, were discarded. The `.nii.gz` file contains the twenty t-score maps, one for each stimulus. Structural data are shared as anonymized, raw images in the directories for single-subject raw files. Subjects from sub-01 to sub-09 performed the execution experiment, whereas subjects from sub-10 to sub-18 performed the imagery experiment, and subjects from sub-19 to sub-27 performed the observation experiment. Detailed information about the data analysis procedure and subjects are given in the README, `dataset_description.json` and subjects’ `.tsv` files, respectively.

Availability of source code and requirements

For each set of data released with this manuscript, we included dedicated Matlab codes to access and, when possible, plot data. Please refer to the ReadMe of each folder and to the specific files for a detailed description. All the codes were tested with Matlab version R2019b (The Mathworks Inc., Natick, MA, USA).

Abbr.	Definition
EMG	Electro-MyoGraphy
EEG	Electro-EncephaloGraphy
ECG	Electro-CardioGraphy
sEMG	Surface Electro-MyoGraphy
fMRI	Functional Magnetic Resonance Imaging
VPIT	Virtual Peg Insertion Test
IMUs	Inertial Measurement Units
HCGSN	HydroCel Geodesic Sensor Net
PIB	Polygraph Input Box
FD	Framewise Displacement
FWHM	Full Width at Half Maximum
SNR	Signal to Noise Ratio
DoF	Degree of Freedom
IQMs	Image Quality Metrics
R-PCA	Repeated-Principal Component Analysis
fPCA	functional Principal Component Analysis
mV	milliVolt
BIDS	Brain Imaging Data Structure

Table 7. List of abbreviations.

Availability of supporting data and materials

All the data associated to this manuscript are available in the Harvard Dataverse repository [56]. Access to the data is granted through the following link https://dataverse.harvard.edu/privateurl.xhtml?token=d8ce17bf-70a5-4f43-b3e0-8b4ea333bdcf.

Declarations

List of abbreviations

This section collects a list of all the abbreviations employed in this manuscript, reported in Tab. 7.

Ethical Approval (optional)

All the experiments conducted to build this collection of data were approved by local ethical committees. Please refer to Tab. 1 for additional information on the approving institution and protocol number.

Consent for publication

All subject gave their written informed consent for publication. All the experiments were performed in accordance with the Declaration of Helsinki, and in observation of the "Guideline for good clinical practice E6(R1)International Council for Harmonization of Technical Requirements for Pharmaceuticals for Human Use (ICH)."

Competing Interests

The authors declare no competing financial interests.

Funding

This project has received funding from the European Union's Horizon 2020 research and innovation programme under grant agreement No. 688857 (SoftPro).

Author's Contributions

All the authors contributed to the design of the experimental protocol and to the development of the different setups. GA, FB, VC, MB and GV performed the experiments at UP. RG, CK, OL performed the experiments at ETHZ. TH, JK (TUM) performed the experiments in Leibniz Universität Hannover, Germany. GH, AL, ER performed the experiment at IMT. JH and AS performed the experiments at UZH. EJ and AO performed the experiments at MHH. GA, MB prepared the first version of the manuscript. All the authors participated to the preparation and the revision of the manuscript in its present shape.

Contributing to this work

Given the international effort provided to prepare this manuscript, and the firm belief that sharing and reusing human data is of paramount importance for the research community in multiple fields, such as Neuroscience, Motion Control, Robotics, Rehabilitation, and Clinical Practice, the Authors are willing to continue nourishing *U-Limb* with additional data, when available. Under these regards, other research groups are warmly invited to contribute to *U-Limb* with data on the human control of limbs, with specific focus to the upper extremities in both healthy and pathological conditions. The latter can refer to any pathological condition that induce a sensory-motor impairment in the upper limb (not only stroke, but also traumatic brain injury, spinal cord injury, injuries to motoneurons, multiple sclerosis, cerebral palsy, Guillain-Barre syndrome, essential tremor, Parkinson's disease, Autosomal Recessive Spastic Ataxia of Charlevoix-Saguenay, etc.), which may be investigated through different acquisitions modalities, such as kinematics, EMG, EEG, fMRI and others. To participate, please contact the Corresponding Author. New data will be associated either to a completely new submission or to an "Update" on this Data Note, for submission to GigaScience's sister journal, GigaByte.

Acknowledgements

Not applicable.

References

1. Huang Y, Bianchi M, Liarokapis M, Sun Y. Recent data sets on object manipulation: A survey. Big data 2016;4(4):197–216.
2. Jarque-Bou NJ, Scano A, Atzori M, Müller H. Kinematic synergies of hand grasps: a comprehensive study on a large publicly available dataset. Journal of neuroengineering and rehabilitation 2019;16(1):63.
3. Santuz A, Ekizos A, Janshen L, Mersmann F, Bohm S, Baltzopoulos V, et al. Modular control of human movement during running: an open access data set. Frontiers in physiology 2018;9:1509.
4. Scano A, Chiavenna A, Molinari Tosatti L, Müller H, Atzori M. Muscle synergy analysis of a hand-grasp dataset: a limited subset of motor modules may underlie a large variety of grasps. Frontiers in neurorobotics 2018;12:57.
5. Saudabayev A, Rysbek Z, Khassenova R, Varol HA. Human grasping database for activities of daily living with depth, color and kinematic data streams. Scientific data 2018;5:180101.
6. Schreiber C, Moissenet F. A multimodal dataset of human

gait at different walking speeds established on injury-free adult participants. Scientific data 2019;6(1):1–7.
7. Matran-Fernandez A, Martínez IJR, Poli R, Cipriani C, Citi L. SEEDS, simultaneous recordings of high-density EMG and finger joint angles during multiple hand movements. Scientific data 2019;6(1):1–10.
8. Jarque-Bou NJ, Atzori M, Müller H. A large calibrated database of hand movements and grasps kinematics. Scientific data 2020;7(1):1–10.
9. Roda-Sales A, Vergara M, Sancho-Bru JL, Gracia-Ibáñez V, Jarque-Bou NJ. Human hand kinematic data during feeding and cooking tasks. Scientific data 2019;6(1):1–10.
10. Jarque-Bou NJ, Vergara M, Sancho-Bru JL, Gracia-Ibáñez V, Roda-Sales A. A calibrated database of kinematics and EMG of the forearm and hand during activities of daily living. Scientific data 2019;6(1):1–11.
11. Atzori M, Gijsberts A, Castellini C, Caputo B, Hager AGM, Elsig S, et al. Electromyography data for non-invasive naturally-controlled robotic hand prostheses. Scientific data 2014;1(1):1–13.
12. Mandery C, Terlemez Ö, Do M, Vahrenkamp N, Asfour T. The KIT whole-body human motion database. In: 2015 International Conference on Advanced Robotics (ICAR) IEEE; 2015. p. 329–336.
13. Atzori M, Müller H. The Ninapro database: a resource for sEMG naturally controlled robotic hand prosthetics. In: 2015 37th Annual International Conference of the IEEE Engineering in Medicine and Biology Society (EMBC) IEEE; 2015. p. 7151–7154.
14. Atzori M, Gijsberts A, Heynen S, Hager AGM, Deriaz O, Van Der Smagt P, et al. Building the Ninapro database: A resource for the biorobotics community. In: 2012 4th IEEE RAS & EMBS International Conference on Biomedical Robotics and Biomechatronics (BioRob) IEEE; 2012. p. 1258–1265.
15. Dolatabadi E, Zhi YX, Ye B, Coahran M, Lupinacci G, Mihailidis A, et al. The toronto rehab stroke pose dataset to detect compensation during stroke rehabilitation therapy. In: Proceedings of the 11th EAI International Conference on Pervasive Computing Technologies for Healthcare; 2017. p. 375–381.
16. Santello M, Bianchi M, Gabiccini M, Ricciardi E, Salvietti G, Prattichizzo D, et al. Hand synergies: Integration of robotics and neuroscience for understanding the control of biological and artificial hands. Physics of life reviews 2016;17:1–23.
17. Averta G, Della Santina C, Battaglia E, Felici F, Bianchi M, Bicchi A. Unvealing the principal modes of human upper limb movements through functional analysis. Frontiers in Robotics and AI 2017;4:37.
18. Averta G, Angelini F, Bicchi A, Valenza G, Bianchi M. On the Role of Postural Synergies for Grasp Force Generation and Upper Limb Motion Control. In: International Conference on NeuroRehabilitation Springer; 2018. p. 344–348.
19. Averta G, Valenza G, Catrambone V, Barontini F, Scilingo EP, Bicchi A, et al. On the time-invariance properties of upper limb synergies. IEEE Transactions on Neural Systems and Rehabilitation Engineering 2019;27(7):1397–1406.
20. Schwarz A, Averta G, Veerbeek JM, Luft AR, Held JPO, Valenza G, et al. A functional analysis-based approach to quantify upper limb impairment level in chronic stroke patients: a pilot study. In: 2019 41st Annual International Conference of the IEEE Engineering in Medicine and Biology Society (EMBC); 2019. p. 4198–4204.
21. Hermens HJ, Freriks B, Disselhorst-Klug C, Rau G. Development of recommendations for SEMG sensors and sensor placement procedures. Journal of electromyography and Kinesiology 2000;10(5):361–374.
22. Catrambone V, Greco A, Averta G, Bianchi M, Vanello N, Bicchi A, et al. EEG processing to discriminate transitive-intransitive motor imagery tasks: Preliminary evidences using support vector machines. In: 2018 40th Annual International Conference of the IEEE Engineering in Medicine and Biology Society (EMBC) IEEE; 2018. p. 231–234.
23. Catrambone V, Greco A, Averta G, Bianchi M, Bicchi A, Scilingo EP, et al. EEG complexity maps to characterise brain dynamics during upper limb motor imagery. In: 2018 40th Annual International Conference of the IEEE Engineering in Medicine and Biology Society (EMBC) IEEE; 2018. p. 3060–3063.
24. Catrambone V, Greco A, Averta G, Bianchi M, Valenza G, Scilingo EP. Predicting object-mediated gestures from brain activity: an EEG study on gender differences. IEEE Transactions on Neural Systems and Rehabilitation Engineering 2019;27(3):411–418.
25. Catrambone V, Averta G, Bianchi M, Valenza G. Toward brain-heart computer interfaces: a study on the classification of upper limb movements using multisystem directional estimates. Journal of Neural Engineering 2021;.
26. Klem G, et al. The ten twenty electrode system: international federation of societies for electroencephalography and clinical neurophysiology. American J EEG Technol 1961;1(1):13–19.
27. Kanzler CM, Rinderknecht MD, Schwarz A, Lamers I, Gagnon C, Held JP, et al. A data-driven framework for selecting and validating digital health metrics: use-case in neurological sensorimotor impairments. NPJ Digital Medicine 2020;3(1):1–17.
28. Bischoff-Grethe A, Ozyurt IB, Busa E, Quinn BT, Fennema-Notestine C, Clark CP, et al. A technique for the deidentification of structural brain MR images. Human brain mapping 2007;28(9):892–903.
29. Cox RW. AFNI: software for analysis and visualization of functional magnetic resonance neuroimages. Computers and Biomedical research 1996;29(3):162–173.
30. Jenkinson M, Beckmann CF, Behrens TE, Woolrich MW, Smith SM. Fsl. Neuroimage 2012;62(2):782–790.
31. Power JD, Barnes KA, Snyder AZ, Schlaggar BL, Petersen SE. Spurious but systematic correlations in functional connectivity MRI networks arise from subject motion. Neuroimage 2012;59(3):2142–2154.
32. Hu T, Kuehn J, Haddadin S. Identification of Human Shoulder-Arm Kinematic and Muscular Synergies During Daily-Life Manipulation Tasks. In: 2018 7th IEEE International Conference on Biomedical Robotics and Biomechatronics (Biorob) IEEE; 2018. p. 1011–1018.
33. Merriaux P, Dupuis Y, Boutteau R, Vasseur P, Savatier X. A study of vicon system positioning performance. Sensors 2017;17(7):1591.
34. Stegeman D, Hermens H. Standards for surface electromyography: The European project Surface EMG for non-invasive assessment of muscles (SENIAM) 2007;.
35. Hu T, Kühn J, Haddadin S. Forward and inverse dynamics modeling of human shoulder-arm musculoskeletal system with scapulothoracic constraint. Computer methods in biomechanics and biomedical engineering 2020;23(11):785–803.
36. Sinderby C, Lindstrom L, Grassino A. Automatic assessment of electromyogram quality. Journal of Applied Physiology 1995;79(5):1803–1815.
37. Fluet MC, Lambercy O, Gassert R. Upper limb assessment using a virtual peg insertion test. In: 2011 ieee international conference on rehabilitation robotics IEEE; 2011. p. 1–6.
38. Esteban O, Birman D, Schaer M, Koyejo OO, Poldrack RA,

Gorgolewski KJ. MRIQC: Advancing the automatic prediction of image quality in MRI from unseen sites. PloS one 2017;12(9):e0184661.
39. Gorgolewski KJ, Alfaro-Almagro F, Auer T, Bellec P, Capotă M, Chakravarty MM, et al. BIDS apps: Improving ease of use, accessibility, and reproducibility of neuroimaging data analysis methods. PLoS computational biology 2017;13(3):e1005209.
40. Averta G, Della Santina C, Valenza G, Bicchi A, Bianchi M. Exploiting upper-limb functional principal components for human-like motion generation of anthropomorphic robots. Journal of NeuroEngineering and Rehabilitation 2020;17:1–15.
41. Averta G, Caporale D, Della Santina C, Bicchi A, Bianchi M. A technical framework for human-like motion generation with autonomous anthropomorphic redundant manipulators. In: Robotics and Automation (ICRA), 2020 IEEE International Conference On. IEEE; 2020. .
42. Fink J. Anthropomorphism and human likeness in the design of robots and human-robot interaction. In: International Conference on Social Robotics Springer; 2012. p. 199–208.
43. ;.
44. Cubelli R, Marchetti C, Boscolo G, Della Sala S. Cognition in action: Testing a model of limb apraxia. Brain and cognition 2000;44(2):144–165.
45. Handjaras G, Bernardi G, Benuzzi F, Nichelli PF, Pietrini P, Ricciardi E. A topographical organization for action representation in the human brain. Human brain mapping 2015;36(10):3832–3844.
46. De Renzi E, Lucchelli F. Ideational apraxia. Brain 1988;111(5):1173–1185.
47. Ochipa C, Rothi LG, Heilman KM. Ideational apraxia: A deficit in tool selection and use. Annals of Neurology: Official Journal of the American Neurological Association and the Child Neurology Society 1989;25(2):190–193.
48. Stamenova V, Roy EA, Black SE. Associations and dissociations of transitive and intransitive gestures in left and right hemisphere stroke patients. Brain and cognition 2010;72(3):483–490.
49. Cutkosky MR, et al. On grasp choice, grasp models, and the design of hands for manufacturing tasks. IEEE Transactions on robotics and automation 1989;5(3):269–279.
50. Feix T, Romero J, Schmiedmayer HB, Dollar AM, Kragic D. The grasp taxonomy of human grasp types. IEEE Transactions on human-machine systems 2015;46(1):66–77.
51. Kanzler CM, Schwarz A, Held JP, Luft AR, Gassert R, Lambercy O. Technology-aided assessment of functionally relevant sensorimotor impairments in arm and hand of post-stroke individuals. bioRxiv 2020;.
52. Kanzler CM, Gomez SM, Rinderknecht MD, Gassert R, Lambercy O. Influence of arm weight support on a robotic assessment of upper limb function. In: 2018 7th IEEE International Conference on Biomedical Robotics and Biomechatronics (Biorob) IEEE; 2018. p. 1–6.
53. Kanzler CM, Catalano MG, Piazza C, Bicchi A, Gassert R, Lambercy O. An objective functional evaluation of myoelectrically-controlled hand prostheses: a pilot study using the Virtual Peg Insertion Test. In: 2019 IEEE 16th International Conference on Rehabilitation Robotics (ICORR) IEEE; 2019. p. 392–397.
54. Leo A, Handjaras G, Bianchi M, Marino H, Gabiccini M, Guidi A, et al. A synergy-based hand control is encoded in human motor cortical areas. Elife 2016;5:e13420.
55. Gorgolewski KJ, Auer T, Calhoun VD, Craddock RC, Das S, Duff EP, et al. The brain imaging data structure, a format for organizing and describing outputs of neuroimaging experiments. Scientific data 2016;3(1):1–9.
56. Averta G, Barontini F, Catrambone V, Haddadin S, Handjaras G, Held JPO, et al., U-Limb. Harvard Dataverse; 2020. https://doi.org/10.7910/DVN/FU3QZ9.

kine_up

Click here to access/download;Figure;kine_up.png

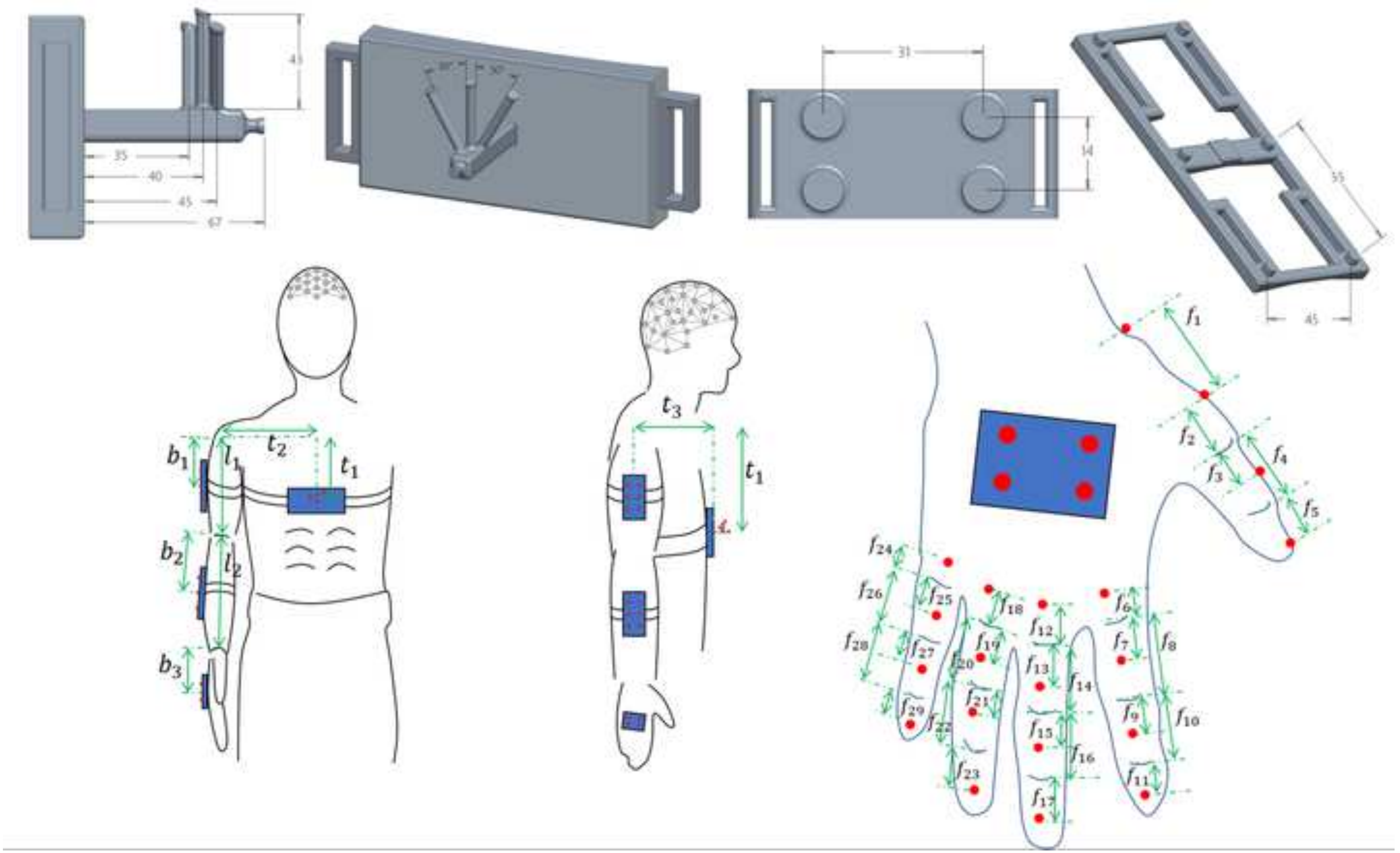

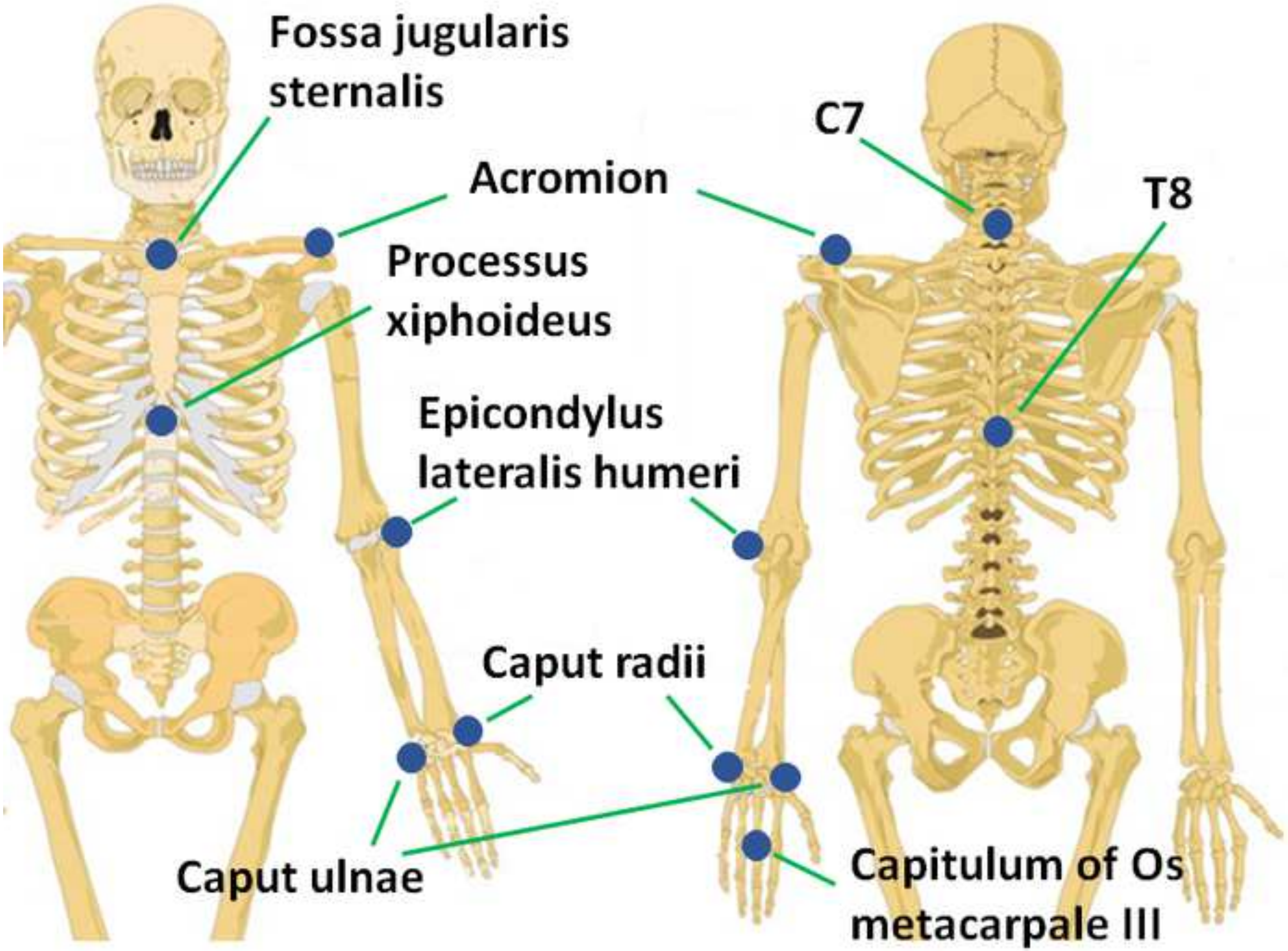
Fossa jugularis sternalis
Acromion
C7
T8
Processus xiphoideus
Epicondylus lateralis humeri
Caput radii
Caput ulnae
Capitulum of Os metacarpale III

Click here to access/download;Figure;kine_mhh.png

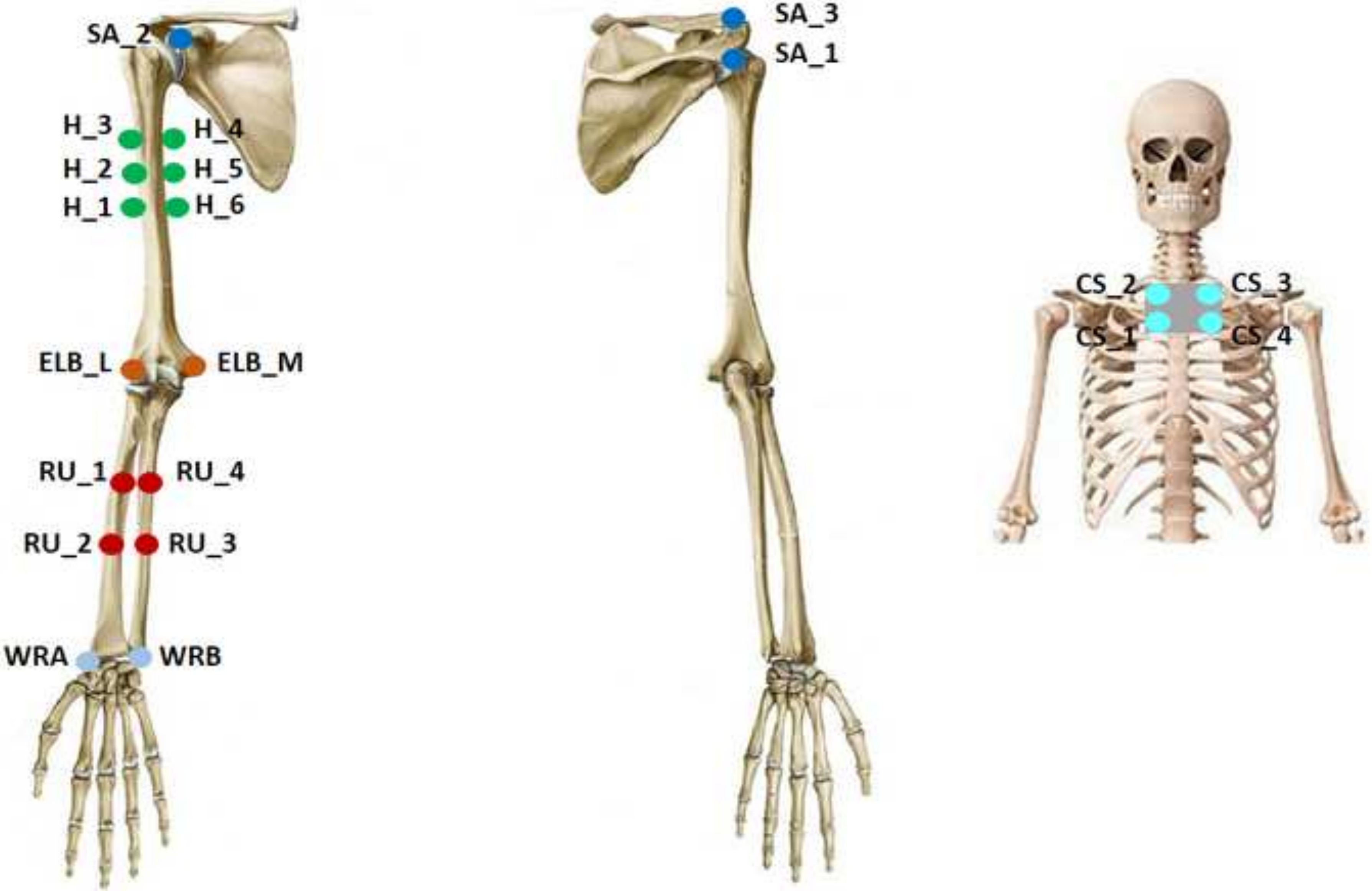

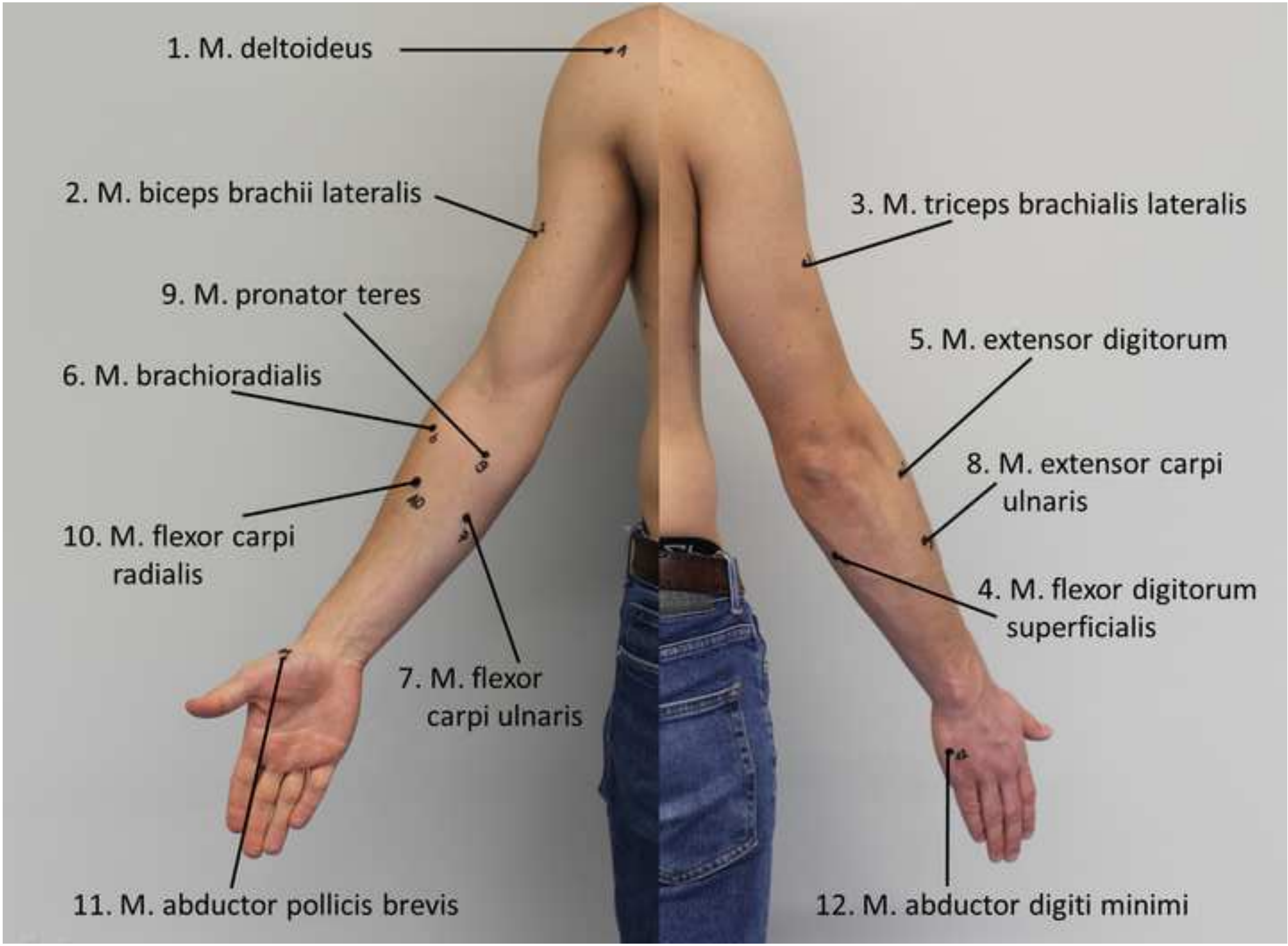
1. M. deltoideus
2. M. biceps brachii lateralis
3. M. triceps brachialis lateralis
9. M. pronator teres
5. M. extensor digitorum
6. M. brachioradialis
8. M. extensor carpi ulnaris
10. M. flexor carpi radialis
4. M. flexor digitorum superficialis
7. M. flexor carpi ulnaris
11. M. abductor pollicis brevis
12. M. abductor digiti minimi

Click here to access/download;Figure;EGI_channelMap.JPG

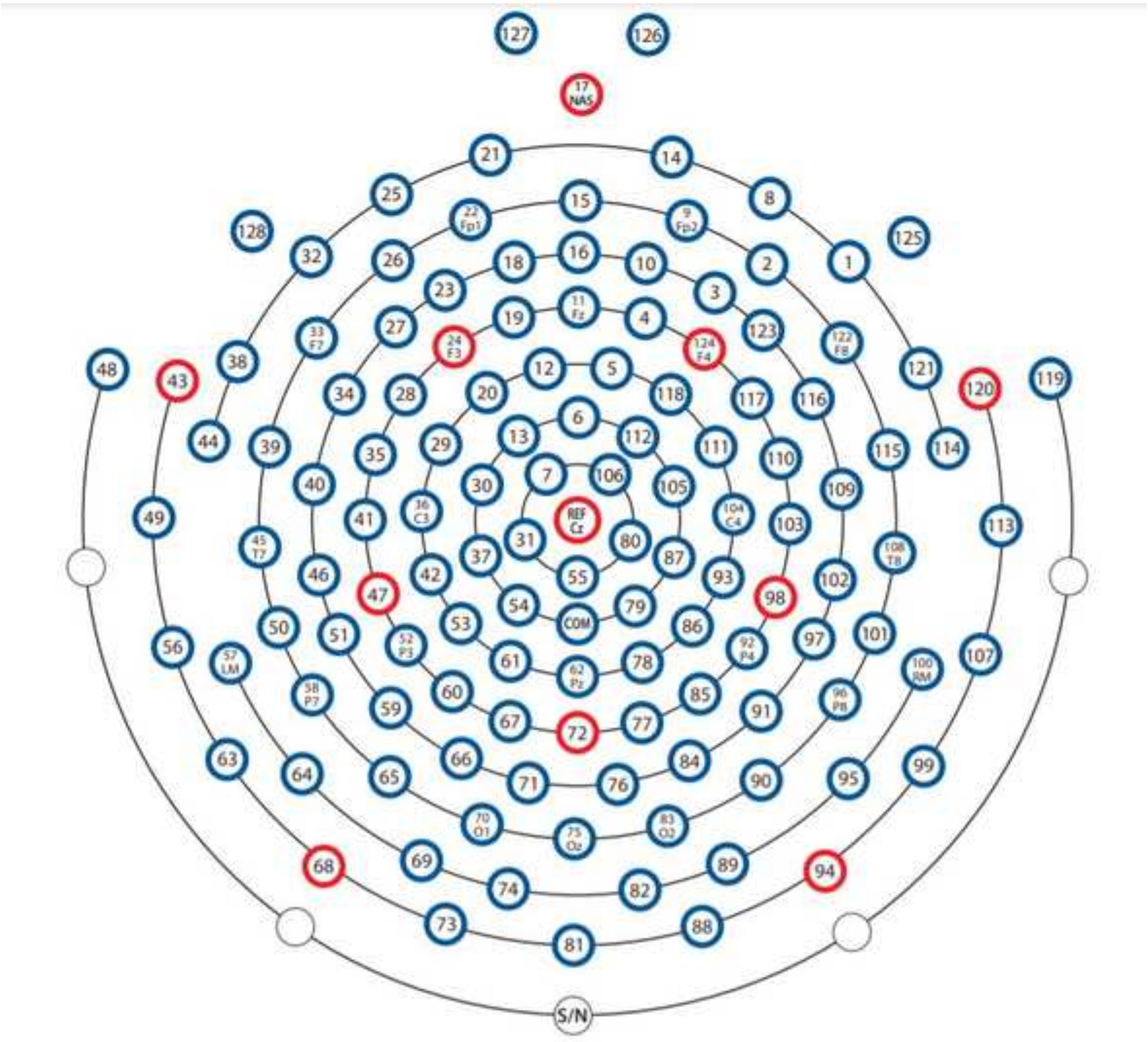

Supplement: giab043_GIGA-D-21-00005_Revision_2 [file giab043_giga-d-21-00005_revision_2.pdf]
