# Supplementary material for: U-Limb: A multi-modal, multi-center database on arm motion control in healthy and post-stroke conditions
Source: Gigascience. 2021 Jun 18;10(6):giab043. doi: 10.1093/gigascience/giab043 (PMC8212873; doi:10.1093/gigascience/giab043)
Supplement: giab043_Response_to_Reviewer_Comments_Original_Submission [file giab043_response_to_reviewer_comments_original_submission.pdf]

Author's Response To Reviewer Comments

Close

Dear Prof Scott Edmunds,

Please find attached for your consideration the revised version of our manuscript (GIGA-D-21-00005) entitled “U-Limb: A multimodal, multi-center database on arm motion control in healthy and post-stroke conditions” for possible publication in Gigascience.

We would like to take this opportunity to thank the Editor and the Reviewers for their valuable effort in reviewing this paper and for the very useful comments and suggestions. We take all of them seriously into account to produce what we hope may be a better work. A detailed list of the most salient changes and answers to reviewers’ comments is attached to the submission.

Yours sincerely
Giuseppe Averta on behalf of all the authors

Close
