# Supplementary material for: U-Limb: A multi-modal, multi-center database on arm motion control in healthy and post-stroke conditions
Source: Gigascience. 2021 Jun 18;10(6):giab043. doi: 10.1093/gigascience/giab043 (PMC8212873; doi:10.1093/gigascience/giab043)
Supplement: giab043_Response_to_Reviewer_Comments_Revision_1 [file giab043_response_to_reviewer_comments_revision_1.pdf]

Author's Response To Reviewer Comments

Close

I did all the edits required.

Close
