# Supplementary material for: U-Limb: A multi-modal, multi-center database on arm motion control in healthy and post-stroke conditions
Source: Gigascience. 2021 Jun 18;10(6):giab043. doi: 10.1093/gigascience/giab043 (PMC8212873; doi:10.1093/gigascience/giab043)
Supplement: giab043_Reviewer_1_Report_Original_Submission — NÃ©stor J. Jarque-Bou -- 1/19/2021 Reviewed [file giab043_reviewer_1_report_original_submission.pdf]

Reviewer Report

Title: U-Limb: A multi-modal, multi-center database on arm motion control in healthy and post-stroke conditions.

Version: Original Submission **Date:** 1/19/2021

Reviewer name: NÃ©stor J. Jarque-Bou

Reviewer Comments to Author:

Open access experimental data is key to foster the progress in many scientific domains, such as neuroscience, rehabilitation, physiotherapy, robotics, prosthetics and biomechanics. Upper limb as a whole is a complex musculoskeletal system and there is a need of neural and local control of muscle-skeletal data of healthy and pathological subjects in order of understanding better the theoretical bases of sensory-motor control of upper limb.

Therefore, collecting and releasing an exhaustive data related to physiological and kinematic data is critical. Because of this, the current study is on a topic of relevance and general interest to the readers of the journal. However, I have several doubts about the methodology employed as well as the suitability of the data shared.

Major comments

The data presented can be very useful and the amount of experimental data carried out is very considerable. However, I have three main concerns that could limit its usage: the lack of mention of some important upper limb datasets, how kinematic data is shared and the lack of more specific location of EMG sensors.

1. Background. I miss some relevant datasets of the upper limb (more in particular, at hand and forearm level) in which physiological data has been shared, such as kinematics and/or EMG, focusing on activities of daily living, with standardized protocols, and sharing not only raw data but also physiological angles.

2. Kin data. Kinematic data is presented as raw data: 1) x-y-z position of markers or 2) IMU-based angular reconstructions with a specific format (.mvnx). Raw data is very useful for several cases, but it also difficult its use in others. Although the location of the marker is shown in Figures 1 and 2, it could be very helpful if the anatomical angles between the joints were also included in the data (raw data plus calibrated anatomical data). This fact could facilitate the use of data for people without sufficient technical knowledge to obtain the anatomical angles from markers position. Furthermore, it is known that using calibrated anatomical angles with similar protocols is very useful for comparing results between subjects, sessions, and labs, as these anatomical values are less affected by operator differences (when placing markers) or different laboratory conditions. In addition, a clearer statement about the specific kinematic data structure should be mentioned within the text.

3. EMG data. Recording data from different laboratories could lead to different muscular activity measurements, even though the same muscle appears to be recorded. Therefore, for each experiment performed, a photography o scheme indicating the position of the electrodes should be included in the data files. This is not mandatory, as I know that maybe the authors do not have that information, but all

the possible information about sensors position is recommended. In the same way, some figures could be helpful to clarify these positions (as similar done with kinematic markers in figures 1 and 2). This information could be helpful to discuss future results when using the data.
Minor comments
1. Figures 1-3. Please, improve the quality of these figures. The current figures have not enough quality to be published in their present form.
2. DOI and link from "Availability of supporting data and materials" section are not working. Please fix them.

Level of Interest

Please indicate how interesting you found the manuscript: Choose an item.

Quality of Written English

Please indicate the quality of language in the manuscript: Choose an item.

Declaration of Competing Interests

Please complete a declaration of competing interests, considering the following questions:

- Have you in the past five years received reimbursements, fees, funding, or salary from an organisation that may in any way gain or lose financially from the publication of this manuscript, either now or in the future?
- Do you hold any stocks or shares in an organisation that may in any way gain or lose financially from the publication of this manuscript, either now or in the future?
- Do you hold or are you currently applying for any patents relating to the content of the manuscript?
- Have you received reimbursements, fees, funding, or salary from an organization that holds or has applied for patents relating to the content of the manuscript?
- Do you have any other financial competing interests?
- Do you have any non-financial competing interests in relation to this paper?

If you can answer no to all of the above, write 'I declare that I have no competing interests' below. If your reply is yes to any, please give details below.

I declare that I have no competing interests

I agree to the open peer review policy of the journal. I understand that my name will be included on my report to the authors and, if the manuscript is accepted for publication, my named report including any attachments I upload will be posted on the website along with the authors' responses. I agree for my

report to be made available under an Open Access Creative Commons CC-BY license (http://creativecommons.org/licenses/by/4.0/). I understand that any comments which I do not wish to be included in my named report can be included as confidential comments to the editors, which will not be published.

Choose an item.

To further support our reviewers, we have joined with Publons, where you can gain additional credit to further highlight your hard work (see: https://publons.com/journal/530/gigascience). On publication of this paper, your review will be automatically added to Publons, you can then choose whether or not to claim your Publons credit. I understand this statement.

Yes Choose an item.
