# Supplementary material for: U-Limb: A multi-modal, multi-center database on arm motion control in healthy and post-stroke conditions
Source: Gigascience. 2021 Jun 18;10(6):giab043. doi: 10.1093/gigascience/giab043 (PMC8212873; doi:10.1093/gigascience/giab043)
Supplement: giab043_Reviewer_2_Report_Original_Submission — Michael Pereira -- 2/3/2021 Reviewed [file giab043_reviewer_2_report_original_submission.pdf]

Reviewer Report

Title: U-Limb: A multi-modal, multi-center database on arm motion control in healthy and post-stroke conditions.

Version: Original Submission **Date:** 2/3/2021

Reviewer name: Michael Pereira

Reviewer Comments to Author:

In this manuscript, Averta and colleagues describe a dataset, consisting of healthy subjects and stroke patients performing daily activities while kinematic and physiological data were recorded. The dataset also provides fMRI data during executed, imagined and observed hand movements and end-effector kinematics during a virtual peg insertion test. The manuscript is nicely written and the data are well described. I downloaded the UP dataset and it was easy to get the EEG and kinematics data. I believe this is a great initiative, very much needed and I fully agree with the authors that this dataset will constitute a very useful ressource for the neuroscience and robotics communities. I have only a few minor remarks, mainly clarifications that I hope the authors will find useful.

1) There are surely many interesting questions that could be answered using the patient data. However, I think it begs the question of whether a control group can be found in the healthy subject data, which would match on basic demographics (e.g. age, sex and handedness). Maybe the authors could briefly comment on this point.

2) I found it a bit difficult to understand whether some subjects were recorded concurrently with multiple modalities or not. I presume that all subjects in group A were recorded for all four H1, H2, H3 and H4, not that some subjects in group A performed H1, others H2, etc... This might be due to the use of the term "healthy subjects" (Hx; $0 < x < 11$) for what is in my understanding recording modalities (e.g. Table 1, "Data type" section). Maybe this could be briefly clarified.

3) Finally, since data recorded with other protocols than softpro were included, I am curious as to whether the authors plan to consider additional contributions from other groups too. Obviously, I understand that curating such a dataset would require a significant amount of work, however if such inclusions are planned, it might be useful to explicitly state it in the manuscript.

Michael Pereira

Details:

* I would have been interested to have an expected range for the number of trials per subject in the manuscript (I only found it for the fMRI data: 100 trials).
* I did not find information about where the ground electrode (and sometimes the reference electrode) where placed for the EEG.
* I think the only way to get the electrode positions is through plotting the positions from the "chanlocs" structure. Is there maybe an electrode position schema that could be included to the UP dataset?
* I did not find what event the markers correspond to. I assume it is the beginning of the trial, or the movement onset, but it would be nice to have this explicitly stated, also in order to be certain of how to

synchronize different recording signals (e.g. EEG and kinematics).
* "neural hand control" in the abstract sounded a bit weird.

Level of Interest

Please indicate how interesting you found the manuscript: Choose an item.

Quality of Written English

Please indicate the quality of language in the manuscript: Choose an item.

Declaration of Competing Interests

Please complete a declaration of competing interests, considering the following questions:

- Have you in the past five years received reimbursements, fees, funding, or salary from an organisation that may in any way gain or lose financially from the publication of this manuscript, either now or in the future?
- Do you hold any stocks or shares in an organisation that may in any way gain or lose financially from the publication of this manuscript, either now or in the future?
- Do you hold or are you currently applying for any patents relating to the content of the manuscript?
- Have you received reimbursements, fees, funding, or salary from an organization that holds or has applied for patents relating to the content of the manuscript?
- Do you have any other financial competing interests?
- Do you have any non-financial competing interests in relation to this paper?

If you can answer no to all of the above, write 'I declare that I have no competing interests' below. If your reply is yes to any, please give details below.

none

I agree to the open peer review policy of the journal. I understand that my name will be included on my report to the authors and, if the manuscript is accepted for publication, my named report including any attachments I upload will be posted on the website along with the authors' responses. I agree for my report to be made available under an Open Access Creative Commons CC-BY license (http://creativecommons.org/licenses/by/4.0/). I understand that any comments which I do not wish to be included in my named report can be included as confidential comments to the editors, which will not be published.

Choose an item.

To further support our reviewers, we have joined with Publons, where you can gain additional credit to further highlight your hard work (see: https://publons.com/journal/530/gigascience). On publication of this paper, your review will be automatically added to Publons, you can then choose whether or not to claim your Publons credit. I understand this statement.

Yes Choose an item.
